# Evidence-based impact projections of single-dose human papillomavirus vaccination in India: a modelling study

**DOI:** 10.1016/S1470-2045(22)00543-5

**Published:** 2022-11

**Authors:** Irene Man, Damien Georges, Tiago M de Carvalho, Lopamudra Ray Saraswati, Prince Bhandari, Ishu Kataria, Mariam Siddiqui, Richard Muwonge, Eric Lucas, Johannes Berkhof, Rengaswamy Sankaranarayanan, Johannes A Bogaards, Partha Basu, Iacopo Baussano

**Affiliations:** a Early Detection, Prevention and Infections Branch, International Agency for Research on Cancer, World Health Organization (IARC/WHO), Lyon, France; b Amsterdam UMC location Vrije Universiteit Amsterdam, Epidemiology and Data Science, Amsterdam, Netherlands; c Amsterdam Public Health, Amsterdam, Netherlands; d RTI International, New Delhi, India; e Center for Global Noncommunicable Diseases, RTI International, New Delhi, India; f Karkinos Healthcare, Ernakulam, India

## Abstract

**Background:**

Despite the high burden of cervical cancer, access to preventive measures remains low in India. A single-dose immunisation schedule could facilitate the scale-up of human papillomavirus (HPV) vaccination, contributing to global elimination of cervical cancer. We projected the effect of single-dose quadrivalent HPV vaccination in India in comparison with no vaccination or to a two-dose schedule.

**Methods:**

In this modelling study, we adapted an HPV transmission model (EpiMetHeos) to Indian data on sexual behaviour (from the Demographic and Health Survey and the Indian National AIDS Control Organisation), HPV prevalence data (from two local surveys, from the states of Tamil Nadu and West Bengal), and cervical cancer incidence data (from Cancer Incidence in Five Continents for the period 2008–12 [volume XI], and the Indian National Centre for Disease Informatics and Research for the period 2012–16). Using the model, we projected the nationwide and state-specific effect of HPV vaccination on HPV prevalence and cervical cancer incidence, and lifetime risk of cervical cancer, for 100 years after the introduction of vaccination or in the first 50 vaccinated birth cohorts. Projections were derived under a two-dose vaccination scenario assuming life-long protection and under a single-dose vaccination scenario with protection duration assumptions derived from International Agency for Research on Cancer (IARC) India vaccine trial data, in combination with different vaccination coverages and catch-up vaccination age ranges. We used two thresholds to define cervical cancer elimination: an age-standardised incidence rate of less than 4 cases per 100 000 woman-years, and standardised lifetime risk of less than 250 cases per 100 000 women born.

**Findings:**

Assuming vaccination in girls aged 10 years, with 90% coverage, and life-long protection by two-dose or single-dose schedule, HPV vaccination could reduce the prevalence of HPV16 and HPV18 infection by 97% (80% UI 96–99) in 50 years, and the lifetime risk of cervical cancer by 71–78% from 1067 cases per 100 000 women born under a no vaccination scenario to 311 (80% UI 284–339) cases per 100 000 women born in the short term and 233 (219–252) cases per 100 000 women born in the long term in vaccinated cohorts. Under this scenario, we projected that the age-standardised incidence rate threshold for elimination could be met across India (range across Indian states: 1·6 cases [80% UI 1·5–1·7] to 4·0 cases [3·8–4·4] per 100 000 woman-years), while the complementary threshold based on standardised lifetime risk was attainable in 17 (68%) of 25 states, but not nationwide (range across Indian states: 207 cases [80% UI 194–223] to 477 cases [447–514] per 100 000 women born). Under the considered assumptions of waning vaccine protection, single-dose vaccination was projected to have a 21–100% higher per-dose efficiency than two-dose vaccination. Single-dose vaccination with catch-up for girls and women aged 11–20 years was more impactful than two-dose vaccination without catch-up, with reduction of 39–65% versus 38% in lifetime risk of cervical cancer across the ten catch-up birth cohorts and the first ten routine vaccination birth cohorts.

**Interpretation:**

Our evidence-based projections suggest that scaling up cervical cancer prevention through single-dose HPV vaccination could substantially reduce cervical cancer burden in India.

**Funding:**

The Bill & Melinda Gates Foundation.

## Introduction

With approximately 604 000 new cases and 342 000 deaths worldwide in 2020, cervical cancer is the fourth most common cancer in women.^1^ Yet, cervical cancer could be eliminated as a public health problem with appropriate preventive measures.^2^ The licensed prophylactic human papillomavirus (HPV) vaccines protect against infection with vaccine-targeted HPV types, precancerous lesions, and invasive cervical cancer, have demonstrated high efficacy for three-dose and two-dose schedules,^3^, ^4^ and have consistently been shown to be safe.^5^ HPV vaccination is, therefore, one of the key pillars—together with cervical cancer screening and treatment—in the WHO call to eliminate cervical cancer as a public health problem.^6^

**Research in context**

**Evidence before this study**

No formal literature search was done before the start of the study. The very high burden of cervical cancer in India underpins the urgency to upscale key interventions, including vaccination against HPV, cervical cancer screening, appropriate treatment, and palliative care, as recommended in the WHO strategy towards the elimination of cervical cancer as a public health problem. Modelling studies focusing on low-income and middle-income countries suggest that HPV vaccination could help these countries reach the WHO elimination threshold. There is also growing evidence that a similar effect could be achieved with single-dose HPV vaccination. Notably, the IARC India trial with a quadrivalent vaccine, and the Costa Rica Trial with a bivalent vaccine, have shown comparable efficacy between single-dose and multi-dose schedules, with sustained antibody levels for at least 11 years after vaccination. These findings are supported by recent evidence from vaccine trials with randomised single-dose groups, such as the KEN SHE and DoRIS trials. From April, 2022, the WHO Strategic Advisory Group of Experts on Immunization recommended single-dose or two-dose schedules for the primary target group of girls aged 9–14 years and girls and women aged 15–20 years.

**Added value of this study**

We projected the effectiveness and efficiency of quadrivalent single-dose HPV vaccination in India using evidence-based scenarios of single-dose long-term protection derived from the most up-to-date efficacy and immunogenicity data from the IARC India HPV vaccine trial. To our knowledge, we provide the first projections of the effect of HPV vaccination both at national and state-specific levels in India to account for differing cervical cancer risk across the country. We found that single-dose vaccination with long-lasting protection and 90% coverage would prevent up to 78% of cases of cervical cancer among vaccinated birth cohorts across the country, with the greatest relative reduction in cases estimated to occur in states with high cervical cancer incidence. The WHO elimination threshold for cervical cancer (age-standardised incidence rate: 4 cases per 100 000 woman-years) could be achieved by single-dose vaccination across India, whereas the threshold proposed on the basis of standardised lifetime risk (250 cases per 100 000 women born) was projected to only be achieved in 17 (68%) of 25 Indian states . We found single-dose vaccination to be more efficient than two-dose vaccination, preventing at least 21% more cancer cases per dose.

**Implications of all the available evidence**

These projections will help Indian national and subnational health authorities secure resources to introduce a nationwide HPV immunisation programme, monitor the local effect of HPV vaccination, and plan additional preventive interventions, such as cervical cancer screening, in particular in states with high cancer incidence. This study complements existing evidence that single-dose HPV vaccination could be an effective and efficient strategy for cervical cancer prevention in India and other low-income and middle-income settings. Finally, our projections suggest that the higher vaccination efficiency under a single-dose schedule could free up resources for a broader target vaccination population. Catch-up vaccination in girls age 11–15 years and older, possibly up to age 20 years, could be considered.

Among 2005–14 birth cohorts worldwide, the largest burden of cervical cancer is projected in low-income and middle-income countries, with close to a sixth of cases projected to be in India, making it the country with the highest expected burden.^7^ Nevertheless, in India, there is still little access to HPV vaccination and cervical cancer screening.^8^, ^9^

Introduction of HPV vaccination into national immunisation programmes in India, and other low-income and middle-income countries, would be facilitated if a single dose of HPV vaccine were shown to be effective in preventing cervical cancer. Vaccine trials have shown that a single-dose schedule provides non-inferior efficacy against persistent HPV infection compared with multi-dose schedules, with lower, but sustained, antibody levels up to at least 11 years after vaccination.^10^, ^11^ Recently, initial results from a vaccine trial in Kenya^12^ designed to compare the protection of single-dose versus multi-dose HPV vaccination corroborate these findings. Based on these considerations, in April, 2022, the WHO Strategic Advisory Group of Experts on Immunization updated their recommendation on HPV vaccination schedule to a single dose or two doses for the primary target of girls aged 9–14 years and for young women aged 15–20 years.^13^

Here, we model the effect of single-dose quadrivalent HPV vaccination on HPV infection and cervical cancer in India, at the national and state-specific levels. We also compare the effect of single-dose HPV vaccination relative to no vaccination and the alternative of a two-dose vaccination, and also consider the effect of catch-up vaccinations in different age ranges.

## Methods

### Study design and model population

In this modelling study, to project the effect of HPV vaccination in India, we adapted a previously published HPV transmission model^14^ into EpiMetHeos, an agent-based dynamic model (appendix pp 4–5). EpiMetHeos is implemented as an extension of EpiModel (version 2.0.3), an open-source statistical framework that allows simulation of infectious disease transmission on dynamic contact networks.^15^ We modelled transmission of the high-risk HPV types (HPV16, HPV18, HPV31, HPV33, HPV45, HPV35, HPV39, HPV51, HPV52, HPV56, HPV58, HPV59, and HPV68) by combining relevant elements of demographic, sexual behaviour, and HPV natural history.

We divided the model population (n=20 000) into strata by age, sex (male *vs* female), and sexual activity risk group (corresponding to different intensities of sexual contact), which determines their probability of establishing stable and one-off heterosexual partnerships. Transmission of different high-risk HPV types was assumed to be independent and governed by type-specific natural history parameters.

This study adheres to HPV-FRAME, a quality framework for modelled evaluations of HPV-related cancer control (appendix pp 29–31).^16^

### Data sources and model calibration

Because the high-quality data on HPV prevalence we needed to calibrate EpiMetHeos were not available for some states in India, we first identified clusters of states with similar patterns of cervical cancer incidence using Cancer Incidence in Five Continents (CI5; volume XI, for the period 2008–12) and the Indian National Centre for Disease Informatics and Research (NCDIR; for the period 2012–16).^17^, ^18^ Subsequently, on the basis of available HPV prevalence data from two surveys from the states of Tamil Nadu and West Bengal^19^, ^20^ and sexual behaviour data from the Indian National AIDS Control Organisation and the Demographic and Health Survey programme for India for 2015–16,^21^ we calibrated EpiMetHeos to the state of Tamil Nadu to represent the states in the high cancer incidence cluster, and the state of West Bengal to represent the states in the low cancer incidence cluster (appendix pp 6–13). Finally, we projected the effect of HPV vaccination on HPV infection and risk of cervical cancer for Tamil Nadu and West Bengal and extrapolated these projections to other states within each cluster. The states within the low incidence and high incidence groups are listed in the appendix (p 19).

### Model outcomes

Simulations using the 100 best-fitting parameter sets were used to derive the mean and the 10th and 90th percentiles (ie, 80% uncertainty interval [UI]) of the model outcomes. We weighted state-specific outcomes by corresponding population sizes to obtain outcomes nationwide and by high or low cancer incidence cluster. Model outcomes over time were derived for up to 100 years after the start of the vaccination programme, whereas model outcomes by cohorts were derived for the period up to the birth cohort vaccinated 50 years after the start of the vaccination programme.

We assessed reduction in HPV prevalence over time for HPV16 and HPV18; HPV16, HPV18, HPV31, HPV33, and HPV45; and any high-risk HPV type for girls and women aged 15–40 years. We assessed reduction in the cumulative risk of HPV infection by 5-year birth cohort, and for HPV16, HPV18, HPV31, HPV33, and HPV45. Reduction in the first 5-year vaccinated cohort is referred to as a short-term effects. Reduction in the 5-year cohort vaccinated 46–50 years after the start of vaccination is referred to as a long-term effects. A Lexis diagram illustrating the 5-year birth cohorts is shown in the appendix (p 17).

We assessed lifetime risk of cervical cancer (in cases per 100 000 women born) by 5-year birth cohort using a recently published method (appendix p 14, 15).^7^ Baseline lifetime risk (ie, risk in the no vaccination scenario) was based on state-specific data on cervical cancer incidence from CI5 and NCDIR (appendix pp 16, 18–19).^17^, ^18^ For Indian states that were missing data on cervical cancer incidence, we approximated baseline estimates with available data from Indian states with similar sexual behaviour using a footprinting framework (appendix p 6). For risk in the vaccination scenarios, baseline lifetime risk was reduced by EpiMetHeos-based estimates of the relative reduction in the cumulative risk of high-risk HPV infection up to age 40 years, weighted according to the observed type-specific contribution to cervical cancer in India.^22^ We derived the absolute number of cervical cancer cases prevented by scaling the reduction in lifetime risk to UN data on the population size of India for the period 2000–20.^23^

To assess progress towards cervical cancer elimination and to compare countries, we derived the age-standardised incidence rate (ASIR) of cervical cancer on the basis of the world standard population.^24^ Additionally, we assessed an alternative target based on the standardised lifetime risk (SLTR) of cervical cancer,^25^ obtained using all-cause mortality rates of the ten countries with the longest life expectancy, instead of India.^26^ We assessed ASIR by 5-year birth cohort and cross-sectionally over the 100 year time horizon, whereas we assessed the SLTR only by 5-year birth cohort and up to the birth cohort vaccinated 50 years after the start of vaccination. The two indicators differ in that ASIR is the incidence in 100 000 women of different ages, possibly from different cohorts, whereas SLTR is a lifetime cumulation of cases in 100 000 women born into the same birth cohort. For ASIR, elimination of cervical cancer was defined as being below the WHO threshold of 4 cases per 100 000 woman-years,^6^ whereas for SLTR elimination was defined as being below 250 cases per 100 000 women born, as previously defined.^25^ The nationwide baseline risks of cervical cancer were lifetime risk of 1067 cases per 100 000 women born, ASIR of 11 cases per 100 000 woman-years, and SLTR of 1339 cases per 100 000 women born (state-specific baseline risks are in the appendix [p 25]). Additionally, we assessed the attainment of elimination with higher baseline ASIRs, as reported on GLOBOCAN Cancer today (18 cases per 100 000 woman-years)^27^ and GLOBOCAN Cancer over time (15 cases per 100 000 woman-years).^28^

Finally, we assessed the relative per-dose efficiency to prevent cervical cancer, which we defined as the additional proportion of cervical cancer cases prevented per dose under single-dose versus two-dose vaccination. For example, in scenarios with the same efficacy under single-dose and two-dose schedules, relative efficiency would be 100%. In the equation, this reads as ([efficiency of single-dose strategy] / [efficiency of two-dose strategy]) – 100%, where the efficiency of a given strategy is defined as (percentage relative reduction in lifetime risk) / (doses required). Details for computation of model outcomes are in the appendix (pp 14–25).

### Vaccination scenarios

We considered scenarios of no vaccination and single-dose and two-dose quadrivalent HPV vaccination targeting high-risk types HPV16 and HPV18 (and low-risk types HPV 6 and HPV 11). Based on available evidence, we assumed life-long protection by two-dose vaccination throughout all scenarios.^10^ In the base-case scenario, we also assumed single-dose vaccination to provide life-long protection for vaccine-targeted HPV types derived from the near-zero number of breakthrough persistent infections up to 10 years after vaccination and stabilising antibody kinetics, as observed in the IARC India HPV vaccine trial for the quadrivalent vaccine.^11^ On the basis of the same trial, we assumed vaccine efficacy under both single-dose and two-dose schedules to be 95% for HPV16 and HPV18; 9% for HPV 31, HPV33, and HPV45; and 0% for the remaining high-risk HPV types. We modelled routine vaccination in girls aged 10 years with 90% coverage, as recommended in the WHO elimination strategy.^6^ Constant coverage was modelled throughout the simulated 100 year time frame.

In addition to lifelong vaccine protection for both single-dose and two-dose vaccination, referred to hereafter as assumption A, we considered four alternative assumptions (B–E; appendix pp 27–28) with lower initial efficacy and faster waning of protection for single-dose vaccination than in assumption A. We derived these assumptions from the lower bound of efficacy estimated by the IARC India HPV vaccine trial^11^ and by projecting the time until HPV16 and HPV18 antibody levels observed in the trial decreased below predefined thresholds (unpublished). In particular, assumptions B and D were derived from the lowest limit of quantification and the threshold of seropositivity reported to be above the clinical threshold for protection.^29^ Assumptions B, C, and D corresponded to remaining efficacy of approximately 80%, 75%, and 65% for HPV16 and HPV18 at 20 years after vaccination, respectively, with long-term efficacy plateauing above 50% for each of them. The worst-case scenario, assumption E, corresponded with assumption D without cross-protection for HPV31, HPV33, and HPV45.

In combination with the different vaccine protection assumptions, we considered alternative vaccination coverage of 60–100% and catch-up vaccination in women and girls from age 11 up to ages 15, 20, 25, or 30 years, and modelled these catch-up scenarios by their age at the at start of vaccination. The same vaccine efficacy was assumed for all catch-up cohorts, although vaccine effectiveness in catch-up cohorts might be lower because the vaccine can only prevent newly acquired HPV infections. Catch-up vaccination was modelled with 60% coverage or with the same coverage as routine vaccination in the base-case scenario (ie, 90%). As an analysis to study a possible optimal strategy under a scenario with a restricted number of vaccine doses, we compared the scenario of single-dose vaccination catch-up to age 20 years with the scenario with two-dose vaccination without catch-up vaccination. An overview of all vaccination scenarios is shown in the appendix (p 26).

Because access to cervical cancer screening in India is very low, we assumed no screening throughout all scenarios.^8^

### Role of the funding source

The sponsor of the study had no role in study design, data collection, data analysis, data interpretation, or writing of the report.

## Results

**Figure 1:**
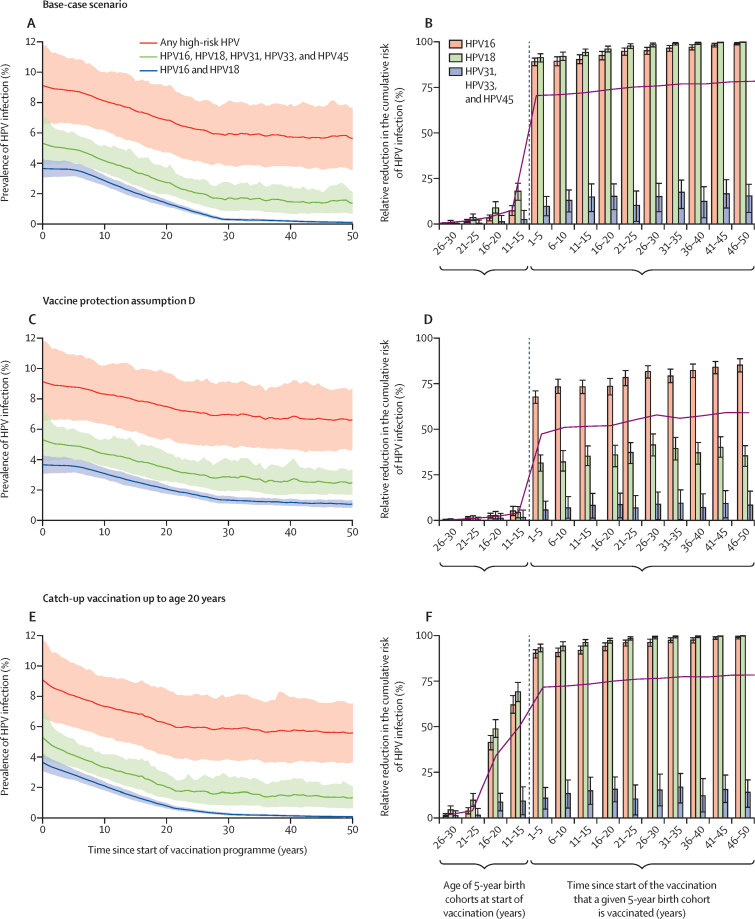
Estimated HPV prevalence over time in girls and women aged 15–40 years (A, C, E) and cumulative risk of HPV infection up to age 40 years, by birth cohort (B, D, F) (A, B) Base-case scenario, with routine vaccination in girls aged 10 years with 90% coverage and life-long vaccine protection by either single-dose or two-dose schedule, without catch-up vaccination. (C, D) Scenario with routine vaccination in girls aged 10 years with 90% coverage and without catch-up vaccination (as in the base-case scenario) but under vaccine protection assumption D for single-dose vaccination. (E, F) Scenario with routine vaccination in girls aged 10 years with 90% coverage and life-long protection, under assumption A for single-dose vaccination (as in the base-case scenario), but with catch-up vaccination in individuals aged up to 20 years that has 60% coverage. (A, C, E) HPV prevalence in girls and women aged 15–40 years over a 50 year time horizon. (B, D, F) Estimated cumulative risk of HPV infection is shown for 5-year birth cohorts; each bar shows 80% uncertainty interval and their weighted mean according to the contribution of each HPV type to the total cervical cancer burden to approximate the relative reduction in lifetime risk of cervical cancer (purple line); the dash vertical line shows the start of the vaccination programme. HPV=human papillomavirus.

In the base-case scenario, assuming life-long protection from HPV16 and HPV18 infection under both single-dose and two-dose schedules, routine vaccination in girls aged 10 years with 90% coverage (without catch-up vaccination) was projected to reduce the prevalence of HPV16 and HPV18 infection by 63% (80% UI 57–69) among women aged 15–40 years in India in the 20 years since the start of the vaccination programme (from 3·7% [3·1–4·3] at baseline to 1·4% [1·1–1·6] at 20 years), and by 97% (96–99) by 50 years after the start of the programme (down to 0·10% [0·05–0·16] at 50 years; figure 1A). For HPV16, HPV18, HPV31, HPV33, and HPV45, reduction in the prevalence of infection after 20 years across all of India from the start of vaccination was projected to be 49% (80% UI 31–63; from 5·3% [3·9–6·9] at baseline to 2·7% [1·9–3·7] at 20 years) and after 50 years was projected to be 75% (60–87; 1·4% [0·7–2·1] at 50 years); whereas, for any high-risk HPV type, the reduction in prevalence was projected to be 25% (1–45) after 20 years (from 9·1% [6·7–11·8] at baseline to 6·8% [4·7–9·1] at 20 years), and 38% (16–61) after 50 years (5·6% [3·6–7·6] at 50 years; figure 1A).

Across all states in India, the reduction in cumulative risk of HPV16 and HPV18 infection up to age 40 years was estimated to be approximately 90% in the short-term cohort (ie, in the first 5-year vaccinated cohort; figure 1B; table 1). In the long-term cohort (ie, in the 5-year birth cohort vaccinated 46–50 years after the start of vaccination), reduction was projected to increase up to almost 100% (figure 1B; table 1). For HPV31, HPV33, and HPV45, the reduction in cumulative risk of infection in the short term was projected to be 10% (80% UI 4–15) and in the long term was 16% (6–22). Generally, the relative reductions in prevalence and cumulative risk of HPV infection were similar across low cervical cancer incidence and high cervical cancer incidence states in India (table 1). All effect estimates on HPV infection are shown in the appendix (pp 32–37).

**Table 1:** Effect of single-dose HPV vaccination on the cumulative risk of high-risk HPV infection and cervical cancer risk in the base-case scenario

		**Relative reduction in cumulative risk of HPV infection (up to age 40 years), %**			**Relative reduction in cervical cancer risk, %**	**Indicators of risk of cervical cancer**			Absolute number of cervical cancer cases prevented*
		HPV16	HPV18	HPV31, HPV33, and HPV45		Lifetime risk, cases per 100 000 women born	Standardised lifetime risk, cases per 100 000 women born	Age-standardised incidence rate, cases per 100 000 woman-years	
**No vaccination scenario**									
India (all states)		NA	NA	NA	NA	1067	1339	11	NA
Low cancer incidence states		NA	NA	NA	NA	922	1158	9·5	NA
High cancer incidence states		NA	NA	NA	NA	1583	1983	16·3	NA
**Base-case scenario**									
India (all states)									
	Short term	89% (87–91)	91% (89–94)	10% (4–15)	71% (69–72)	311 (284–339)	390 (356–425)	3·2 (2·9–3·5)†	471 284
	Long term	99% (98–100)	100% (99–100)	16% (6–22)	78% (78–79)	233 (219–252)	292 (274–316)	2·4 (2·2–2·6)†	517 749
Low cancer incidence states									
	Short term	89% (86–91)	92% (88–94)	10% (3–17)	70% (68–72)	268 (245–293)	337 (308–367)	2·8 (2·5–3·0)†	313 173
	Long term	99% (98–100)	100% (99–100)	17% (4–24)	78% (78–79)	202 (189–217)	253 (237–273)‡	2·1 (1·9–2·2)†	348 964
High cancer incidence states									
	Short term	90% (87–93)	91% (88–93)	8% (3–15)	71% (69–73)	461 (421–503)	577 (527–629)	4·7 (4·3–5·2)	153 823
	Long term	99% (97–100)	100% (99–100)	13% (5–21)	78% (77–79)	346 (324–374)	434 (406–468)	3·6 (3·3–3·8)†	168 989

Data are n or proportion (80% uncertainty interval). Short-term outcomes were assessed using the first 5-year vaccinated birth cohort, and long-term outcomes were assessed using the 5-year birth cohort vaccinated 46–50 years after the start of the vaccination programme. The states within the low incidence and high incidence groups are listed in the appendix (p 19). HPV=human papillomavirus. NA=not applicable.

* Absolute number of cases prevented in respective 5-year birth cohort with 62 million women born in India in 2010–15, and 78% of whom were born in low-incidence states and 22% being born in high-incidence states.

† Below threshold.

‡ Threshold included in the uncertainty interval.

**Figure 2:**
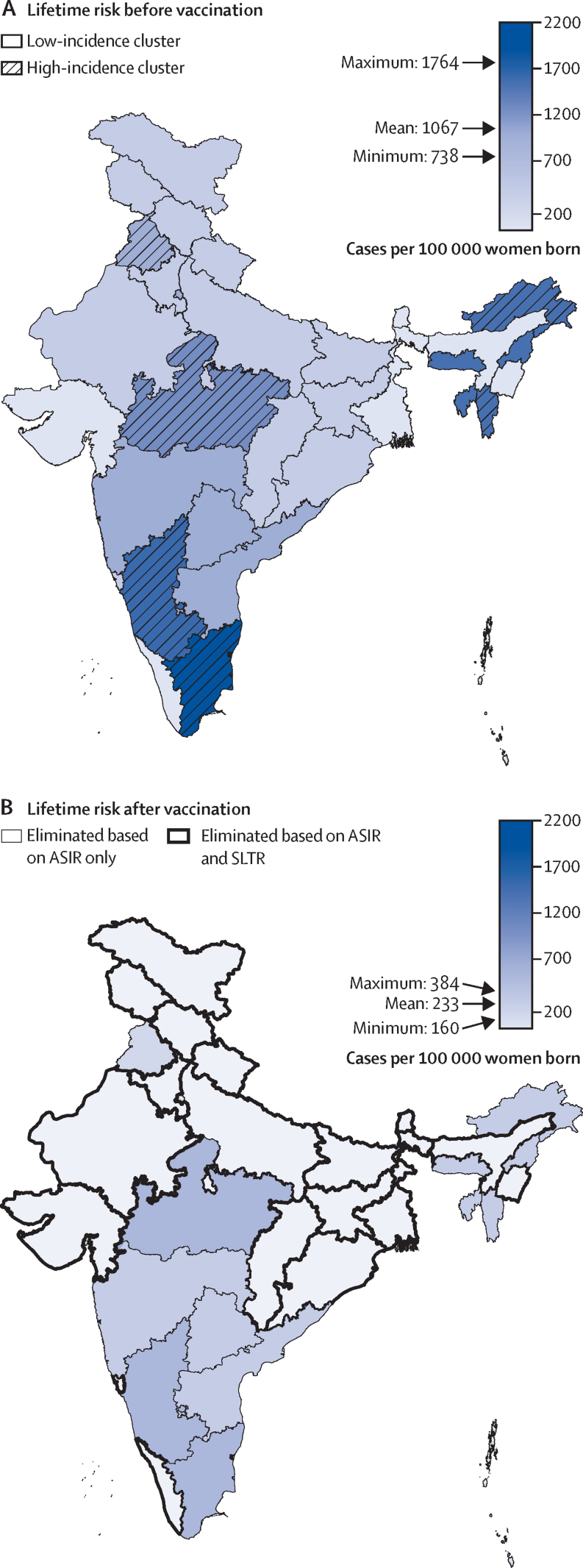
Projected mean lifetime risk of cervical cancer before (A) and after (B) start of vaccination, under the base-case scenario, by state in India Lifetime risk of cervical cancer in cases per 100 000 women born in the base-case scenario (ie, routine vaccination in girls aged 10 years with 90% coverage under life-long vaccine protection by either single-dose and two-dose schedule and without catch-up vaccination). (A) Baseline risk without vaccination. (B) Projections for the 5-year birth cohort vaccinated 46–50 years after the start of the vaccination programme; elimination of cervical cancer based on ASIR only is based on the WHO elimination threshold 4 cases per 100 000 woman-years, whereas elimination based on ASIR and SLTR also incorporates the threshold for SLTR of 250 cases per 100 000 women born. Exact projected values of lifetime risk, SLTR, and ASIR are shown in the appendix (p 39). ASIR=age-standardised incidence rate. SLTR=standardised lifetime risk.

The projected reduction in lifetime risk of cervical cancer was 71% (80% UI 69–72) in the short term, which further increased to 78% (78–79) in the long term (figure 1D, table 1). Estimated relative reduction in cervical cancer risk was similar across low-incidence and high-incidence states, while absolute reduction was higher in states with higher baseline risk (table 1; figure 2). For example, long-term lifetime risk decreased from 1583 to 346 cases (80% UI 324–374) per 100 000 women born in the high cancer incidence states and from 922 to 202 cases (189–217) per 100 000 women born in the low cancer incidence states (table 1).

As a result of the projected reduction in cervical cancer risk, the WHO elimination threshold could already be attained in the low cancer incidence states in the short term and across all states in the long term, with ASIRs ranging between 1·6 cases (80% UI 1·5–1·7) and 4·0 cases (3·8–4·4) per 100 000 woman-years (figure 2; appendix p 39). Due to differential reduction across birth cohorts, the elimination threshold was attained cross-sectionally in the population 60 years after the start of the vaccination programme (appendix p 38). As for SLTR, the threshold of 250 cases per 100 000 women born was projected to be attained in the long term only in 17 (68%) of 25 Indian states, but not nationwide, with SLTRs ranging between 207 cases (80% UI 194–223) and 477 cases (447–514) per 100 000 women born across states (figure 2; appendix p 39). All effect estimates regarding cervical cancer risk are in the appendix (pp 36–51).

**Figure 3:**
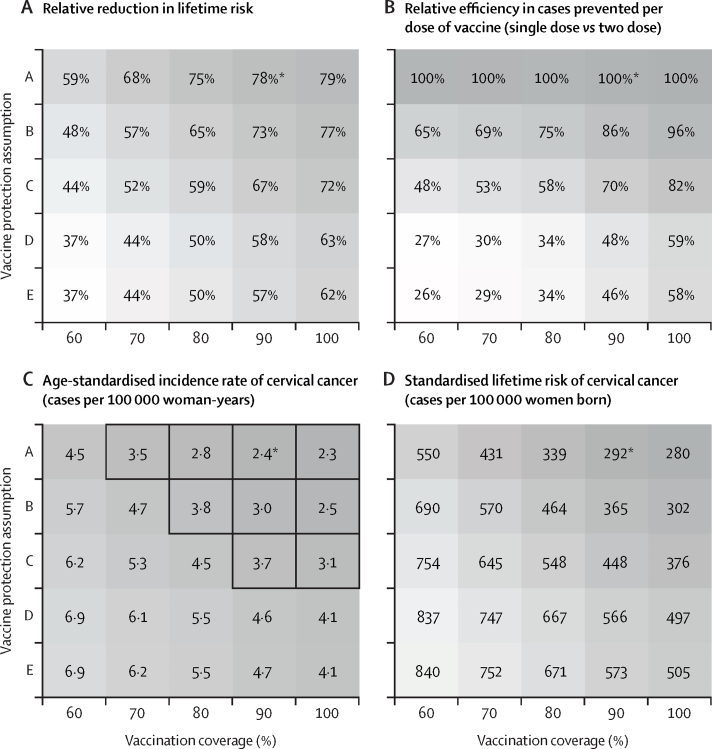
Estimated cervical cancer risk by vaccine protection assumption and coverage Estimates are based on reduction of cervical cancer risk in the long term (ie, the 5-year birth cohort vaccinated 46–50 years after the start of the vaccination programme).(A) Percentage relative reduction in lifetime risk of cervical cancer. (B) Relative efficiency in the number of cervical cancer cases prevented per dose under single-dose versus two-dose vaccination. (C) ASIR rate of cervical cancer; cells with a thick outline indicate that the ASIR projections are below the WHO cervical cancer elimination threshold of 4 cases per 100 000 woman-years; baseline ASIR without vaccination being 11 cases per 100 000 woman-years. (D) Standardised lifetime risk of cervical cancer; none of the cells are below the elimination threshold of 250 cases per 100 000 women born; baseline standardised lifetime risk without vaccination being 1339 cases per 100 000 women born. The setting across all scenarios is routine vaccination in girls aged 10 years without catch-up vaccination. ASIR=age-standardised incidence rate. *Base-case scenario.

We then explored the sensitivity of the model estimates to variations in single-dose protection and vaccination coverage. In our projections, two-dose vaccination with life-long protection led to higher impact than did single-dose vaccination with waning protection (figure 1C, D). Under two-dose vaccination, the long-term reduction in lifetime risk was estimated to be 59–79% with 60–100% vaccination coverage (figure 3A), whereas in the worst-case scenario (vaccine protection assumption E), in which we assumed lower initial efficacy, fast waning protection, and no cross-protection, single-dose vaccination still led to 37–62% reduction in lifetime risk with this range of coverage (figure 3A). Despite the possible lower overall effect, single-dose vaccination was projected to have 26–100% higher per-dose efficiency than two-dose vaccination under all considered vaccine protection assumptions (figure 3B).

Some scenarios with reduced coverage and waning single-dose protection also allowed attainment of the ASIR elimination threshold (figure 3C). For example, with life-long protection, 70% coverage was estimated to be sufficient for elimination. Similarly, with 90% coverage, waning scenario C was estimated to be sufficient for elimination. When using the higher nationwide estimate reported on GLOBOCAN, attainment of the elimination threshold is more difficult but still possible (appendix p 53). By contrast, in our projections, we found that 100% vaccination coverage and life-long protection combined were still not sufficient to attain the SLTR threshold nationwide (figure 3D).

**Figure 4:**
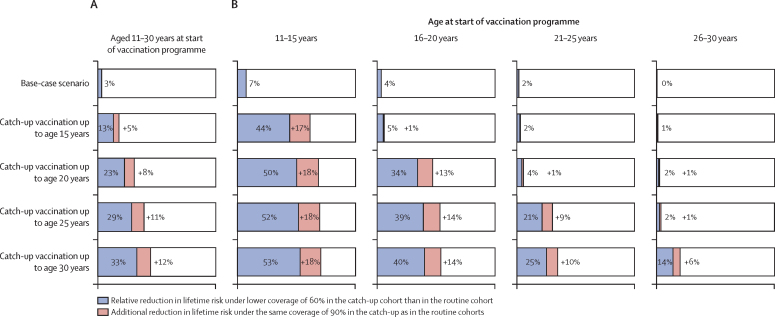
Estimated relative reduction in lifetime risk of cervical cancer with catch-up vaccination, by age group at start of vaccination programme Relative reduction in lifetime risk of cervical cancer in girls and women aged 11–30 years across all states in India at the start of vaccination, unstratified (A) and stratified by age at the start of the vaccination programme (B). Base-case scenario is 90% coverage in routine vaccination cohort of girls aged 10 years, with life-long vaccine protection, without any catch-up. All other scenarios are the same scenario with increasing maximum age of catch-up up to age 15, 20, 25, and 30 years. 80% uncertainty intervals are reported in the appendix (p 52).

In the base-case scenario, without catch-up vaccination, women beyond the eligible age of routine vaccination benefited slightly from routine vaccination in younger cohorts. Among women aged 11–30 years at the start of the vaccination programme, we projected that the nationwide lifetime risk of cervical cancer would be reduced by 3% (80 UI 2–4; figure 4A), with the highest reduction of 7% (5–10) being in the youngest age group, 11–15 years (figure 4B; appendix p 52).

Compared with the base-case scenario, we found that including catch-up vaccinations accelerated and increased the estimated effect of vaccination on HPV infection and cervical cancer risk (figure 1E, F). Extending catch-up to those aged 15, 20, 25, and 30 years successively, with 60% coverage, increased the projected reduction in lifetime risk among all women aged 11–30 years at the start of the vaccination programme from 3% up to 13% (80% UI 12–14) to 33% (31–35) (figure 4A). With 90% coverage in the catch-up cohorts, the estimated reduction in lifetime risk further increased to 18% (17–19) to 45% (43–46; figure 4A; appendix p 52).

Although expanding the age range of catch-up in our projections increased the total effect of vaccination, the marginal gain was diminished because of increased sexual exposure at older ages. For instance, catch-up vaccination in girls aged 11–15 years with 60% coverage could reduce their lifetime risk by 53% (80% UI 50–57), whereas the same catch-up in women aged 26–30 years is estimated to reduce lifetime risk by only 14% (12–16; figure 4B; appendix p 52).

Finally, we compared two alternative strategies allocating the same number of doses over a 10-year period: targeting only ten routine cohorts with two-dose vaccination without catch-up, or reallocating the second dose from the routine cohorts to include catch-up in the ten birth cohorts aged 11–20 years at the start of the vaccination programme. We estimated that the two-dose strategy without catch-up (and 90% coverage in the routine cohort) would only reduce the lifetime risk of cervical cancer in the 20 birth cohorts by 38% (80% UI 37–39; table 2), whereas, under life-long vaccine protection assumption A, the single-dose strategy with catch-up increased the reduction in lifetime risk to an estimated 57% (56–58) under 60% catch-up coverage, and 65% (64–66) under 90% catch-up coverage (table 2). Correspondingly, the single-dose strategy was estimated to reach 80% higher per-dose efficiency than two-dose vaccination under 60% coverage and 71% higher per-dose efficiency under 90% coverage. Estimated relative efficiency was 21–80% in all considered scenarios (table 2).

**Table 2:** Effect of single-dose strategy with catch-up versus two-dose strategy without catch-up on cervical cancer risk, by vaccine protection assumption scenario

		**Relative reduction in lifetime risk, %**			Relative efficiency in cases prevented per dose (single dose *vs* two dose)*	**Absolute number of cervical cancer cases prevented**†
		Routine cohort‡	Candidate catch-up cohort§	Routine and candidate catch-up cohorts combined		
**Two-dose vaccination without catch-up**						
A (life-long protection)		71% (69–72)	6% (4–7)	38% (37–39)	NA	1 055 193
**Single-dose vaccination with catch-up**						
60% coverage of catch-up vaccination						
	A (life-long protection)	72% (71–73)	42% (40–44)	57% (56–58)	80%	1 582 790
	B	64% (62–66)	38% (35–40)	51% (49–53)	61%	1 416 181
	C	58% (56–61)	33% (30–36)	46% (44–48)	45%	1 277 339
	D	51% (49–53)	28% (26–31)	40% (38–41)	26%	1 110 730
	E	50% (48–52)	28% (26–30)	39% (37–40)	23%	1 082 962
90% coverage of catch-up vaccination						
	A (life-long protection)	73% (72–74)	58% (55–60)	65% (64–66)	71%	1 804 936
	B	66% (64–67)	52% (49–54)	59% (57–60)	55%	1 638 327
	C	60% (58–62)	47% (45–49)	53% (52–55)	39%	1 471 717
	D	52% (50–54)	40% (38–42)	46% (44–48)	21%	1 277 339
	E	51% (49–53)	40% (37–42)	46% (44–47)	21%	1 277 339

Data are n, proportion, or proportion (80% uncertainty interval). Vaccine protection assumptions A–E are described in the appendix (pp 27–28). HPV=human papillomavirus. NA=not applicable.

* Under 90% coverage in the catch-up cohorts, number of doses required in routine and catch-up cohorts combined is the same for both strategies, whereas under 60% coverage in the catch-up cohorts, number of doses required in routine and candidate catch-up cohorts combined is 1·2 times higher for the two-dose routine-only strategy than the single-dose strategy.

† Absolute number of cases prevented in the routine and catch-up birth cohorts, combined with the total number of women born in India in 2000–20, which was estimated to be 260 million.

‡ The first ten routine vaccination birth cohorts.

§ The ten birth cohorts aged 11–20 years at start of vaccination that could be vaccinated as part of catch-up programme.

## Discussion

On the basis of data on Indian cervical cancer epidemiology and the IARC India HPV vaccine trial, we projected the effect of single-dose quadrivalent HPV vaccination on HPV infection and cervical cancer risk in India and explored its advantages compared with two-dose vaccination. Assuming the same long-lasting efficacy of single-dose and two-dose vaccinations, we found that vaccination by either dose schedule in girls aged 10 years could reduce lifetime risk of cervical cancer in future vaccinated birth cohorts by up to 79% in future vaccinated birth cohorts compared with no vaccination. As a result, HPV vaccination could prevent close to 1 million cases of cervical cancer in the lifetime of the 120 million Indian girls currently aged 10 years and younger.

Although two-dose HPV vaccination might result in a greater reduction in risk of cervical cancer, we projected that single-dose HPV vaccination would be 21–100% more efficient in terms of cases prevented per dose under the considered scenarios of single-dose vaccine protection. Single-dose vaccination could potentially free up resources for catch-up vaccination, accelerating reduction in the burden of cervical cancer. In a separate health economics analysis (unpublished), we found that single-dose HPV vaccination with catch-up vaccination in girls aged 11–20 years could be cost-effective under the WHO recommended cost-effectiveness threshold of one-times gross domestic product per capita.^30^ This finding is consistent with a previous multi-country modelling study on low-income and middle-income settings, including India.^31^

Our projections also support previous modelling studies^2^, ^31^, ^32^ that found high coverage of vaccination against HPV16 and HPV18 in girls aged 10 years could reduce the burden of cervical cancer in India considerably, and enable the country to reach the WHO elimination target even with single-dose vaccination. We projected that, under a single-vaccination scenario, the elimination target could be reached nationwide in approximately 60 years. To accelerate the reduction in the incidence of cervical cancer, cervical screening should also be considered for older cohorts in whom vaccination might no longer be effective, and who might contribute to the burden in the future. Additionally, our projections indicate that elimination could still be hard to achieve in the long term for Indian states with high baseline cervical cancer risk. For these states, it would be important to ensure high vaccination coverage and consider additional preventive measures such as cervical screening and higher valency vaccines, which are also likely to be efficacious under a single-dose schedule.^12^ Furthermore, screening and higher valency vaccines both help to reduce the burden of cervical cancer attributable to high-risk HPV types other than HPV16 and HPV18. Future modelling studies could provide important guidance on whether Indian states should consider either measure alone or adopt an optimal combination of both measures.

To complement our assessment of progress towards elimination, we also provide effect projections in terms of lifetime risk indicators. As recently suggested,^25^ using SLTR to define public health targets allows comparison of actual or projected outcomes against the highest observed life expectancy standards, and provides an alternative approach to standardisation that does not rely on outdated world population age distribution.^25^ We found the SLTR threshold of 250 cases of cervical cancer per 100 000 women born to be more stringent than the WHO threshold, and projected this threshold to be attainable in only 17 of 25 Indian states under single-dose HPV vaccination strategies. This finding is in line with our expectations, because the SLTR threshold is based on the level of risk of cervical cancer projected in a setting with elimination of all cervical cancer due to HPV16 and HPV18 and cervical cancer screening in place.^25^ Although this threshold provides long-term vision, more realistic intermediate targets might also be helpful. Additionally, we provided projections in terms of non-standardised lifetime risk, which are easier to interpret than the standardised indicators and hence might be more valuable in guiding local health policy decisions.

A major strength of this modelling study is the amount of local data and evidence that make our results context specific. First, we used data from multiple HPV prevalence surveys, and state-specific data on cervical cancer incidence and sexual behaviour in our model to capture the varying epidemiology of cervical cancer across India. To our knowledge, we are the first group to project the effect of HPV vaccination in India with state-specific granularity. Second, we explored uncertainty on single-dose protection duration by using efficacy and immunogenicity data from the IARC India HPV vaccine trial (unpublished),^11^ which currently provides the best data on single-dose protection because of its long follow-up and near-random assignment of participants to different dose schedules.^10^, ^11^ Specifically, we projected a plateau efficacy of 50% in the long term, in the worst-case assumption, on the basis of a seropositivity threshold, which is probably higher than that needed for protection.^29^ This assumption differs from previous studies, which mostly assumed protection to disappear in 20 to 40 years after vaccination.^31^, ^33^

Our study has some limitations. First, our estimates regarding cervical cancer elimination depend on the assumed baseline risk of cervical cancer. The nationwide baseline risk reported here (11 cases per 100 000 woman-years) is lower than that in GLOBOCAN (18·0 cases per 100 000 woman-years),^27^ because we also included data from Indian states with relatively low cancer incidence that are not in CI5. Using GLOBOCAN estimates would make reaching the WHO elimination threshold more difficult, but should still be possible under high vaccine coverage and efficacy.^2^, ^31^ Second, we approximated the relative effect of HPV vaccination on risk of cervical cancer with relative effect on cumulative risk of high-risk HPV infection up to age 40 years. Stabilisation of the relative effect beyond age 40 years was verified with the model estimates. Furthermore, truncation at this age was motivated by the reduced likelihood of infections acquired beyond this age to progress to cancer due to the hormonal patterns after menopause, and the shorter time to develop cervical cancer in this older age group.^34^, ^35^ Finally, our results are subject to uncertainty on changes in sexual behaviour over time. Although the incidence of cervical cancer has been decreasing in India, changing sexual behaviours related to an emerging economy (eg sexual debut at earlier ages and marriage at older ages) might make control of cervical cancer more challenging than projected.^9^

In summary, in this modelling study, we projected that, under a range of plausible conditions of coverage and vaccine protection, single-dose HPV vaccination could be more efficient in decreasing the burden of cervical cancer in India than a two-dose strategy, and could eliminate cervical cancer as a public health problem. Emerging evidence from ongoing studies and new vaccine trials will be crucial to periodically reassess the potential benefits of single-dose vaccination and whether booster vaccination is needed at an older age.^10^, ^11^, ^12^ In addition to the technological aspects, the success of HPV vaccination depends on securing political will and public acceptance.^9^ Ultimately, if successfully implemented, a national HPV vaccination programme is expected to not only improve the health of the Indian population, but would also generate useful lessons for implementing HPV vaccination in other low-income and middle-income countries.

## Data sharing

External researchers can make written requests to the IARC for sharing of data regarding the IARC India HPV vaccine trial. Requests will be assessed on a case-by-case basis in consultation with lead investigators and co-investigators. A brief analysis plan and data request will be required and reviewed by the investigators for approval of data sharing. When requests are approved, anonymised data will be sent electronically in password protected files. All data sharing will abide by rules and policies defined by the involved parties. Data sharing mechanisms will ensure that the rights and privacy of individuals participating in research will be protected at all times. Data are from public sources that are listed in the appendix (pp 11–13). The model code is available from the authors on request.

## Declaration of interests

JB has received support to his institution from IARC/WHO outside of the submitted work. All other authors declare no competing interests.
