# Supplementary material for: Evidence-based impact projections of single-dose human papillomavirus vaccination in India: a modelling study
Source: Lancet Oncol. 2022 Nov;23(11):1419–29. doi: 10.1016/S1470-2045(22)00543-5 (PMC9622421; doi:10.1016/S1470-2045(22)00543-5)
Supplement: Supplementary appendix [file mmc1.pdf]

THE LANCET
Oncology

Supplementary appendix

This appendix formed part of the original submission and has been peer reviewed. We post it as supplied by the authors.

Supplement to: Man I, Georges D, de Carvalho TM, et al. Evidence-based impact projections of single-dose human papillomavirus vaccination in India: a modelling study. *Lancet Oncol* 2022; published online Sept 26. https://doi.org/10.1016/S1470-2045(22)00543-5.

Appendices

Contents

List of tables 2
List of figures........ 3
A.1. HPV Transmission model 4
A.1.1. Demography........ 4
A.1.2. Sexual contact behaviour 4
A.1.3. HPV natural history........ 4
A.2. Model calibration........ 6
A.2.1. Footprinting framework 6
A.2.2. Calibration to sexual behaviour data........ 7
A.2.3. Calibrating to HPV prevalence data........ 9
A.3. Computation of model outcomes........ 14
A.3.1. Prevalence of HPV infection........ 14
A.3.2. Cumulative risk of HPV infection........ 14
A.3.3. Life-time risk of cervical cancer 14
A.3.4. Standardised life-time risk of cervical cancer 15
A.3.5. Age-standardised incidence rate of cervical cancer........ 15
A.3.6. Relative per-dose efficiency........ 15
A.3.7. Aggregating model outcomes........ 15
A.4. Vaccination scenarios........ 26
A.4.1. Overview of vaccination scenarios 26
A.4.2. Vaccine protection assumptions 27
A.5. HPV-FRAME checklist........ 29
.... 30
B.1. Supplementary results in tables........ 32
B.2. Supplementary results in figures........ 52
Reference........ 54

List of tables

Table A1. List of model parameters
Table A2. Overview of available cancer incidence data from local registries by Indian state
Table A3. Age-specific cervical cancer incidence data by Indian state
Table A4. Scaling parameters for prevalence of groups of HPV types
Table A5. Female mortality rate of India and ten countries with the highest life expectancy
Table A6. Type-specific contribution of HPV types in cervical cancer
Table A7. Standard world population
Table A8. Female population size by Indian state
Table A9. Pre-vaccination risk of cervical cancer by Indian state
Table A10. Overview of vaccination scenarios
Table A11. Overview of parameters under different vaccine protection assumptions
Table A12. HPV-FRAME checklist

Table B1. Relative reduction in HR HPV prevalence over time in the base-case scenario
Table B2. Relative reduction in nationwide HR HPV prevalence over time across single-dose protection assumptions
Table B3. Relative reduction in nationwide cumulative risk of HR HPV infection and cervical cancer risk by cohort across single-dose protection assumptions
Table B4. Nationwide age-standardised incidence rate of cervical cancer over time in the base-case scenario
Table B5. Cervical cancer risk in the long-term across Indian states in the base-case scenario
Table B6. Age-standardised incidence rate of cervical cancer in the long-term across single-dose protection assumptions
Table B7. Standardised life-time risk of cervical cancer in the long-term across single-dose protection assumptions
Table B8. Life-time risk of cervical cancer in the long-term across single-dose protection assumptions
Table B9. Relative reduction in life-time risk of cervical cancer by maximum age of catch-up

List of figures

Figure A1. Structure of HPV natural history in EpiMetHeos
Figure A2. Model fit of the target statistics of sexual contact behaviour
Figure A3. Model fit of the type-specific HPV prevalence data
Figure A4. Age-specific cervical cancer incidence data by Indian state
Figure A5. Mean of age-specific cervical cancer incidence of the cluster of Indian states
Figure A6. Lexis diagram showing the 5-year birth cohorts targeted by HPV vaccination
Figure A7. Vaccine efficacy by HPV type for different vaccine protection assumptions for single-dose vaccination
Figure A8. Immunogenicity data of IARC India vaccine trial and thresholds of protection

Figure B1. Age-standardised incidence rate of cervical cancer under alternative baseline values

Appendix A

A.1. HPV Transmission model

We adapted a previously published HPV transmission model [1] into an stochastic agent-based dynamic model, EpiMetHeos. EpiMetHeos is an extension of EpiModel [2] an open-source statistical framework that allows simulation of infectious disease transmission on dynamic contact networks.

A.1.1. Demography

The model considers an open population in which individuals enter at the age of 10 years and exit due to death with maximum age of 100 years. Each individual is characterized by the two demographic characteristics, age and sex, denoted by $a \in [10{,}100]$ and $g \in \{W, M\}$, respectively. The entire age range is further stratified into the following age groups: $ageg \in \{10-14, 15-19, 20-24, 25-29, 30-49, 50-59, 60-99\}$.

Individuals die according to sex- and age-specific probabilities $m_{g,age}$. The number of new individuals born into the population per time step is given by b, which is set to a value that keeps the total population constant over time and with 50%-50% of female and male new-borns. A constant age-specific population size distribution corresponding to $m_{g,age}$ is achieved through the simulation of a burn-in period.

A.1.2. Sexual contact behaviour

Two dynamic contact networks of sexual partnerships are modelled among the model individuals: one for stable partnerships and one for one-off partnerships. Stables partnerships represent marital partnerships and one-off partnerships outside marriage. Both networks are constructed using EpiModel's implementation of the Separable Temporal Exponential-family Random Graph Models (STERGM).[2] An STERGM is characterised by formation and dissolution probabilities of partnerships. At each time step, partnerships that were present at the previous time step can be dissolved, and new partnership can be formed between individuals that were not connected at the previous time step.

Formation of partnerships is only allowed between opposite sex individuals to model heterosexual networks. Formation probabilities of stable as well as one-off partnerships depend on the individuals' sex, age group and the assigned risk group of sexual activity. Each individual is assigned to one of the five risk groups of sexual activity, denoted by $riskg \in \{1,2,3,4,5\}$, which determines which type of partnerships he/she is allowed to form as follows:

- $riskg = 1$: no stable, no one-off,
- $riskg = 2$: only stable,
- $riskg = 3$: both stable and one-off,
- $riskg = 4$: only one-off,
- $riskg = 5$: only one-off; in women, this risk group represents female sex workers.

The risk group of an individual is assigned randomly at birth according to a sex-specific multinomial distribution $p_{g,riskg}$ and remains unchanged for the rest of the individual's lifespan. The dissolution probability differs between stable and one-off partnerships but is the same for all individuals.

Sexual behaviour is further characterized based on parameters regarding the number of sex acts within established partnerships. At each time step, sex acts may occur within a stable partnership. The number of sex acts is randomly generated according to a Poisson distribution with mean r^{main}. Within a one-off partnership, the number of sex acts $r^{one-off}$ is exactly one. The parameter values regarding sexual contact behaviour were fixed or obtained through a calibration step described in Section A.2.2.

A.1.3. HPV natural history

HR HPV types are assumed to be transmitted independently, governed by type-specific natural history parameters, including the probability of transmission, duration of infection and natural immunity. Transmission of all HR HPV types are assumed to follow the "Susceptible-Infected-Susceptible" dynamics in men and the "Susceptible-Infected-Removed/Immune-Susceptible" dynamics in women (**Figure A1**). Effects of HIV, smoking, and use of contraceptives, as risk factors of HPV transmission and cervical cancer progression, were not included in the model. We chose not to include them in the model. For HIV infection, this choice was based on the low HIV prevalence in India.[3] As for smoking and the use of contraceptives, the choice was based on the lack of data.

All individuals enter the population being susceptible. At each time step, an unvaccinated susceptible individual having l sex acts with an individual infected with type i has a probability of $1-(1-\beta_i)^l$ becoming infected, where β_i is the probability of transmission per sex act. If vaccinated and vaccine protection is successfully induced, the probability of transmission for a vaccine-targeted type, either a vaccine type or a cross-protective type, becomes zero. In other words,

we assumed an all-or-nothing working mechanism for vaccine protection. When vaccine-induced protection is lost, the probability of transmission returns to $1-(1-\beta_i)^l$.

The duration of infection follows a type-specific distribution with six parameters $\gamma_i, \eta_i, \delta_{i,1}, \delta_{i,2}, \nu_{i,1}, \nu_{i,2}$. This distribution describes the process through CIN0, CIN1, regressive CIN2/3, and non-regressive CIN3 stages as modelled in an extensively validated cervical cancer progression model.[4,5] In particular, non-regressive CIN3 here represents the part of HPV infections that will persist and progress to cancer. For those HPV infections that do not persist, upon clearance, men become susceptible and women become removed/immune. The duration of natural immunity in women follows an exponential distribution type-specific rate μ_i, after which women also become susceptible again. The values of the natural history parameters were fixed or obtained through a calibration step described in Section A.2.3.

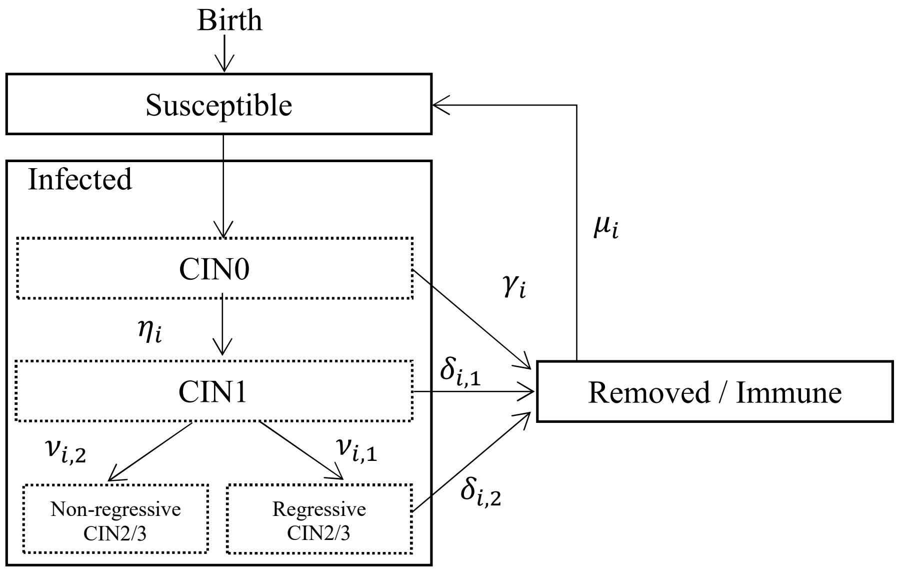

Figure A1. Structure of HPV natural history in EpiMetHeos.

A.2. Model calibration

A.2.1. Footprinting framework

Since high-quality data about HPV prevalence and cervical cancer incidence data essential to project the impact of HPV vaccination are not available for each Indian state, the Footprinting framework was used to approximate missing data and extrapolate impact projections. The Footprinting framework is described in more details in a separate manuscript.[6] The framework consists of three steps: clustering, classification, and projection. These steps are briefly described below.

Clustering step
In the clustering step, Indian states with available cervical cancer incidence data were clustered based on their similarity in patterns of age-specific cervical cancer incidence. Sources for cervical cancer incidence data were volume XI of Cancer Incidence in Five Continent (CI5) and the 2012-2016 Report of National Cancer Registry Programme by National Centre for Disease Informatics and Research (NCDIR).[7,8] See **Table A2** for an overview of Indian states (or groups of states) with available cancer incidence data from local registries. Of the 25 Indian states, 14 states have local registries and 11 do not. Whenever a registry was both present in CI5 and NCDIR, only the data by CI5 were used.

A Poisson-regression-based CEM clustering algorithm was used to cluster the age-specific cervical cancer incidence curve. Details of the clustering algorithm are described in Appendix S1 of the separate manuscript, and its results are reported in the main text of the separate manuscript.[6] We identified one cluster with high cancer incidence and one with low cancer incidence. See also column “cluster” of **Table A3** for the assigned cluster of each Indian state. In this table, the Indian states with cervical cancer incidence, hence involved in this clustering step, are indicated by “extracted” in the column “source”.

Classification step
The remaining Indian states without cervical cancer incidence data were classified to the identified clusters of Indian states with similar patterns of cervical cancer incidence based on similarity in sexual behaviour data from the behaviour surveillance survey by the National AIDS Control Organization of India.[9]

Random forest was used for classification. Details of the classification method are described in Appendix S2 of the separate manuscript, and the results are reported in the main text of the separate manuscript.[6] See also column “cluster” of **Table A3** for the assigned cluster of each Indian state. In this table, the Indian states without cervical cancer incidence, hence involved in this classification step, are indicated by “inferred” in the column “source”.

Projection step
In the projection step, baseline (i.e., in the scenario without vaccination) HPV prevalence and cervical cancer incidence were approximated, based on the available data within each cluster. For HPV prevalence, high-quality type- and age-specific HPV prevalence data were only available for Tamil Nadu and West Bengal, the former being in the “high” and the latter in the “low” incidence cluster.[10,11] As for cervical cancer incidence data, approximation was based on the mean age-specific incidence within each cluster. See **Figure A5** and **Table A3** for mean values. From the approximated Indian state-specific baseline cervical cancer incidence, we then derived cervical cancer risk in terms of three indicators, life-time risk (LTR), standardized life-time risk (SLTR), and age-standardized incidence rate (ASIR), according to the methodology described in **Sections A.3.3**, **A.3.4**, and **A.3.5**, respectively. See **Table A9** for baseline cervical cancer risk by the three indicators.

HPV prevalence and cervical cancer risk under vaccination scenarios were obtained as follows. The HPV transmission model, EpiMetHeos, was calibrated to these two representative Indian states (Tamil Nadu and West Bengal) according to the procedure described in **Section A.2.2.** Using the two obtained models, we then simulated different vaccination scenarios and obtained estimates of relative reduction in HPV prevalence and HPV infection risk. Finally, these estimates of relative reduction were applied to the previously obtained baseline values of HPV prevalence and cervical cancer risk to derive the values under vaccination scenarios.

A.2.2. Calibration to sexual behaviour data

The model was calibrated to the two representation states: West Bengal and Tamil Nadu. Model calibration consists of two steps. In the first step, the component of the model simulating the dynamic contact networks was calibrated to the sexual behaviour data without considering the component concerning the natural history of HPV yet. Four sources of sexual behaviour data were used:

- The Demographic and Health Survey (DHS) programme, to provide information on stable partnerships in West Bengal and Tamil Nadu.[12]
- National Behavioural Surveillance Survey 2006 by The National AIDS Control Organisation (NACO), to provide information on one-off partnerships in West Bengal and Tamil Nadu.[9]
- Publication by Gaffey et al., "Male use of female sex work in India: a nationally representative behavioural survey", to provide information on proportion of men with stable partnership that also have one-off partnerships in West Bengal and Tamil Nadu.[13]
- Publication by Vandepitte et al, "Estimates of the number of female sex workers in different regions of the world", to provide information on one-off partnerships across India.[14]

First, the probabilities of being assigned to different risk groups of sexual activity were fixed. See **Table A1** for the fixed values and the justification. Subsequently, formation and dissolution probabilities of the dynamic sexual contact networks were fitted to a set of target statistics using the netest function of EpiModel. The set of target statistics was:

- Stable partnerships:
  - Sex- and age-group-specific population proportion with stable partnerships $d_{g,ageg}^{stable}$
  - Sex- and risk-group-specific population proportion with stable partnerships $d_{g,riskg}^{stable}$
  - Overall population proportion with stable partnerships d^{stable}
  - Mean age difference in stable partnerships κ^{stable}
  - Mean spread of absolute age difference in stable partnerships ω^{main}
  - Mean duration of stable partnerships s^{stable}
- One-off partnerships:
  - Sex- and age-group-specific mean degree of one-off partnerships $d_{g,ageg}^{one-o}$
  - Sex- and risk-group-specific mean degree of one-off partnerships $d_{g,riskg}^{one-of}$
  - Overall mean degree of one-off partnerships d^{one-of}
  - Mean duration of one-off partnerships $s^{one-off}$

See **Table A1** for the values of the target statistics and the corresponding data sources used. See **Table A1** also for the fixed parameters and the corresponding data sources used. See **Figure A2** for the fit to the target statistics. Note that assortativeness of sexual contact by age- and risk-groups are results of the fitting process.

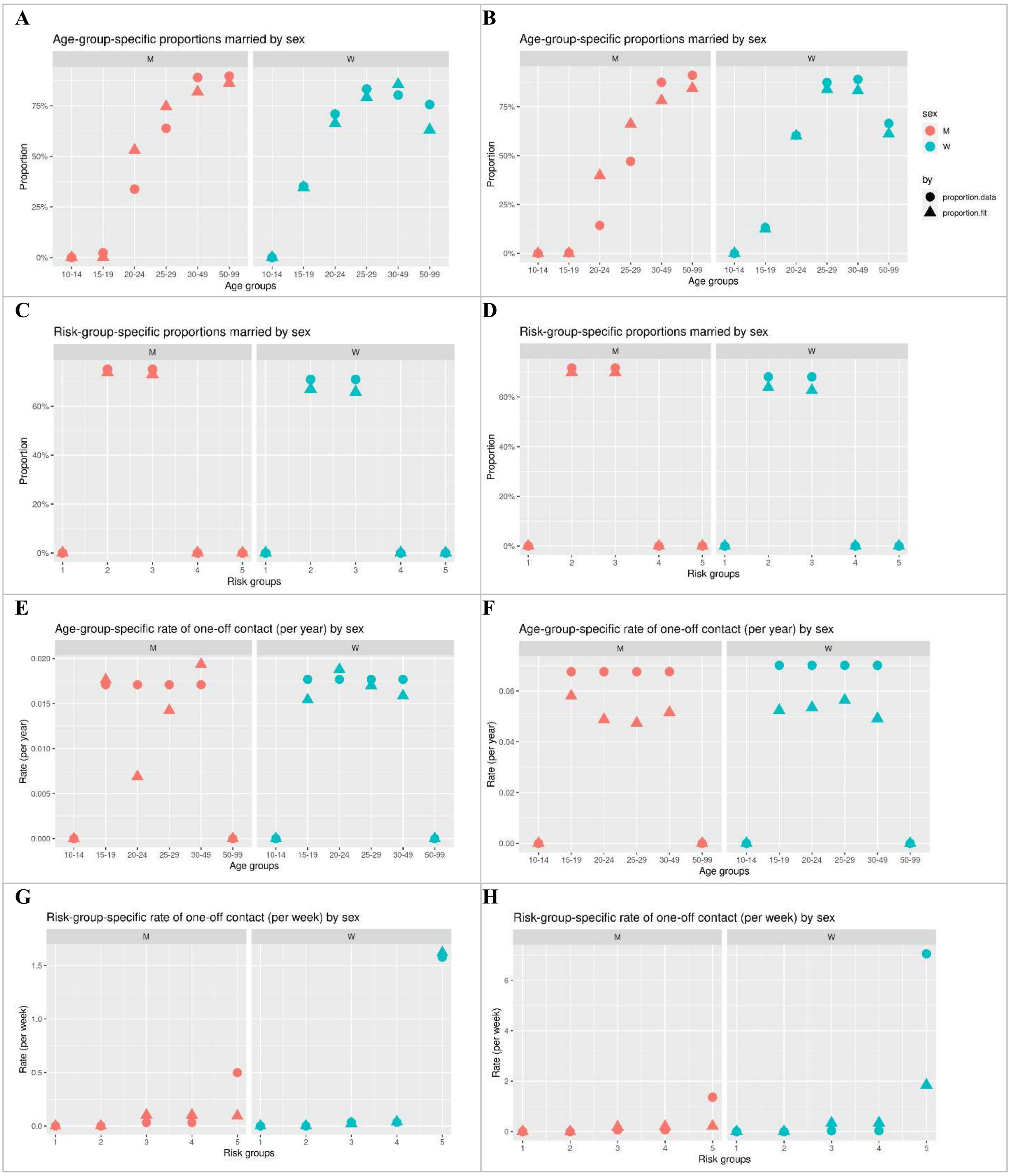

Figure A2. Model fit of the target statistics of sexual contact behaviour. Left column: West Bengal. Right column: Tamil Nadu. Circle: targets derived from data. Triangle: fit by model.

A.2.3. Calibrating to HPV prevalence data

In the second step, the entire model was calibrated, including the component concerning the natural history of HPV. This was done by fitting the model HPV prevalence among sexually active women to HPV prevalence data from two surveys among married women in Tamil Nadu and West Bengal.[10,11] To obtain consistent type-specific HPV natural history parameters between Tamil Nadu and West Bengal, we scaled the relative type-specific prevalence of Tamil Nadu to West Bengal. The relative prevalence of HPV 16/18 between Tamil Nadu and West Bengal was used as the scaling factor.

Due to the low prevalence of some HR HPV types, we used the average prevalence of the following three groups of HR HPV types as target prevalence:

- HPV 16
- HPV 18
- HPV 31/33/45/35/39/51/52/56/58/59/68

Note that for the subsequent simulation of the vaccination scenarios, the calibrated parameters based on average prevalence of HPV 31/33/45/35/39/51/52/56/58/59/68 were then used to model the cross-protective types HPV 31/33/45 and the other HR HPV types HPV 35/39/51/52/56/58/59/68, which are referred to as "*cross*" and "*other*".

The parameter values regarding the type-specific progression, clearance rates and waning rates of natural immunity were fixed to those estimated for the extensively validated cervical cancer progression model.[4,5] For HPV 31/33/45/35/39/51/52/56/58/59/68, we derived the mean values of the type-specific estimates.

The parameter values obtained in this calibration step regard type-specific transmission probabilities and one-off partnership underreporting rate. Using 2500 parameter sets that were uniformly generated from the range (**Table A1**), we selected 100 best-fitting parameter sets. Model fit was evaluated based on log-likelihood of the observed HPV prevalence data given the simulated HPV prevalence under a binomial distribution. For each parameter set, log-likelihood was computed at each year in the last 50 years of 250 years of simulation. The maximum and mean log-likelihood across these years were used as the two summary statistics. See **Figure A3** for the fit to the HPV target prevalence.

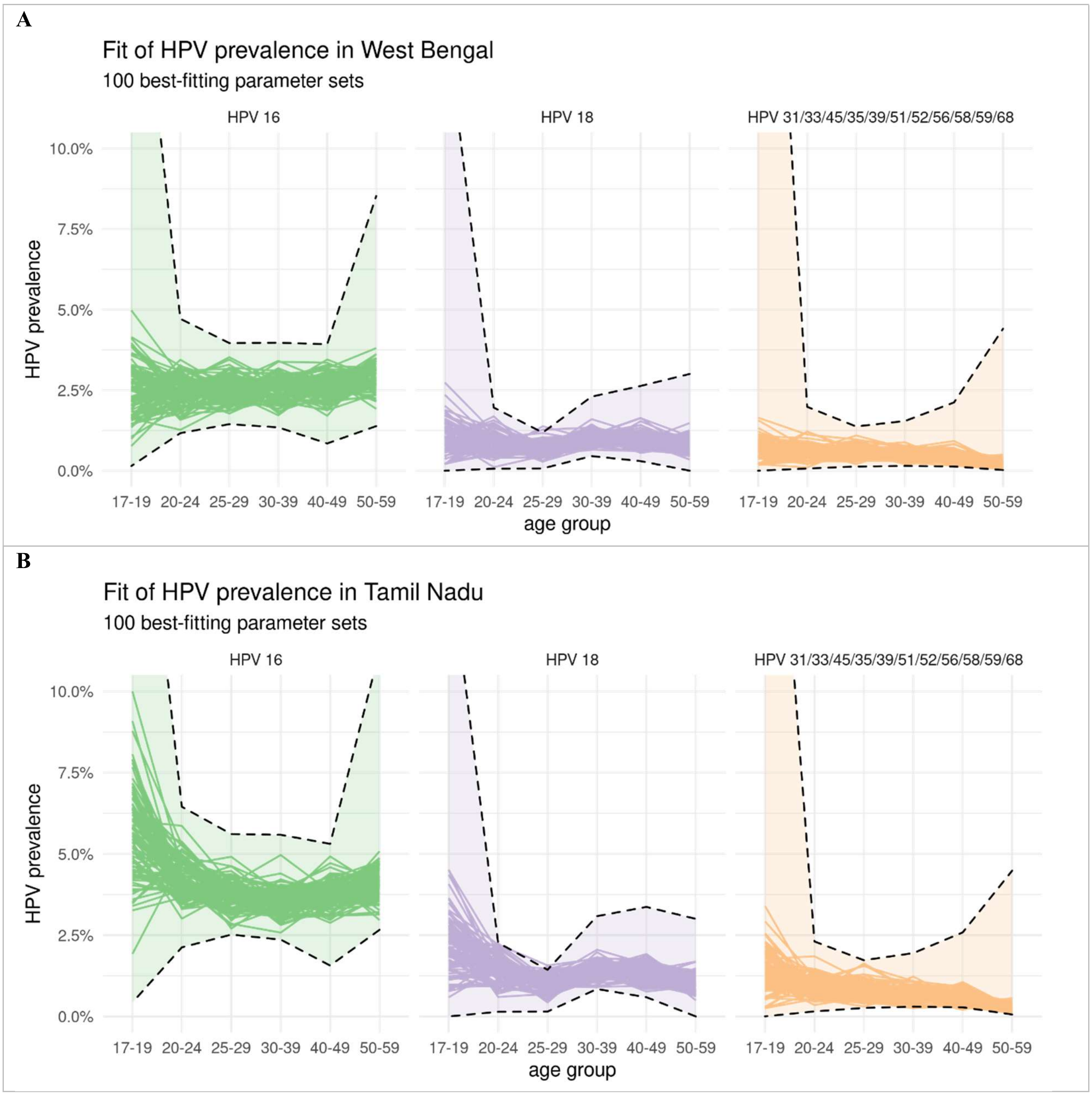

Figure A3. Model fit of the type-specific HPV prevalence data. Panel A: West Bengal. Panel B: Tamil Nadu. Model estimates of HPV prevalence of each of the 100 best-fitting parameter sets are given by a separate line. The confidence intervals of the observed HPV prevalence under binomial distribution are given by the dashed lines. The confidence intervals for West Bengal follow the same shape as those for Tamil Nadu, as the type-specific prevalence for West Bengal was derived by rescaling that of Tamil Nadu (the prevalence for West Bengal being approximately half that of Tamil Nadu).

Table A1. List of model parameters.

Notation	Description	Values / ranges	Reference
Demography			
$m_{g,age}$	Gender- and age-specific mortality	See Table A7	Based UN life tables of India in 2015-2020.(14)
b	Population sex-specific birth rate	Set such that the total population stays constant given $m_{g,age}$ and 50%-50% distribution of female and male new-borns	NA
Sexual contact behaviour			
$p_{W,1}, p_{M,1}$	Probability of being assigned to risk group 1 in women and men	1.3%, 3.3% for West Bengal<br>1.7%, 2.8% for Tamil Nadu	Proportion of virgins in the age group 30-49 estimated by DHS in West Bengal and Tamil Nadu.[12]
$p_{M,3}, p_{M,4}, p_{M,5}$	Probability of being assigned to risk group 3, 4, and 5 in men	3.6%, 4.7%, 1.4% for West Bengal<br>7.9%, 4.6%, 2.2% for Tamil Nadu	Based on Table 1 of Gaffey et al.[13] The rows for low- and high-HIV states were used for West Bengal and Tamil Nadu, respectively. Risk groups 3, 4 and 5 were based on the proportion of sexually active married men having non-regular partners, sexually active unmarried men having non-regular partners but not using female sex workers, and sexually active unmarried men using female sex worker, respectively.
$p_{W,5}$	Probability of being assigned to risk group 5 in women, representing female sex workers	0.5% for West Bengal and Tamil Nadu	Estimated prevalence of female sex workers for India in Table 2 of Vandepitte et al.[14]
$p_{W,3}, p_{W,4}$	Probability of being assigned to risk groups 3 and 4 in women	2.5%, 2.5% for West Bengal<br>2.65%, 2.65% for Tamil Nadu	Urban percentage of women with at least one non-regular partners last year after subtracting the proportion of female sex workers in Table 6.2 of the NACO report.[9]
$p_{W,2}, p_{M,2}$	Probability of being assigned to risk group 2 in women and men	86.0%, 93.2% for West Bengal<br>82.5%, 92.5% for Tamil Nadu	Remaining proportion after fixing the proportions of other risk groups.
$d^{stable}_{W,10-14}, d^{stable}_{M,10-14}$	Population proportion with stable partnerships in women and men aged in the age group 10-14	0%	Set as zero given the low proportion of married individuals in this age groups as estimated by DHS.[12]
$d^{stable}_{W,15-19}, d^{stable}_{W,20-24}, d^{stable}_{W,25-29}, d^{stable}_{W,30-49}, d^{stable}_{W,50-59}, d^{stable}_{W,60-99}$	Population proportion with stable partnerships in women in age groups 15-19, 20-24, 25-29, 30-49, 50-59, and 60-99	38.5%, 77.6%, 91.1%, 87.8%, 83%, 83% for West Bengal<br>12.8%, 58.5%, 84.7%, 86.2%, 64%, 64% for Tamil Nadu	Proportion of women married in DHS in West Bengal and Tamil Nadu.[12]
$d^{stable}_{M,15-19}, d^{stable}_{M,20-24}, d^{stable}_{M,25-29}, d^{stable}_{M,30-49}, d^{stable}_{M,50-59}, d^{stable}_{M,60-99}$	Age-group-specific population proportion with stable partnerships in men in age groups 15-19, 20-24, 25-29, 30-49, 50-59, and 60-99	2.4%, 34.8%, 65.8%, 91.6%, 92.3, 92.3% for West Bengal<br>0.3%, 15.2%, 50.1%, 93.1%, 96.9, 96.9% for Tamil Nadu	Proportion of women married in DHS in West Bengal and Tamil Nadu.[12]
$d^{stable}_{W,1}, d^{stable}_{W,4}, d^{stable}_{W,5}, d^{stable}_{M,1}, d^{stable}_{M,4}, d^{stable}_{M,5}$	Population proportion with stable partnerships in risk groups 1, 4, and 5	0%, by definition.	NA
$d^{stable}_{W,2}, d^{stable}_{W,3}$ $d^{stable}_{M,2}, d^{stable}_{M,3}$	Population proportion with stable partnerships in risk groups 2 and 3	Set to match the age-group-specific population proportion with stable partnerships.:<br>75% for West Bengal	NA

		72% for Tamil Nadu	
d^{stable}	Overall population proportion with stable partnerships	Set to match the age-group-specific population proportion with stable partnerships:<br>68% for West Bengal<br>65% for Tamil Nadu	NA
κ^{stable}	Mean age difference in stable partnerships (men minus women)	7.29 years for West Bengal<br>6.58 years for Tamil Nadu	Estimated from DHS for West Bengal and Tamil Nadu.[12]
ω^{main}	Mean spread of absolute age difference in stable partnerships	3.31 years for West Bengal<br>3.28 years for Tamil Nadu	Estimated from DHS for West Bengal and Tamil Nadu.[12]
s^{stable}	Mean duration of stable partnerships	20 years, by assumption.	NA
$d_{W,1}^{one-off}, d_{W,2}^{one-off}, d_{M,1}^{one-off}, d_{M,2}^{one-off}$	Mean degree of one-off partnerships in risk groups 1 and 2	0%, by definition.	NA
$d_{M,3}^{one-off}, d_{M,4}^{one-off}, d_{M,5}^{one-off}$	Mean degree of one-off partnerships in risk groups 3, 4, and 5 in men	1.65, 1.65, 25.57 for West Bengal<br>3.41, 3.41, 70.58 for Tamil Nadu	Derived on in Table 6.4 of the NACO report.[9]
$d_{W,3}^{one-off}, d_{W,4}^{one-off}$	Mean degree of one-off partnerships in risk groups 3 and 4 in women	2, by assumption.	NA
$d_{W,5}^{one-off}$	Sex- and age-group-specific mean degree of one-off partnerships	Set to match the degree of one-off partnerships in men:<br>89.1 partners per year in West Bengal<br>373.4 partners per year in Tamil Nadu	NA
$d^{one-off}$	Overall mean degree of one-off partnerships	Set to match the risk-group-specific degree with one-off partnerships:<br>0.50 partners per year for West Bengal<br>1.98 partners per year for Tamil Nadu	NA
$s^{one-off}$	Mean duration of one-off partnerships	1 unit of time step, by definition	NA
r^{stable}	Mean number of sex acts per time step within stable partnerships according to a Poisson distribution		
$r^{one-off}$	Fixed number of sex acts per time step within one-off partnerships	1 act, by definition	NA
$\theta^{one-off}$	One-off partnership underreporting rate	Derived from model calibration with the following range for candidate values: (8, 20)	NA
HPV natural history			
β_i	Transmission probability of type i per sex act	Derived from model calibration with the following range for candidate values: (0.2, 0.95).	NA
$\gamma_{16}, \gamma_{18}, \gamma_{cross}, \gamma_{other}$	Clearance rate from CIN0	0.824 per year for HPV 16,<br>0.955 per year for HPV 18,<br>1.18 per year for the cross-protective types and remaining HR HPV types	[4,5]
$\eta_{16}, \eta_{18}, \eta_{cross}, \eta_{other}$	Progression rate from CIN0 to CIN1	0.676 per year for HPV 16,<br>0.545 per year for HPV 18,	[4,5]

		0.324 per year for the cross-protective types and remaining HR HPV types	
$\delta_{16,1}$, $\delta_{18,1}$, $\delta_{cross,1}$, $\delta_{other,1}$	Clearance rate from CIN1	0.133 per year for HPV 16,<br>0.386 per year for HPV 18,<br>0.481 per year for the cross-protective types and remaining HR HPV types	[4,5]
$\delta_{16,2}$, $\delta_{18,2}$, $\delta_{cross,2}$, $\delta_{cross,2}$	Clearance rate from regressive CIN2/3	2.10 per year for HPV 16,<br>2.10 per year for HPV 18,<br>2.10 per year for the cross-protective types and remaining HR HPV types	[4,5]
$\nu_{16,1}$,$\nu_{18,1}$, $\nu_{cross,1}$, $\nu_{other,1}$	Progression rate from CIN1 to regressive CIN2/3	0.048 per year for HPV 16,<br>0.00681 per year for HPV 18,<br>0.0447 per year for the cross-protective types and remaining HR HPV types	[4,5]
$\nu_{16,2}$,$\nu_{18,2}$, $\nu_{cross,2}$, $\nu_{othe\ ,2}$	Progression rate from CIN1 to non-regressive CIN2/3	0.0454 per year for HPV 16,<br>0.0450 per year for HPV 18,<br>0.0110 per year for the cross-protective types and remaining HR HPV types	[4,5]
μ_{16}, μ_{18}, μ_{cross}, μ_{othe}	Rate of waning natural immunity	0.0407 per year for HPV 16,<br>0.0287 per year for HPV 18,<br>0.0320 per year for the cross-protective types and remaining HR HPV types	[4,5]

A.3. Computation of model outcomes

A.3.1. Prevalence of HPV infection

Model estimates on prevalence of four representative HPV types ($X_{16}, X_{18}, X_{cross}, X_{other}$) were reported. In addition, these were used to derive the prevalence of groups of HPV types:

- $X_{16,18}$: HPV 16/18
- $X_{16,18,31,33,45}$: HPV 16/18/31/33/45
- $X_{any\ HR}$: Any HR HPV, i.e., HPV 16/18/31/33/45/35/39/51/52/56/58/59/68

To account for the clustering of HPV types in hosts, the following scaling formulae were used to scale the model prevalence estimates of the four representative HPV types:

- $X_{16,18} = \alpha_{18}X_{18} + \alpha_{16}X_{16}$
- $X_{16,18,31,33,45} = (\beta_{45} + \beta_{33} + \beta_{31})X_{cross} + \beta_{18}X_{18} + \beta_{16}X_{16}$
- $X_{any\ HR} = (\gamma_{68} + \gamma_{59} + \gamma_{58} + \gamma_{56} + \gamma_{52} + \gamma_{51} + \gamma_{39} + \gamma_{35})X_{other} + (\gamma_{45} + \gamma_{33} + \gamma_{31})X_{cross} + \gamma_{18}X_{18} + \gamma_{16}X_{16}$

The three series of scaling parameters ($\alpha_i, \beta_i, \gamma_i$) as given in **Table A4** were obtained from the HPV prevalence survey in Tamil Nadu using the data of co-infection patterns in hosts.[10] For each series given in each column of the table, each parameter, reading from top to bottom, corresponds to the proportion of women infected with that type that were not infected with any of the HPV types in the preceding rows. As an example, for series α_i, 95% of all women infected with HPV 16 were not infected by HPV 18. As another example, for series β_i, 91% of all women infected with HPV 33 were not infected by 45, and 96% of all women infected by HPV 31 were not infected by either 33 or 45. We expect this approach to approximate prevalence of groups of HPV types to work best under vaccination that either reduces the prevalence of any given HPV type substantially. Therefore, the prevalence at the new equilibrium should be well approximated.

As high-quality state-specific HPV prevalence data were not at our disposal, we assumed the model estimates for West Bengal and Tamil Nadu to apply to all states in the low- and high-cancer-incidence clusters, respectively.

A.3.2. Cumulative risk of HPV infection

For each of the four representative HPV type i and each birth cohort, model estimates on half-year incidence $r_{i,a}$ up to age $a = 40$ years were combined to derive the cumulative risk of HPV infection Y_i, as follows: $Y_i = \sum_{a=15}^{45} 0.5 r_{i,a} s_a$. Here, $s_a = \exp\left(-\sum_{a'=10}^{4} 0.5 d_{a'}\right)$ is the survival probability up to age a derived from the mortality rates $d_{a'}$ of the UN life-table of India in 2015-2020 (**Table A5**).

The cumulative risk of any HR HPV infection was a weighted average of the relative reduction in type-specific cumulative risk of HPV infection. The weights were based on the type-specific contribution to cervical cancer as observed in India.[15] The contributions by HPV 16, 18, and 31/33/45 were 62%, 16%, and 6%, respectively (**Table A6**). These proportions were derived by normalizing the type-specific contributions.

As for outcome of HPV prevalence, we assumed the model estimates for cumulative risk of HPV infection for West Bengal and Tamil Nadu to apply to all states in the low- and high-cancer-incidence clusters, respectively.

A.3.3. Life-time risk of cervical cancer

To obtain the life-time risk (LTR) of cervical cancer in the scenario without vaccination, we extracted age-specific cervical cancer incidence data from volume XI of Cancer Incidence in Five Continent (CI5) and the 2012-2016 Report of National Cancer Registry Programme by National Centre for Disease Informatics and Research (NCDIR).[6-8] Of the 25 Indian states, 14 states have local registries and 11 do not. Whenever a registry was present in both CI5 and NCDIR, only the data corresponding to CI5 were included. As described in Section A.2.1., each state without a local cancer registry was classified to either the cluster of states with high or low cervical cancer incidence. The missing incidence were inferred based on the cluster mean of the classified cluster. See **Table A3**, **Figure A4** and **Figure A5** for the extracted or inferred age-specific cervical cancer incidence data by Indian state.

A recently published method [16] was used to derive LTR of cervical cancer from age-specific incidence. This method converts age-specific cancer incidence into LTR of cervical cancer (in cases per 100 000 women born) while accounting for the competing risk of dying from other causes before possible occurrence of cervical cancer. For LTR, we used the UN data on female mortality rates for 2015-2020 in India (**Table A5**).[17] See **Table A9** for the LTR without vaccination by Indian state.

To derive the LTR of cervical cancer in the scenarios with vaccination, we approximated the relative reduction in risk of cervical cancer by model estimates of the relative reduction in the cumulative risk of any HR HPV infection. Model estimates of West Bengal and Tamil Nadu were used for the states in the low- and high-cancer-incidence clusters, respectively.

A.3.4. Standardised life-time risk of cervical cancer

Standardised life-time risk (SLTR) of cervical cancer (in cases per 100,000 women born) was obtained by using the UN data on female mortality rates for 2015-2020 in the ten countries with the highest life-expectancy instead of India (**Table A5**).[17] See **Table A9** for the SLTR without vaccination by state. As for the LTR, the SLTR in the scenarios with vaccination was derived based on model estimates of the relative reduction in the cumulative risk of any HR HPV infection.

A.3.5. Age-standardised incidence rate of cervical cancer

Age-standardised incidence rate (ASIR) of cervical cancer (in cases per 100,000 women-years) was obtained based on the world standard population (**Table A7**).[18] See **Table A9** for the ASIR without vaccination by state. As for the LTR and SLTR, the ASIR in the scenarios with vaccination was derived based on model estimates of the relative reduction in the cumulative risk of any HR HPV infection.

A.3.6. Relative per-dose efficiency

Relative per-dose efficiency is defined as the additional proportion of cervical cancer cases prevented per dose under single- vs two-dose vaccination. In formula, this is given by [Efficiency of single-dose strategy] / [Efficiency of two-dose strategy] - 100%, where [Efficiency of ... strategy] = [% Relative reduction in LTR in routine and CU cohorts combined] / [Doses required in routine and CU cohorts combined].

A.3.7. Aggregating model outcomes

To obtain outcomes for India as a whole and by low- and -high-incidence states, state-specific outcomes were weighted based on the state-specific female population size according to the table C-13 by the Indian Census of 2011 (**Table A8**).[19]

Model outcomes corresponding to the simulation of the 100 best-fitting parameter sets were used to derive the mean and the 10th and 90th percentiles, i.e., Uncertainty Interval (UI), of the model outcomes. For the nationwide model outcomes, the 100 best-fitting parameter sets of West Bengal and Tamil Nadu were paired for derived 100x100 outcomes, which were subsequently used to derive the mean and the 10th and 90th percentiles. Furthermore, nationwide outcomes and outcomes across all high- and low-cancer-incidence states were obtained by weighting the state-specific outcomes by the corresponding population sizes.

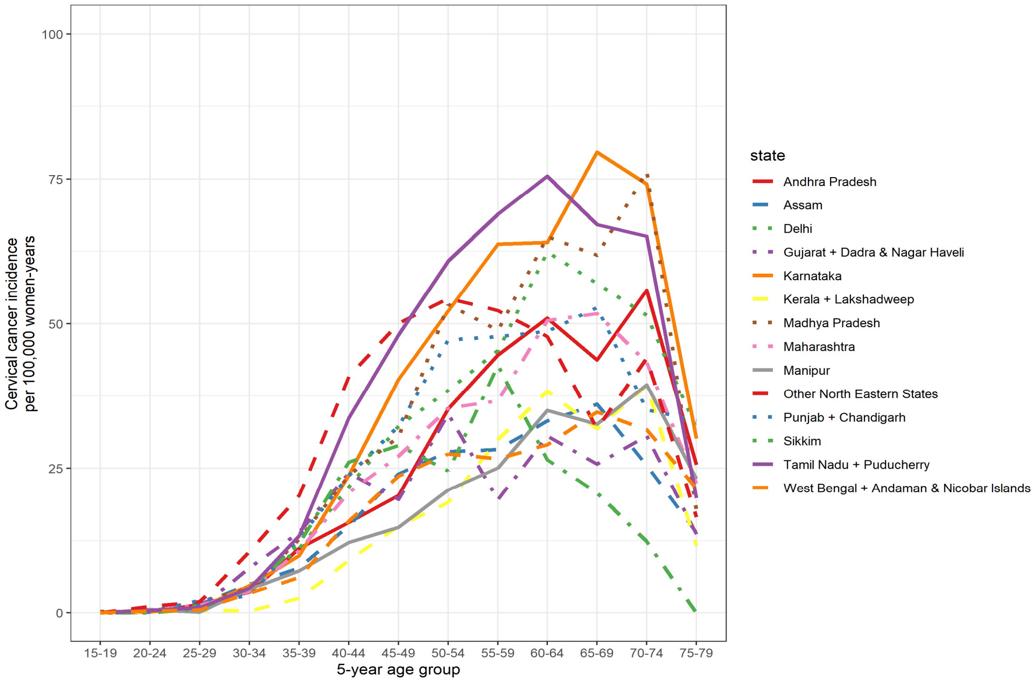

Figure A4. Age-specific cervical cancer incidence data by Indian state.

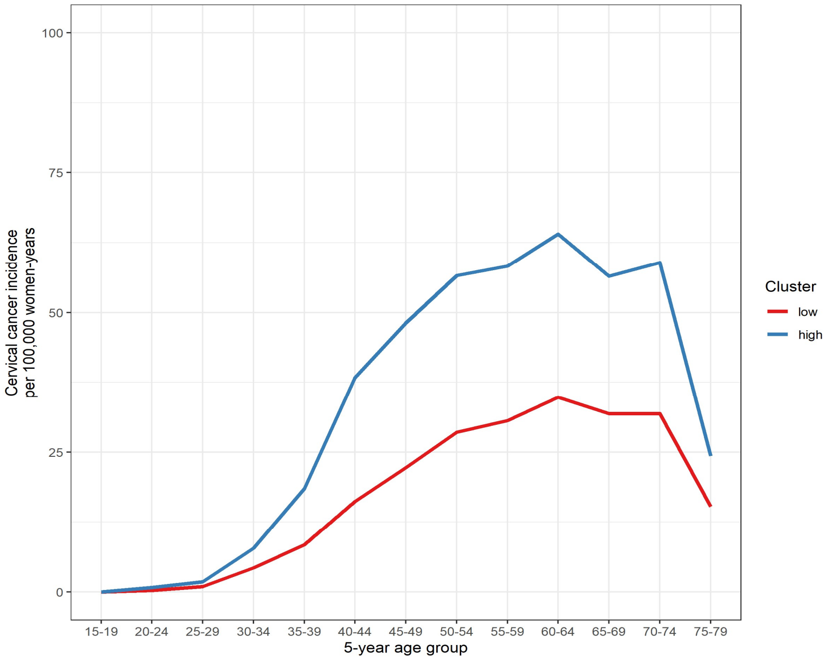

Figure A5. Mean of age-specific cervical cancer incidence of the cluster of Indian states

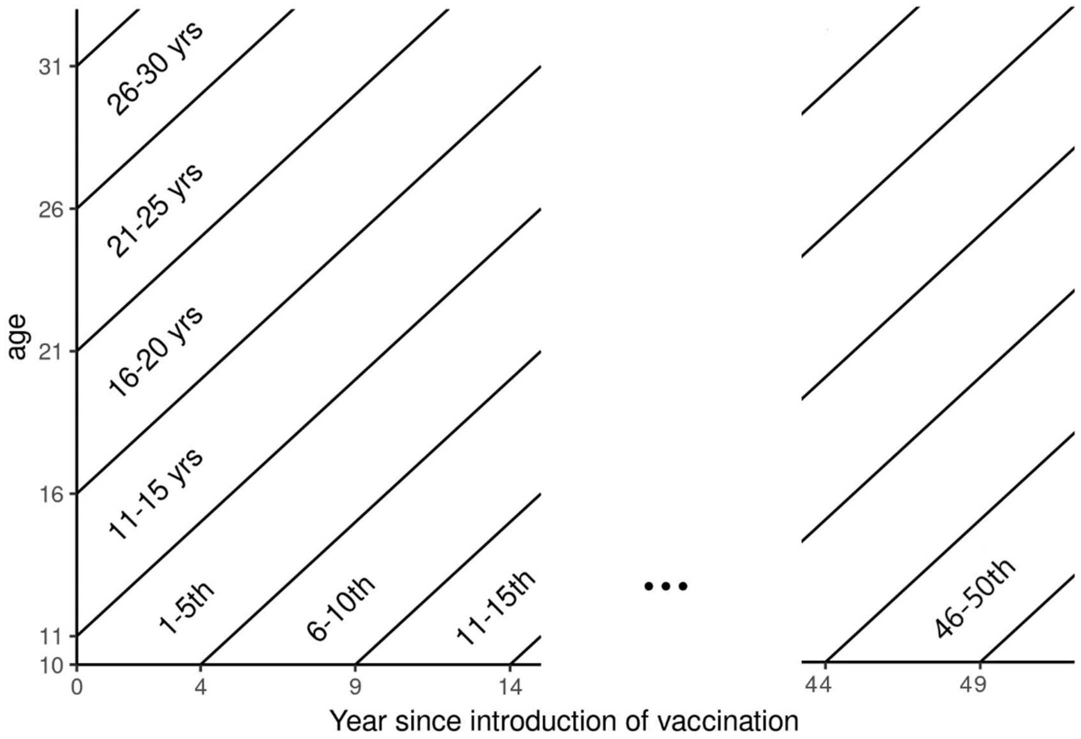

Figure A6. Lexis diagram showing the 5-year birth cohorts targeted by HPV vaccination.
"1-5th" represents the first 5-year vaccinated cohort. This is the birth cohort in which the "short-term" reduction in HPV infection risk and cervical cancer risk are defined. "46-50th" represents the 5-year birth cohort vaccinated at 46 to 50 years after the start of vaccination. This is the birth cohort in which the "long-term" reduction in HPV infection risk and cervical cancer risk are defined.

Table A2. Overview of available cancer incidence data from local registries by Indian state.
* States or groups of states as reported in the 2006 National Behaviour Surveillance Survey of the National AIDS Control Organization of India.[9]
§ Other North Eastern States include Arunachal Pradesh, Nagaland, Meghalaya, Mizoram, and Tripura.
¤ The eighteen registries CI5 and NCDIR do not have in common are in *italics*.

State/group of states *	CI5 registry ¤ [7]	NCDIR registry ¤ [8]
Andhra Pradesh		*Hyderabad district*
Assam	Cachar, Kamrup Urban District	Cachar district, *Dibrugarh district*, Kamrup urban
Bihar		
Chhattisgarh		
Delhi		*Delhi*
Goa + Daman & Diu		
Gujarat + Dadra & Nagar Haveli	Ahmedabad	Ahmedabad urban
Haryana		
Himachal Pradesh		
Jammu & Kashmir		
Jharkhand		
Karnataka	Bangalore	Bangalore
Kerala + Lakshadweep	Kollam, Trivandrum	Kollam district, Thi'puram district
Madhya Pradesh	Bhopal	Bhopal
Maharashtra	Barshi & Paranda & Bhum, Mumbai, Poona, Wardha	*Aurangabad*, *Osamanabad & Beed*, Barshi rural, Mumbai, Pune, Wardha district, *Nagpur*
Manipur		*Manipur state*, *Imphal West district*
Orissa		
Other North Eastern States §	Mizoram, Tripura	Mizoram state, *Aizawl district*, Tripura state, *West Arunachal, Papumpare district, Meghalaya, East Khasi Hills district, Nagaland, Pasighat*
Punjab + Chandigarh		*Patiala district*
Rajasthan		
Sikkim	Sikkim State	Sikkim state
Tamil Nadu + Puducherry	Chennai, *Dindigul Ambilikkai*	Chennai
Uttar Pradesh		
Uttarakhand		
West Bengal + Andaman & Nicobar Islands		*Kolkata*

Table A3. Age-specific cervical cancer incidence data by Indian state.
Incidence is given in cases per 100 000 women-years by 5-year age groups.
* States or groups of states as reported in the 2006 National Behaviour Surveillance Survey of the National AIDS Control Organization of India.[9]
§ Other North Eastern States include Arunachal Pradesh, Nagaland, Meghalaya, Mizoram, and Tripura.
Ŧ "Extracted": cervical cancer incidence data were extracted from CI5 or NCDIR when available; "Inferred": when cervical cancer incidence data were unavailable, they were inferred based on footprinting.[6-8]
¤ Belonging to low- or high-incidence cluster. Cluster was obtained by the clustering step when cervical cancer incidence data were available and by the classification step whenever cervical cancer incidence data were unavailable.

			**Age group**													
**State/group of states ***	**Source Ŧ**	**Cluster ¤**	**15-19**	**20-24**	**25-29**	**30-34**	**35-39**	**40-44**	**45-49**	**50-54**	**55-59**	**60-64**	**65-69**	**70-74**	**75-79**	**80-84**
Andhra Pradesh	Extracted	Low	0	0.1	1.5	3.6	11.2	15.7	20.3	35.3	44.6	51	43.8	55.7	25.7	12.8
Assam	Extracted	Low	0	0.2	1.5	5.1	7.9	15.1	24.1	27.9	28.2	33.2	36.1	25.5	13.9	7
Bihar	Inferred	Low	0	0.3	1	4.3	8.4	16.2	22.2	28.6	30.7	34.9	31.9	31.9	15.3	7.6
Chhattisgarh	Inferred	Low	0	0.3	1	4.3	8.4	16.2	22.2	28.6	30.7	34.9	31.9	31.9	15.3	7.6
Delhi	Extracted	High	0	0.6	1.2	4.4	11.2	21.8	32.3	38.5	45.4	62.3	57	51.4	31.9	16
Goa + Daman & Diu	Inferred	Low	0	0.3	1	4.3	8.4	16.2	22.2	28.6	30.7	34.9	31.9	31.9	15.3	7.6
Gujarat + Dadra & Nagar Haveli	Extracted	Low	0	0	0.8	7.9	14	24.2	19.7	34.4	19.6	30.6	25.7	30.6	13.6	6.8
Haryana	Inferred	Low	0	0.3	1	4.3	8.4	16.2	22.2	28.6	30.7	34.9	31.9	31.9	15.3	7.6
Himachal Pradesh	Inferred	Low	0	0.3	1	4.3	8.4	16.2	22.2	28.6	30.7	34.9	31.9	31.9	15.3	7.6
Jammu & Kashmir	Inferred	Low	0	0.3	1	4.3	8.4	16.2	22.2	28.6	30.7	34.9	31.9	31.9	15.3	7.6
Jharkhand	Inferred	Low	0	0.3	1	4.3	8.4	16.2	22.2	28.6	30.7	34.9	31.9	31.9	15.3	7.6
Karnataka	Extracted	High	0	0.2	0.9	4.9	10	23.8	40.4	52.2	63.7	64	79.6	74.1	30.2	15.1
Kerala + Lakshadweep	Extracted	Low	0	0.2	0.4	0.3	2.5	9.2	15	19.1	30	38.4	31.9	39.3	11.7	5.8
Madhya Pradesh	Extracted	High	0.2	0.2	2	4.3	12.8	23.8	30.4	53.3	48.8	65	61.8	76.4	17.7	8.8
Maharashtra	Extracted	Low	0	0.4	1.3	3.8	10.8	20.8	27.1	35.4	36.8	50.5	51.7	43.3	22.4	11.2
Manipur	Extracted	Low	0	0.6	0.1	4.2	7.4	12.2	14.8	21.3	25.1	35	32.7	39.4	23.2	11.6
Orissa	Inferred	Low	0	0.3	1	4.3	8.4	16.2	22.2	28.6	30.7	34.9	31.9	31.9	15.3	7.6
Other North Eastern States §	Extracted	High	0	1.1	1.9	10.7	20.3	40.9	50	54.3	52.2	47.8	31.9	44	16.6	8.3
Punjab + Chandigarh	Extracted	High	0	0.1	2.2	3	13	23.8	32.3	47.2	47.8	48.7	52.8	35.2	33.3	16.6
Rajasthan	Inferred	Low	0	0.3	1	4.3	8.4	16.2	22.2	28.6	30.7	34.9	31.9	31.9	15.3	7.6
Sikkim	Extracted	Low	0	0	0.7	4.3	12	26.1	29	24.6	42.8	26.5	20.8	12.4	0	0
Tamil Nadu + Puducherry	Extracted	High	0	0.4	0.9	4.3	13.4	33.7	48.1	60.8	68.9	75.5	67.1	65.1	19.9	9.9
Uttar Pradesh	Inferred	Low	0	0.3	1	4.3	8.4	16.2	22.2	28.6	30.7	34.9	31.9	31.9	15.3	7.6
Uttarakhand	Inferred	Low	0	0.3	1	4.3	8.4	16.2	22.2	28.6	30.7	34.9	31.9	31.9	15.3	7.6
West Bengal + Andaman & Nicobar Islands	Extracted	Low	0	0.3	0.6	3.5	6.2	15.9	23.7	27.5	26.7	29.1	34.8	31.7	21.5	10.8

Table A4. Scaling parameters (α_i, β_i, γ_i) for prevalence of groups of HPV types.
Obtained from the HPV prevalence survey in Tamil Nadu using the data of co-infection patterns in hosts.[10]

HPV type i	α_i	β_i	γ_i
68	-	-	1
59	-	-	1
58	-	-	1
56	-	-	1
52	-	-	1
51	-	-	1
39	-	-	0.91
35	-	-	0.89
45	-	1	1
33	-	0.91	0.73
31	-	0.96	0.88
18	1	1	0.84
16	0.95	0.9	0.81

Table A5. Female mortality rate of India and ten countries with the highest life expectancy.
Obtained from UN life tables for 2015-2020.[17]
* Average across the ten countries with the highest life expectancy on the right.

			Ten countries /geographical regions with the highest life expectancy									
**Age group**	**India**	**Average ***	**Hong Kong**	**Japan**	**Macao**	**Switzerland**	**Singapore**	**Italy**	**Spain**	**Australia**	**Channel Is-lands**	**Iceland**
0-0	0.03290	0.00240	0.00122	0.00169	0.00166	0.00320	0.00158	0.00243	0.00221	0.00286	0.00604	0.00108
1-4	0.00218	0.00014	0.00018	0.00017	0.00013	0.00010	0.00009	0.00011	0.00012	0.00013	0.00030	0.00010
5-9	0.00075	0.00006	0.00007	0.00007	0.00004	0.00006	0.00004	0.00006	0.00006	0.00008	0.00011	0.00004
10-14	0.00061	0.00007	0.00007	0.00006	0.00004	0.00007	0.00007	0.00007	0.00007	0.00007	0.00009	0.00013
15-19	0.00100	0.00013	0.00011	0.00013	0.00008	0.00012	0.00012	0.00012	0.00011	0.00020	0.00014	0.00014
20-24	0.00129	0.00018	0.00014	0.00022	0.00016	0.00016	0.00013	0.00015	0.00013	0.00023	0.00021	0.00023
25-29	0.00136	0.00020	0.00017	0.00026	0.00020	0.00020	0.00015	0.00015	0.00016	0.00027	0.00025	0.00018
30-34	0.00154	0.00026	0.00025	0.00034	0.00023	0.00026	0.00024	0.00023	0.00022	0.00038	0.00031	0.00015
35-39	0.00201	0.00040	0.00040	0.00048	0.00034	0.00037	0.00035	0.00036	0.00039	0.00055	0.00042	0.00039
40-44	0.00290	0.00066	0.00065	0.00073	0.00046	0.00062	0.00059	0.00064	0.00068	0.00082	0.00061	0.00084
45-49	0.00400	0.00105	0.00106	0.00113	0.00074	0.00105	0.00108	0.00114	0.00117	0.00127	0.00096	0.00087
50-54	0.00761	0.00166	0.00165	0.00170	0.00115	0.00173	0.00176	0.00184	0.00184	0.00190	0.00148	0.00158
55-59	0.01060	0.00254	0.00240	0.00239	0.00168	0.00281	0.00279	0.00277	0.00265	0.00280	0.00232	0.00276
60-64	0.01770	0.00401	0.00358	0.00345	0.00270	0.00429	0.00440	0.00430	0.00366	0.00429	0.00374	0.00568
65-69	0.02730	0.00645	0.00557	0.00511	0.00489	0.00674	0.00726	0.00668	0.00546	0.00669	0.00709	0.00899
70-74	0.04430	0.01099	0.00927	0.00822	0.00959	0.01072	0.01304	0.01083	0.00935	0.01158	0.01350	0.01379
75-79	0.06650	0.02069	0.01780	0.01506	0.01884	0.01917	0.02456	0.02077	0.01849	0.02044	0.02562	0.02620
80-84	0.10800	0.04101	0.03455	0.03031	0.04047	0.04047	0.04372	0.04306	0.03968	0.04087	0.04858	0.04840
85-89	0.16400	0.08085	0.06229	0.06317	0.07637	0.08752	0.07885	0.08979	0.08342	0.08326	0.08716	0.09663
90-94	0.24200	0.15040	0.10895	0.12643	0.14090	0.17486	0.13722	0.16574	0.16090	0.16180	0.14520	0.18197
95-99	0.22500	0.25650	0.18539	0.22975	0.24119	0.31079	0.22770	0.28747	0.28083	0.28492	0.22719	0.28979

Table A6. Type-specific contribution of HPV types in cervical cancer.
Obtained from a study of HPV distribution in cervical cancer in India.[15]

HPV type	16	18	31	33	35	39	45	52	56	58	59	68	73	other	Contribution of a given combination of HPV types (%)
Combination of HPV types	x														57.5
		x													10.4
			x												1.0
				x											3.1
						x									0.5
							x								1.6
								x							1.0
									x						1.6
										x					1.6
											x				2.1
	x	x													7.3
	x		x												0.5
	x			x											1.0
	x				x										1.0
	x					x									0.5
	x							x							1.0
	x								x						0.5
	x									x					0.5
	x											x			0.5
													x		0.5
		x			x										0.5
														x	5.8
**Unnormalized contributions (%)**	70	18	2	4	2	1	2	2	2	2	2	1	1	6	
**Normalized contributions (%)**	62	16	1	4	1	1	1	2	2	2	2	0	0	5	

Table A7. Standard world population.[18]

Age group	Population
0-4	12000
5-9	10000
10-14	9000
15-19	9000
20-24	8000
25-29	8000
30-34	6000
35-39	6000
40-44	6000
45-49	6000
50-54	5000
55-59	4000
60-64	4000
65-69	3000
70-74	2000
75-79	1000
80-84	500
85+	500
Total	100000

Table A8. Female population size by Indian state.
Extracted from table C-13 by the Indian Census.[19]
* States or groups of states as reported in the 2006 National Behaviour Surveillance Survey of the National AIDS Control Organization of India.[9]
ǂ Clustering of states into groups of high and low cervical cancer incidence was derived in a separate manuscript.[6]
§ Other North Eastern States include Arunachal Pradesh, Nagaland, Meghalaya, Mizoram, and Tripura.

State/group of states *	Cluster ǂ	Population size	Percentage (%)
Andhra Pradesh	Low	41754886	7.13
Assam	Low	15257203	2.61
Bihar	Low	49638102	8.48
Chhattisgarh	Low	12701295	2.17
Delhi	High	7793088	1.33
Goa + Daman & Diu	Low	811247	0.14
Gujarat + Dadra & Nagar Haveli	Low	28984911	4.95
Haryana	Low	11842082	2.02
Himachal Pradesh	Low	3377919	0.58
Jammu & Kashmir	Low	5895268	1.01
Jharkhand	Low	16003337	2.73
Karnataka	High	30108199	5.14
Kerala + Lakshadweep	Low	17392769	2.97
Madhya Pradesh	High	34975017	5.97
Maharashtra	Low	53942893	9.21
Manipur	Low	1413663	0.24
Orissa	Low	20704258	3.54
Other North Eastern States §	High	5435403	0.93
Punjab + Chandigarh	High	13559265	2.32
Rajasthan	Low	32865353	5.61
Sikkim	Low	286968	0.05
Tamil Nadu + Puducherry	High	36611821	6.25
Uttar Pradesh	Low	94575702	16.15
Uttarakhand	Low	4941223	0.84
West Bengal + Andaman & Nicobar Islands	Low	44595926	7.62
Total (Low-incidence cluster)		456985005	78.05
Total (High-incidence cluster)		128482793	21.94
Total (all states)		585467798	100.00

Table A9. Pre-vaccination risk of cervical cancer by Indian state.
* States or groups of states as reported in the 2006 National Behaviour Surveillance Survey of the National AIDS Control Organization of India.[9]
§ Other North Eastern States include Arunachal Pradesh, Nagaland, Meghalaya, Mizoram, and Tripura.
ƚ Cases per 100 000 women born
ǂ Cases per 100 000 women

State/group of states *	Life-time risk ƚ	Standardised life-time risk ƚ	Age-standardised incidence rate ǂ
Andhra Pradesh	1157	1480	11.5
Assam	849	1054	8.9
Bihar	870	1087	9
Chhattisgarh	870	1087	9
Delhi	1353	1722	13.7
Goa + Daman & Diu	870	1087	9
Gujarat + Dadra & Nagar Haveli	868	1067	9.3
Haryana	870	1087	9
Himachal Pradesh	870	1087	9
Jammu & Kashmir	870	1087	9
Jharkhand	870	1087	9
Karnataka	1667	2116	16.8
Kerala + Lakshadweep	738	944	7.3
Madhya Pradesh	1498	1882	15.3
Maharashtra	1158	1462	11.9
Manipur	804	1044	7.9
Orissa	870	1087	9
Other North Eastern States §	1483	1788	16.3
Punjab + Chandigarh	1304	1643	13.4
Rajasthan	870	1087	9
Sikkim	820	956	9.2
Tamil Nadu + Puducherry	1764	2180	18.5
Uttar Pradesh	870	1087	9
Uttarakhand	870	1087	9
West Bengal + Andaman & Nicobar Islands	843	1072	8.6
Total (Low-incidence cluster)	922	1158	9.5
Total (High-incidence cluster)	1583	1983	16.3
Total (all states)	1067	1339	11

A.4. Vaccination scenarios

A.4.1. Overview of vaccination scenarios

Table A10. Overview of vaccination scenarios.
° See Section A.4.3. for the construction of the vaccine protection assumptions.

Name	Vaccine protection assumptions for single-dose vaccination °	Routine coverage	Max. age catch-up	Catch-up coverage	Number of scenarios
A90 (base-case)	A	90%	NA	NA	1
A60, A70, A80, A100	A	60, 70, 80, 100%	NA	NA	4
B60, B70, B80, B90, B100	B	60, 70, 80, 90, 100%	NA	NA	5
C60, C70, C80, C90, C100	C	60, 70, 80, 90, 100%	NA	NA	5
D60, D70, D80, D90, D100	D	60, 70, 80, 90, 100%	NA	NA	5
E60, E70, E80, E90, E100	E	60, 70, 80, 90, 100%	NA	NA	5
A90.CU15, A90.CU15+	A	90%	15 years	60, 90%	2
A90.CU20, A90.CU20+	A	90%	20 years	60, 90%	2
A90.CU25, A90.CU25+	A	90%	25 years	60, 90%	2
A90.CU30, A90.CU30+	A	90%	30 years	60, 90%	2
B90.CU20, B90.CU20+	B	90%	20 years	60, 90%	2
C90.CU20, C90.CU20+	C	90%	20 years	60, 90%	2
D90.CU20, D90.CU20+	D	90%	20 years	60, 90%	2
E90.CU20, E90.CU20+	E	90%	20 years	60, 90%	2

A.4.2. Vaccine protection assumptions

In the model, vaccine-induced protection is assumed to impede acquisition of new HPV infections. The vaccine-induced protection is assumed to follow an all-or-nothing mechanism, meaning that a vaccinated individual is either fully protected or not protected at all. Vaccine efficacy is here defined as the proportion of the vaccinated individuals that are fully protected. Waning of vaccine protection is modelled as a decrease in vaccine efficacy, meaning a decrease of the proportion of individuals that are fully protected turning into not protected at all.

In total, we considered four main assumptions of vaccine protection for single-dose vaccination, A-D. Vaccine efficacy was assumed to decrease according to an exponential decay that may plateau above 0%. The following parametric form was used for the decrease of vaccine efficacy: $(VE_{initial} - VE_{plateau}) * e^{-rate*time} + VE_{plateau}$, with $time$ in years. See **Table A11** for the parameter values of $VE_{initial}$, $VE_{plateau}$, and $rate$ corresponding to the different assumptions. The exponential decay functions corresponding to assumptions A, B, C, and D are given in cyan, blue, purple, and pink in **Figure A7**, respectively.

Table A11. Overview of parameters under different vaccine protection assumptions.

	HPV 16			HPV 18			HPV 31/33/45		
**Assumption**	$VE_{initial}$	$VE_{plateau}$	$rate$	$VE_{initial}$	$VE_{plateau}$	$rate$	$VE_{initial}$	$VE_{plateau}$	$rate$
A	0.95	0.95	NA	0.95	0.95	NA	0.09	0.09	NA
B	0.95	0.60	0.02	0.95	0.45	0.02	0.09	0.45/0.95*0.09	0.02
C	0.90	0.55	0.02	0.85	0.35	0.04	0.09	0.35/0.85*0.09	0.04
D	0.85	0.50	0.02	0.55	0.25	0.08	0.09	0.25/0.55*0.09	0.08
E	0.85	0.50	0.02	0.55	0.25	0.08	0	0	NA

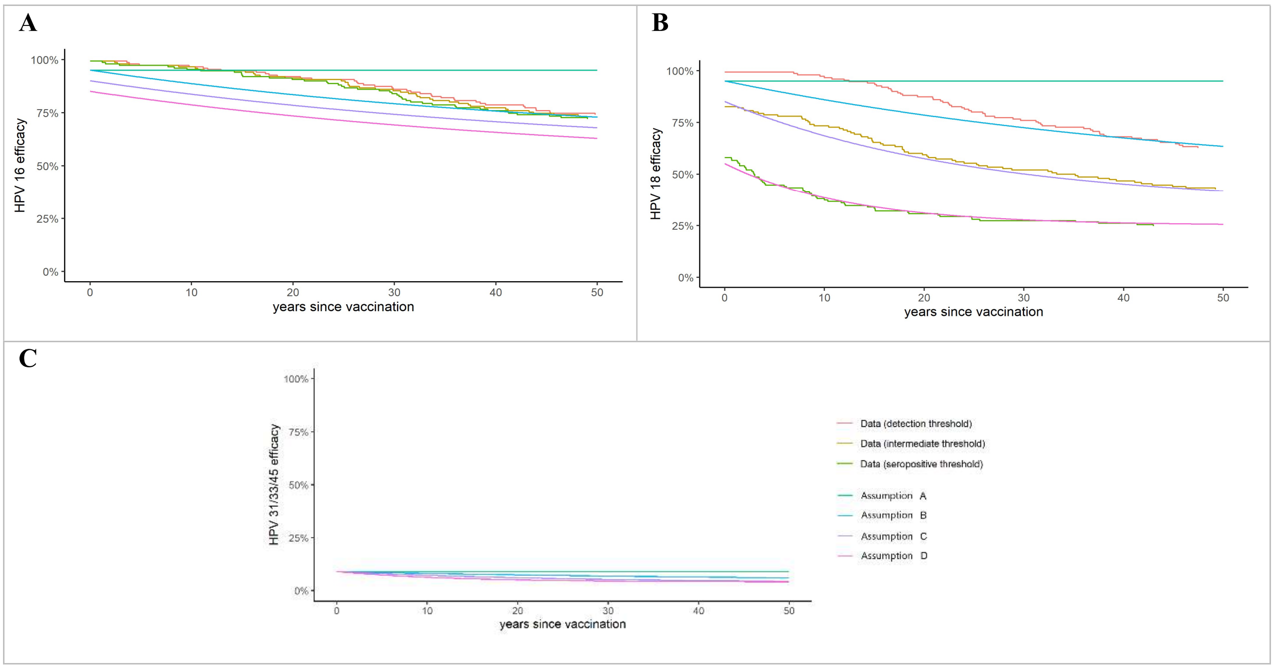

Figure A7. Vaccine efficacy by HPV type for different vaccine protection assumptions for single-dose vaccination. Panel A: HPV 16. Panel B: HPV 18. Panel C: HPV 31/33/45. Cyan: assumption A. Blue: assumption B. Purple: assumption C. Pink: assumption D. Red: survival curve of time until antibody level drops below detection threshold. Green: survival curve of time until antibody level drops below seropositivity threshold. Gold: survival curve of time until antibody level drops below the geometric mean of the detection and seropositivity thresholds.

The assumptions were constructed as follows. Assumption A (cyan in **Figure A7**), which assumed life-long protection for the vaccine targeted types, was based on near-zero number of break-through persistent infections up to 10 years post-vaccination as observed in the IARC India vaccine trial.[20] The efficacy was assumed to be 95% for HPV 16/18, 9% for HPV 31/33/45, and 0% for the remaining HR HPV types, according to the efficacy estimated against persistent infection at 10 years post-vaccination.

Assumptions B, C and D were obtained by projecting the time until the antibody levels of HPV 16/18 decrease below various thresholds using immunogenicity data from the IARC India vaccine trial.[20] See **Figure A8** for the antibody levels at 3 and 10 years since vaccination in the trial's participants. Three thresholds were considered: 1) Detection threshold of 1 international unit (IU)/ML for HPV 16 and 0.3 IU/ML for HPV 18 (red in **Figure A8**);[21] 2) Seropositivity

threshold of 1.3 IU/ML for HPV 16 and 2.9 IU/ML for HPV 18 (green in **Figure A8**); 3) Geometric mean of the detection and seropositivity thresholds of 1.14 IU/ML for HPV 16 and 0.93 IU/ML for HPV 18 (gold in **Figure A8**). Survival curves were obtained for the projected time antibody level drops below the three suggested thresholds (see survival curves of the corresponding colour in **Figure A7**). Exponential decay functions fitting the survival curves were derived for HPV 18 for assumptions B, C and D (**Figure A7**). For HPV 16, there was little variation in the different survival curves. Hence, only the exponential decay function of assumption B was derived from them. The parameter $VE_{initial}$ exponential decay functions for assumptions C and D were based on the estimate lower bound of efficacy of 85% against persistent HPV 16/18 infection at 10 years post-vaccination. Averaging the efficacy of HPV 16 and 18, the remaining efficacy in assumptions B, C, and D are approximately 80%, 75% and 65% for HPV 16/18 after 20 years, respectively, as indicated in the main text. Efficacy for HPV 31/33/45 were derived from efficacy of HPV 18. Same proportional decrease was assumed to derive $VE_{plateau}$, and the same exponential decay rate was assumed (**Table A11**). Finally, assumption E was derived from assumption D with no cross-protection for HPV 31/33/45 (**Table A11**).

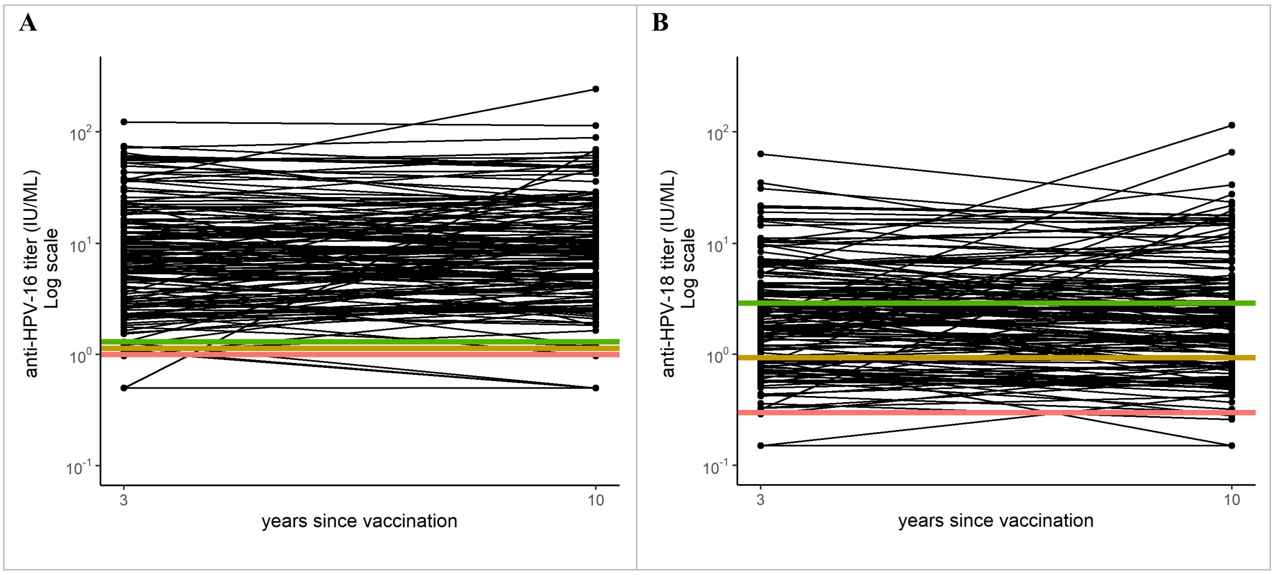

Figure A8. Immunogenicity data of IARC India vaccine trial and thresholds of protection.
Panel A: HPV 16. Panel B: HPV 18. Red: detection threshold. Green: seropositivity threshold. Gold: geometric mean of the detection and seropositivity thresholds. Data points at year 3 and 10 of each trial participant are connected by a straight line.

A.5. HPV-FRAME checklist

Table A12. HPV-FRAME checklist [22]
Y=yes; N=no; F=female; M=male; NA=not applicable

Core reporting standard

**a) Inputs**	**Reported by age? (Y/N)**	**Report by sex? (F-only, M-only or both)**	**Comments**
Target population for intervention	Y	F-only	Only vaccination in girls and women were considered. Age of routine and catch-up vaccination were reported.
Sexual behaviour	Y	Both	Parameters reports: proportion of risk-group; age- and risk-group specific numbers of stable and one-off partnerships; rates of partnership dissolution; number of sex acts per unit of time given established partnership; probability of HPV transmission per sex act. Parameters were reported separately by model for the high and low cervical cancer incidence cluster. Derivation of assortative parameters was reported.
Cohort examined for evaluation / time horizon	N	F-only	Cumulative risk of HPV infection and life-time risk of cervical cancer were presented for the cohorts vaccinated at the 1-5th and 46-50th year in the routine programme, and for the catch-up vaccinated cohort. HPV prevalence and age-standardized incidence rate of cervical cancer were reported up to 50 years after the introduction of vaccination.
Quality of life assumptions	NA	NA	NA
Calibration	Y	Y	The standard STERGM calibration function was used to obtain age- and risk-groups parameters of partnership formation and dissolution. Subsequently, maximum likelihood was used to assess the goodness-of-fit to age-specific HPV prevalence data and to obtain HPV transmission probabilities and one-off partnership underreporting rate.
Validation (where possible)	Y	Y	Validation adequate cervical cancer incidence resulting from the HPV infection incidence here was reported in an accompanying paper.
Costs	NA	NA	NA

Reporting standard for HPV vaccination in adolescent individuals

**a) Inputs**	**Reported? (Y/N)**	**Reported by age? (Y/N)**	**Report by sex? (F-only, M-only or both)**	**Report as calibration or validation target? (Y/N)**
Vaccine uptake	Y	Y	Y	Single- and two-dose schedules were considered. Only vaccination in girls and women were considered. Uptake between 60-100% were considered for routine vaccination. Uptake of 60% and 90% were considered for catch-up vaccination.
Vaccine efficacy	Y	Y	NA	Efficacy by dose schedule and HPV type was considered. Efficacy was independent of age.
Vaccine cross-protection	Y	Y	NA	Level of cross-protection for HPV 31/33/45 was reported.
Duration vaccine protection and waning	Y	Y	NA	Waning assumption by dose schedule and HPV type was considered.
Vaccine and delivery costs	NA	NA	NA	Economic assessment was done in an accompanying paper.
Pre-vaccination disease burden (including population attributable fractions for HPV)	Y, for cervical cancer.	Y, for cervical cancer.	F-only, for cervical cancer.	Attributable fraction by HPV type to cervical cancer burden was reported.
Duration of natural immunity	Y	NA	Y	Natural immunity was independent of age. Sex-specific natural immunity assumptions were reported.

(Table continues the next page)

b) Outputs	Reported? (Y/N)	Reported by age? (Y/N)	Report by sex? (F-only, M-only or both)	Comments
Absolute reductions in HPV infections, and/or warts, post-vaccination	Y, for HPV infections.	N, for HPV infection.	F-only, for HPV infection.	Type-specific HPV-prevalence only evaluated for women aged 15-40 years. Impact on warts was not considered.
Absolute reductions in CIN2+ post-vaccination	N	N	NA	NA
Absolute reductions in invasive cancer (cervical and other HPV cancers, as relevant)	Y, for cervical cancer.	N	F-only, cervical cancer.	NA

Reporting standards for evaluations of vaccination at older ages

a) Inputs	Detail	Reported by age? (Y/N)	Report by sex? (F-only, M-only or both)	Comments
Natural history	Nature history structure used in the model (including progression and regression from high grade to low grade disease/productive HPV infection).	Y	Y	Natural history structure was independent of age. Sex-specific natural history parameters were reported.
Natural history	Rate of clearance of HPV infection	Y	Y	Rate of clearance of HPV infection was independent of age. Sex-specific natural history parameters were reported.
Natural history	Rate of loss of naturally acquired immunity	Y	Y	Rate of loss of naturally acquired immunity was independent of age. Naturally acquired immunity was only assumed for women.
Natural history	Simulation of latency by HPV type (handling of apparently new infections in older women – is the possibility that some are reactivated latent infections explored in sensitivity analysis?)	N	N	No latency assumed in the model.
Vaccination	Vaccine coverage at older ages	Y	NA	Catch-up at older ages was only considered for women.
Vaccination	Whether screen-and-vaccinate is being modelled, or just vaccination at older ages (without linking to screening/HPV status)	NA	NA	We reported that no screening was assumed.

Reporting standard for models of HPV prevention in LMIC

a) Inputs	Reported? (Y/N)	Reported by age? (Y/N)	Report by sex? (F-only, M-only or both)	Comments
HIV prevalence rates if endemic in country	N	N	N	The effects of HIV are not modelled because of the low HIV prevalence in India.[3]
Description of any opportunistic or pilot/demonstration screening project ongoing	Y	NA	NA	Opportunistic cervical cancer screening is done in the Indian national communicable disease control programme. However, the coverage is very low. Ever-in-lifetime coverage was reported to be lower than 3% for women aged 30–49 years in the 2017–18 NCD monitoring survey.[23] Hence, no screening was assumed in the present study.

Reporting standards for evaluations assessing alternative vaccine types or reduced-dose schedules

a) Inputs	Reported? (Y/N)	Reported by age? (Y/N)	Report by sex? (F-only, M-only or both)	Comments
Vaccine efficacy/waning	See Comments.	See Comments.	See Comments.	See entries "Vaccine efficacy" and "Duration vaccine protection and waning" under "Reporting standard for HPV vaccination in adolescent individuals".
Timing between doses (for 2-dose)	NA	NA	NA	Timing between the two doses under two-dose vaccination schedule was not modelled. We assumed constant and life-long efficacy under two-dose schedule.

(Table continues the next page)

Vaccine cross-protection	See Comments.	See Comments.	See Comments.	See entries "Vaccine cross-protection" under "Reporting standard for HPV vaccination in adolescent individuals".
Cost	NA	NA	NA	NA
**b) Outputs**	**Reported? (Y/N)**	**Reported by age? (Y/N)**	**Report by sex? (F-only, M-only or both)**	**Report as calibration or validation target (Y/N)?**
Threshold cost per dose	NA	NA	NA	NA

Appendix B

B.1. Supplementary results in tables

Table B1. Relative reduction in HR HPV prevalence over time in the base-case scenario

§ See Table A3 for which Indian states are assigned to high- or low-cancer-incidence clusters.

¤ Any HR HPV includes HPV 16/18/31/33/45/35/39/51/52/56/58/59/68.

ǂ With prevalence of 2.6% for HPV 16 and 1.2% for HPV 18 at the start of vaccination, the prevalence becomes 26 per 100.000 or lower in 75 years since the start of vaccination, which in the order of magnitude that has been suggest to allow stochastic extinction.[24]

Groups of Indian states §	Year since start of vaccination	HPV 16	HPV 18	HPV 16/18	HPV 31/33/45	HPV 16/18/31/33/45	any HR HPV¤
India (all states)	10	20% (9, 30)	27% (9, 45)	22% (13, 31)	20% (-35, 61)	22% (2, 36)	11% (-10, 32)
India (all states)	20	60% (53, 67)	69% (60, 79)	63% (57, 69)	22% (-32, 63)	49% (31, 63)	25% (1, 48)
India (all states)	50	96% (94, 98)	99% (97, 100)	97% (96, 99)	31% (-10, 69)	75% (60, 87)	38% (16, 61)
India (all states)	75	99% (99, 100) ǂ	100% (100, 100) ǂ	99% (99, 100)	30% (-23, 70)	76% (57, 89)	39% (16, 62)
Low incidence	10	20% (7, 33)	28% (1, 50)	22% (10, 34)	22% (-60, 75)	22% (-4, 40)	12% (-16, 41)
Low incidence	20	59% (52, 69)	69% (57, 81)	62% (55, 71)	22% (-45, 74)	49% (23, 66)	25% (-8, 57)
Low incidence	50	97% (94, 99)	99% (97, 100)	97% (95, 99)	35% (-19, 79)	76% (58, 91)	39% (10, 68)
Low incidence	75	99% (98, 100)	100% (100, 100)	100% (99, 100)	33% (-38, 82)	77% (52, 94)	40% (12, 71)
High incidence	10	20% (6, 33)	25% (3, 46)	21% (11, 32)	17% (-32, 65)	20% (2, 39)	10% (-12, 30)
High incidence	20	61% (53, 68)	70% (60, 80)	64% (58, 69)	21% (-40, 65)	49% (27, 65)	25% (2, 49)
High incidence	50	96% (93, 98)	99% (97, 100)	97% (95, 98)	23% (-34, 66)	71% (52, 86)	36% (12, 61)
High incidence	75	99% (98, 100)	100% (100, 100)	99% (98, 100)	24% (-23, 62)	73% (57, 86)	37% (12, 62)

Table B2. Relative reduction in nationwide HR HPV prevalence over time across single-dose protection assumptions
¤ Any HR HPV includes HPV 16/18/31/33/45/35/39/51/52/56/58/59/68.

Single-dose protection scenario	Routine coverage	Year since start of vaccination	HPV 16	HPV 18	HPV 16/18	HPV 31/33/45	HPV 16/18/31/33/45	any HR HPV¤
A	0.6	10	13% (1, 24)	20% (-2, 40)	15% (5, 25)	20% (-35, 61)	17% (-3, 32)	9% (-12, 30)
A	0.6	20	42% (34, 51)	52% (40, 66)	45% (38, 53)	20% (-39, 63)	37% (16, 52)	19% (-6, 43)
A	0.6	50	72% (66, 78)	83% (72, 91)	75% (70, 80)	24% (-27, 64)	58% (40, 73)	30% (4, 53)
A	0.6	75	76% (70, 82)	85% (73, 94)	79% (73, 83)	20% (-34, 67)	59% (40, 75)	30% (7, 54)
A	0.7	10	15% (4, 26)	22% (2, 42)	18% (8, 27)	21% (-33, 61)	19% (-0, 33)	10% (-11, 31)
A	0.7	20	48% (40, 56)	58% (45, 70)	51% (44, 58)	23% (-30, 65)	41% (23, 56)	21% (-4, 45)
A	0.7	50	82% (77, 86)	92% (87, 97)	85% (81, 88)	28% (-22, 67)	65% (48, 79)	34% (12, 56)
A	0.7	75	88% (83, 92)	95% (89, 100)	90% (86, 93)	22% (-31, 66)	67% (48, 82)	34% (9, 57)
A	0.8	10	18% (7, 28)	24% (4, 43)	20% (10, 29)	21% (-33, 62)	20% (1, 35)	10% (-10, 32)
A	0.8	20	54% (47, 62)	63% (53, 74)	57% (50, 64)	22% (-30, 64)	45% (27, 60)	23% (-1, 46)
A	0.8	50	90% (87, 93)	97% (94, 99)	92% (90, 95)	28% (-15, 66)	70% (54, 83)	36% (14, 58)
A	0.8	75	96% (93, 98)	99% (98, 100)	97% (95, 98)	25% (-27, 65)	72% (54, 86)	37% (16, 57)
A	0.9	10	20% (9, 30)	27% (9, 45)	22% (13, 31)	20% (-35, 61)	22% (2, 36)	11% (-10, 32)
A	0.9	20	60% (53, 67)	69% (60, 79)	63% (57, 69)	22% (-32, 63)	49% (31, 63)	25% (1, 48)
A	0.9	50	96% (94, 98)	99% (97, 100)	97% (96, 99)	31% (-10, 69)	75% (60, 87)	38% (16, 61)
A	0.9	75	99% (99, 100)	100% (100, 100)	99% (99, 100)	30% (-23, 70)	76% (57, 89)	39% (16, 62)
A	1	10	22% (11, 32)	30% (11, 47)	24% (15, 33)	23% (-31, 62)	24% (5, 38)	12% (-9, 34)
A	1	20	65% (59, 71)	74% (65, 82)	67% (62, 73)	25% (-29, 64)	53% (34, 66)	27% (2, 51)
A	1	50	99% (99, 100)	100% (100, 100)	100% (99, 100)	30% (-16, 67)	75% (59, 88)	39% (16, 61)
A	1	75	100% (100, 100)	100% (100, 100)	100% (100, 100)	26% (-27, 66)	74% (56, 88)	38% (17, 60)
B	0.6	10	12% (0, 23)	18% (-4, 39)	14% (3, 24)	21% (-36, 62)	16% (-4, 32)	9% (-12, 30)
B	0.6	20	39% (30, 48)	45% (29, 62)	41% (32, 50)	20% (-33, 62)	34% (14, 49)	17% (-7, 42)
B	0.6	50	63% (57, 70)	68% (54, 79)	64% (59, 70)	23% (-42, 64)	50% (26, 65)	26% (-0, 50)
B	0.6	75	66% (59, 73)	66% (50, 79)	66% (58, 73)	18% (-36, 62)	49% (29, 65)	25% (2, 48)
B	0.7	10	14% (2, 25)	21% (-0, 40)	16% (6, 26)	20% (-33, 61)	18% (-2, 33)	9% (-12, 31)
B	0.7	20	43% (35, 52)	53% (38, 68)	46% (38, 54)	20% (-39, 62)	37% (18, 52)	19% (-5, 43)
B	0.7	50	73% (68, 79)	76% (64, 86)	74% (69, 79)	23% (-20, 62)	56% (40, 70)	29% (6, 51)
B	0.7	75	76% (71, 82)	75% (60, 87)	76% (70, 82)	22% (-22, 66)	57% (41, 74)	29% (7, 53)
B	0.8	10	17% (5, 27)	23% (2, 42)	19% (8, 28)	20% (-38, 61)	19% (-1, 34)	10% (-11, 31)
B	0.8	20	49% (42, 57)	57% (45, 70)	52% (45, 60)	18% (-33, 61)	40% (23, 55)	21% (-3, 44)
B	0.8	50	82% (77, 86)	85% (76, 93)	83% (78, 87)	27% (-26, 67)	63% (45, 78)	33% (10, 56)
B	0.8	75	86% (82, 90)	86% (77, 94)	86% (82, 90)	19% (-34, 63)	63% (44, 78)	33% (9, 54)
B	0.9	10	18% (8, 29)	25% (6, 44)	20% (11, 29)	22% (-34, 62)	21% (1, 36)	11% (-10, 32)
B	0.9	20	55% (48, 62)	62% (49, 74)	57% (50, 64)	22% (-32, 62)	45% (28, 59)	23% (-1, 46)
B	0.9	50	90% (86, 93)	92% (86, 97)	91% (87, 93)	28% (-20, 65)	69% (52, 82)	35% (12, 57)
B	0.9	75	94% (91, 97)	96% (91, 100)	95% (92, 97)	28% (-20, 66)	72% (55, 85)	37% (14, 60)
B	1	10	21% (11, 31)	28% (9, 46)	23% (14, 31)	20% (-33, 61)	22% (3, 36)	11% (-9, 33)

B	1	20	59% (53, 66)	67% (56, 77)	62% (56, 68)	23% (-31, 63)	48% (30, 62)	25% (-1, 49)
B	1	50	95% (93, 97)	97% (94, 99)	96% (94, 97)	25% (-25, 64)	71% (54, 85)	36% (14, 58)
B	1	75	98% (97, 100)	99% (97, 100)	99% (97, 100)	23% (-43, 65)	72% (49, 87)	37% (13, 60)
C	0.6	10	12% (-1, 23)	16% (-6, 38)	13% (2, 23)	19% (-38, 61)	15% (-5, 30)	8% (-13, 30)
C	0.6	20	37% (28, 46)	37% (20, 55)	37% (29, 46)	19% (-36, 63)	31% (10, 47)	16% (-9, 40)
C	0.6	50	60% (53, 66)	48% (33, 63)	56% (49, 63)	21% (-32, 59)	44% (26, 57)	23% (-0, 45)
C	0.6	75	63% (55, 71)	50% (34, 64)	59% (52, 67)	20% (-30, 65)	45% (27, 62)	23% (2, 47)
C	0.7	10	13% (1, 24)	18% (-3, 38)	15% (4, 24)	19% (-38, 61)	16% (-3, 32)	8% (-12, 30)
C	0.7	20	41% (32, 50)	43% (25, 60)	41% (33, 51)	18% (-40, 61)	33% (12, 49)	17% (-9, 41)
C	0.7	50	70% (64, 76)	57% (41, 71)	66% (59, 72)	18% (-45, 61)	49% (26, 65)	25% (-0, 49)
C	0.7	75	74% (67, 79)	59% (44, 72)	69% (63, 75)	17% (-40, 61)	51% (30, 67)	26% (3, 50)
C	0.8	10	16% (4, 26)	19% (-1, 39)	17% (7, 26)	20% (-36, 62)	18% (-2, 33)	9% (-12, 31)
C	0.8	20	47% (39, 55)	47% (31, 62)	47% (39, 55)	19% (-32, 60)	37% (20, 53)	19% (-4, 42)
C	0.8	50	78% (73, 83)	65% (51, 78)	74% (68, 80)	20% (-34, 64)	55% (35, 71)	28% (5, 50)
C	0.8	75	83% (78, 87)	66% (51, 78)	77% (71, 83)	21% (-30, 64)	58% (40, 74)	30% (8, 53)
C	0.9	10	18% (6, 28)	22% (2, 40)	19% (9, 28)	20% (-36, 61)	19% (-0, 34)	10% (-11, 31)
C	0.9	20	52% (45, 60)	50% (33, 64)	52% (44, 59)	18% (-41, 62)	40% (21, 56)	21% (-4, 44)
C	0.9	50	86% (82, 90)	73% (60, 84)	82% (77, 87)	22% (-27, 65)	61% (43, 77)	31% (10, 54)
C	0.9	75	91% (87, 95)	73% (61, 84)	86% (81, 90)	22% (-35, 66)	63% (45, 79)	33% (9, 56)
C	1	10	20% (9, 30)	22% (2, 42)	20% (11, 29)	21% (-34, 62)	21% (1, 35)	11% (-10, 32)
C	1	20	57% (50, 63)	54% (39, 68)	56% (49, 63)	21% (-37, 64)	44% (25, 59)	22% (-3, 47)
C	1	50	92% (90, 95)	80% (70, 88)	89% (85, 92)	22% (-29, 62)	65% (47, 79)	34% (9, 55)
C	1	75	97% (95, 98)	81% (69, 91)	92% (88, 95)	25% (-18, 63)	68% (53, 82)	35% (15, 58)
D	0.6	10	11% (-2, 22)	10% (-13, 33)	11% (-1, 21)	19% (-38, 61)	13% (-7, 29)	7% (-14, 28)
D	0.6	20	35% (25, 44)	21% (0, 42)	31% (21, 40)	21% (-34, 62)	27% (6, 43)	14% (-11, 38)
D	0.6	50	56% (48, 63)	29% (6, 48)	47% (38, 55)	17% (-38, 66)	36% (18, 54)	19% (-4, 41)
D	0.6	75	59% (51, 66)	27% (7, 46)	49% (41, 57)	14% (-48, 61)	36% (15, 54)	19% (-4, 42)
D	0.7	10	12% (0, 23)	12% (-10, 34)	12% (1, 22)	20% (-40, 61)	15% (-5, 30)	8% (-13, 30)
D	0.7	20	39% (31, 47)	25% (3, 45)	35% (26, 45)	17% (-37, 62)	28% (8, 45)	15% (-11, 38)
D	0.7	50	66% (58, 73)	33% (14, 50)	56% (48, 63)	22% (-25, 62)	43% (27, 58)	22% (-0, 45)
D	0.7	75	69% (62, 76)	30% (8, 49)	57% (49, 65)	20% (-44, 67)	43% (21, 61)	22% (-4, 48)
D	0.8	10	15% (3, 25)	13% (-10, 34)	14% (4, 24)	20% (-36, 61)	16% (-4, 31)	8% (-13, 30)
D	0.8	20	44% (35, 53)	27% (5, 46)	39% (31, 48)	20% (-27, 62)	32% (15, 47)	17% (-8, 40)
D	0.8	50	75% (69, 80)	39% (19, 55)	64% (57, 71)	20% (-30, 62)	48% (31, 64)	25% (2, 47)
D	0.8	75	77% (71, 83)	36% (13, 53)	65% (57, 72)	21% (-34, 65)	49% (28, 66)	25% (-0, 48)
D	0.9	10	17% (5, 27)	14% (-6, 35)	16% (6, 25)	21% (-33, 61)	17% (-1, 32)	9% (-12, 30)
D	0.9	20	49% (42, 57)	32% (14, 49)	44% (37, 52)	19% (-33, 62)	35% (16, 51)	18% (-6, 41)
D	0.9	50	83% (78, 87)	43% (23, 59)	71% (64, 77)	24% (-21, 62)	54% (36, 68)	28% (5, 49)
D	0.9	75	86% (82, 90)	39% (22, 56)	72% (66, 78)	22% (-25, 64)	54% (35, 69)	28% (4, 51)
D	1	10	18% (8, 29)	15% (-8, 36)	17% (7, 27)	20% (-36, 61)	18% (-2, 33)	9% (-11, 31)
D	1	20	54% (47, 61)	32% (14, 50)	47% (39, 56)	19% (-34, 61)	37% (18, 52)	19% (-5, 42)

D	1	50	89% (86, 93)	47% (30, 62)	77% (71, 82)	24% (-25, 66)	57% (40, 73)	30% (5, 51)
D	1	75	93% (91, 96)	42% (22, 60)	78% (71, 83)	22% (-25, 65)	58% (40, 74)	30% (8, 53)
E	0.6	10	11% (-1, 22)	10% (-12, 33)	11% (-1, 21)	18% (-43, 61)	13% (-8, 29)	7% (-14, 29)
E	0.6	20	35% (25, 44)	21% (1, 42)	31% (21, 40)	16% (-43, 60)	25% (4, 42)	13% (-13, 37)
E	0.6	50	56% (48, 63)	29% (6, 48)	48% (38, 56)	16% (-34, 58)	36% (19, 51)	19% (-4, 40)
E	0.6	75	59% (51, 66)	28% (7, 47)	49% (41, 57)	11% (-44, 59)	36% (16, 53)	18% (-6, 43)
E	0.7	10	12% (1, 23)	12% (-11, 34)	12% (1, 22)	17% (-44, 61)	14% (-7, 30)	7% (-13, 29)
E	0.7	20	39% (30, 47)	25% (3, 44)	35% (26, 45)	16% (-38, 60)	28% (9, 45)	14% (-11, 38)
E	0.7	50	66% (58, 73)	33% (14, 50)	56% (48, 63)	15% (-38, 58)	41% (21, 57)	21% (-1, 43)
E	0.7	75	69% (62, 76)	30% (8, 49)	57% (49, 65)	11% (-43, 58)	40% (20, 58)	21% (-4, 44)
E	0.8	10	15% (3, 25)	13% (-10, 34)	14% (4, 24)	17% (-44, 61)	15% (-6, 31)	8% (-13, 29)
E	0.8	20	44% (35, 53)	27% (5, 46)	39% (31, 48)	17% (-35, 62)	31% (12, 47)	16% (-8, 40)
E	0.8	50	75% (69, 81)	40% (19, 55)	64% (57, 71)	20% (-26, 60)	48% (31, 63)	25% (2, 47)
E	0.8	75	77% (71, 83)	36% (13, 54)	65% (57, 72)	10% (-54, 60)	45% (23, 64)	23% (-1, 47)
E	0.9	10	16% (5, 27)	14% (-6, 35)	16% (6, 25)	18% (-43, 61)	16% (-4, 32)	8% (-12, 30)
E	0.9	20	49% (42, 57)	32% (14, 49)	44% (37, 52)	13% (-47, 60)	33% (12, 50)	17% (-10, 41)
E	0.9	50	83% (78, 87)	43% (23, 59)	71% (64, 77)	16% (-31, 60)	51% (33, 67)	26% (3, 49)
E	0.9	75	86% (82, 90)	38% (22, 55)	72% (66, 77)	15% (-41, 59)	51% (32, 68)	26% (3, 49)
E	1	10	18% (8, 29)	15% (-8, 36)	17% (7, 27)	18% (-43, 61)	17% (-4, 33)	9% (-12, 30)
E	1	20	54% (47, 61)	32% (14, 50)	47% (39, 56)	14% (-41, 59)	35% (15, 51)	18% (-7, 41)
E	1	50	89% (86, 93)	47% (30, 62)	77% (71, 82)	18% (-30, 56)	55% (38, 69)	29% (6, 50)
E	1	75	93% (91, 96)	42% (22, 60)	78% (71, 83)	17% (-33, 59)	56% (37, 72)	29% (5, 51)

Table B3. Relative reduction in nationwide cumulative risk of HR HPV infection and life-time cervical cancer risk by cohort across single-dose protection assumptions
¤ See Table A11 for the exact efficacy and rate of waning for the single-dose protection assumptions.

Single-dose protection scenario ¤	Routine coverage	Birth cohorts by years since start of vaccination to be vaccinated	HPV 16	HPV 18	HPV 31/33/45	LTR of cervical cancer
A	0.6	1-5th	61% (57, 66)	66% (61, 71)	5% (-1, 10)	49% (46, 52)
A	0.6	6-10th	63% (58, 68)	70% (65, 75)	12% (5, 18)	51% (48, 54)
A	0.6	46-50th	75% (71, 79)	85% (79, 91)	10% (3, 17)	61% (58, 64)
A	0.7	1-5th	70% (67, 75)	75% (70, 79)	6% (0, 11)	56% (54, 59)
A	0.7	6-10th	72% (67, 76)	79% (75, 83)	12% (5, 20)	58% (55, 61)
A	0.7	46-50th	87% (83, 90)	94% (90, 98)	11% (2, 19)	70% (67, 72)
A	0.8	1-5th	80% (77, 84)	83% (79, 87)	7% (1, 13)	64% (61, 66)
A	0.8	6-10th	81% (78, 85)	86% (82, 89)	12% (6, 19)	65% (62, 67)
A	0.8	46-50th	95% (92, 97)	99% (98, 100)	13% (4, 20)	76% (74, 77)
A	0.9	1-5th	89% (87, 92)	91% (88, 94)	8% (2, 13)	71% (69, 72)
A	0.9	6-10th	90% (87, 93)	92% (90, 95)	13% (7, 20)	71% (69, 73)
A	0.9	46-50th	99% (98, 100)	100% (100, 100)	17% (7, 23)	79% (78, 79)
A	1	1-5th	98% (96, 99)	98% (97, 99)	9% (4, 14)	77% (76, 78)
A	1	6-10th	97% (95, 98)	97% (96, 99)	14% (5, 22)	76% (75, 78)
A	1	46-50th	100% (100, 100)	100% (100, 100)	17% (9, 24)	79% (79, 80)
B	0.6	1-5th	54% (50, 59)	55% (50, 60)	4% (-1, 8)	43% (40, 46)
B	0.6	6-10th	55% (50, 60)	55% (49, 60)	9% (2, 15)	44% (40, 47)
B	0.6	46-50th	64% (59, 69)	64% (56, 72)	7% (1, 15)	50% (47, 54)
B	0.7	1-5th	61% (57, 65)	65% (60, 71)	5% (-1, 12)	48% (46, 51)
B	0.7	6-10th	63% (58, 67)	67% (61, 72)	8% (1, 16)	50% (47, 53)
B	0.7	46-50th	75% (70, 79)	74% (67, 80)	11% (2, 19)	59% (56, 62)
B	0.8	1-5th	70% (66, 74)	73% (68, 78)	5% (-0, 11)	55% (53, 58)
B	0.8	6-10th	71% (66, 75)	74% (68, 79)	9% (3, 15)	56% (53, 59)
B	0.8	46-50th	84% (80, 88)	86% (81, 92)	12% (3, 19)	67% (64, 70)
B	0.9	1-5th	78% (74, 82)	79% (74, 82)	7% (1, 13)	61% (59, 64)
B	0.9	6-10th	81% (77, 84)	79% (75, 84)	11% (3, 20)	64% (61, 66)
B	0.9	46-50th	93% (90, 96)	96% (91, 99)	11% (3, 19)	74% (72, 76)
B	1	1-5th	86% (84, 89)	85% (82, 88)	8% (3, 13)	68% (66, 69)
B	1	6-10th	87% (83, 90)	87% (83, 91)	12% (4, 20)	68% (66, 71)
B	1	46-50th	98% (97, 99)	99% (98, 100)	13% (5, 21)	78% (77, 79)
C	0.6	1-5th	51% (46, 55)	40% (35, 46)	3% (-2, 9)	38% (35, 41)
C	0.6	6-10th	53% (48, 58)	43% (37, 50)	7% (-1, 13)	40% (37, 43)
C	0.6	46-50th	61% (56, 66)	46% (40, 52)	6% (-1, 14)	46% (42, 49)
C	0.7	1-5th	56% (52, 61)	47% (41, 53)	3% (-2, 9)	43% (40, 46)
C	0.7	6-10th	61% (55, 66)	51% (45, 58)	7% (-0, 13)	46% (42, 50)
C	0.7	46-50th	71% (66, 75)	55% (49, 62)	9% (0, 16)	53% (50, 56)
C	0.8	1-5th	66% (62, 70)	54% (48, 59)	4% (-1, 8)	50% (47, 53)

C	0.8	6-10th	69% (64, 73)	54% (48, 60)	6% (-2, 13)	52% (48, 55)
C	0.8	46-50th	81% (77, 84)	63% (57, 69)	9% (0, 16)	61% (58, 63)
C	0.9	1-5th	74% (70, 78)	58% (52, 63)	5% (-1, 10)	55% (53, 58)
C	0.9	6-10th	78% (74, 82)	59% (53, 65)	10% (2, 17)	58% (56, 61)
C	0.9	46-50th	90% (86, 93)	71% (66, 76)	8% (0, 17)	68% (65, 70)
C	1	1-5th	81% (78, 84)	63% (57, 68)	6% (0, 12)	61% (58, 63)
C	1	6-10th	84% (81, 87)	69% (64, 74)	10% (2, 17)	64% (61, 66)
C	1	46-50th	96% (94, 98)	79% (73, 85)	11% (3, 17)	73% (71, 75)
D	0.6	1-5th	47% (42, 52)	20% (14, 24)	3% (-2, 8)	33% (29, 36)
D	0.6	6-10th	50% (45, 55)	22% (16, 29)	6% (0, 13)	35% (32, 38)
D	0.6	46-50th	57% (52, 61)	25% (18, 32)	7% (-3, 14)	40% (36, 43)
D	0.7	1-5th	52% (48, 56)	26% (22, 31)	3% (-1, 9)	37% (34, 40)
D	0.7	6-10th	57% (51, 62)	27% (21, 33)	6% (-0, 13)	40% (36, 43)
D	0.7	46-50th	67% (62, 72)	29% (24, 36)	8% (-0, 15)	47% (43, 50)
D	0.8	1-5th	60% (56, 65)	28% (22, 32)	3% (-3, 9)	42% (39, 46)
D	0.8	6-10th	65% (60, 70)	28% (22, 34)	6% (-1, 12)	45% (42, 48)
D	0.8	46-50th	76% (71, 80)	35% (29, 42)	8% (-2, 16)	53% (50, 56)
D	0.9	1-5th	69% (65, 72)	31% (27, 36)	4% (-2, 9)	48% (45, 51)
D	0.9	6-10th	73% (69, 77)	32% (27, 39)	7% (1, 14)	51% (48, 54)
D	0.9	46-50th	85% (82, 89)	36% (30, 42)	8% (-0, 15)	59% (57, 62)
D	1	1-5th	76% (73, 80)	33% (28, 38)	5% (-1, 10)	53% (50, 55)
D	1	6-10th	79% (76, 83)	37% (30, 44)	8% (1, 14)	56% (53, 58)
D	1	46-50th	92% (89, 95)	41% (35, 47)	10% (2, 18)	65% (62, 67)
E	0.6	1-5th	47% (42, 52)	20% (14, 24)	-1% (-5, 3)	32% (29, 35)
E	0.6	6-10th	50% (45, 55)	22% (16, 29)	-1% (-7, 5)	34% (31, 37)
E	0.6	46-50th	57% (52, 61)	25% (18, 32)	0% (-7, 9)	39% (36, 42)
E	0.7	1-5th	52% (48, 57)	26% (22, 31)	-1% (-5, 4)	37% (34, 39)
E	0.7	6-10th	57% (51, 62)	27% (21, 33)	-1% (-7, 7)	40% (36, 43)
E	0.7	46-50th	67% (62, 72)	29% (24, 36)	0% (-8, 7)	46% (43, 49)
E	0.8	1-5th	60% (56, 65)	28% (22, 32)	-1% (-7, 4)	42% (39, 45)
E	0.8	6-10th	65% (60, 70)	28% (22, 34)	-1% (-7, 6)	45% (42, 48)
E	0.8	46-50th	76% (72, 80)	35% (29, 42)	0% (-7, 8)	53% (50, 56)
E	0.9	1-5th	69% (65, 72)	31% (27, 36)	-0% (-5, 5)	48% (45, 50)
E	0.9	6-10th	73% (69, 77)	33% (27, 39)	-1% (-8, 5)	50% (47, 53)
E	0.9	46-50th	85% (82, 89)	36% (30, 42)	0% (-9, 9)	59% (56, 61)
E	1	1-5th	76% (73, 79)	33% (29, 38)	-1% (-6, 5)	52% (50, 55)
E	1	6-10th	79% (76, 83)	37% (30, 43)	-1% (-7, 6)	55% (52, 57)
E	1	46-50th	93% (89, 95)	41% (35, 47)	0% (-8, 8)	64% (62, 66)

Table B4. Nationwide age-standardised incidence rate of cervical cancer over time in the base-case scenario
ǂ Cases per 100 000 women-years.
* Threshold included in the UI.
** Below threshold.

Year since start of vaccination	ASIR ǂ
0	11.0 (11.0, 11.0)
5	11.0 (11.0, 11.0)
10	11.0 (11.0, 11.0)
15	10.9 (10.9, 11.0)
20	10.8 (10.8, 10.9)
25	10.5 (10.4, 10.6)
30	9.9 (9.8, 10.1)
35	9.0 (8.8, 9.2)
40	7.8 (7.6, 8.1)
45	6.6 (6.3, 6.9)
50	5.5 (5.2, 5.8)
55	4.3 (4.0, 4.6) *
60	3.5 (3.2, 3.8) **
65	3.0 (2.7, 3.3) **
70	2.8 (2.5, 3.1) **
75	2.7 (2.4, 2.9) **
80	2.6 (2.4, 2.8) **
85	2.5 (2.3, 2.7) **
90	2.5 (2.3, 2.7) **
95	2.4 (2.3, 2.6) **
100	2.4 (2.3, 2.6) **

Table B5. Cervical cancer risk in the long-term across Indian states in the base-case scenario.
See Table A9 for the baseline risk without vaccination.
Ŧ States or groups of states as reported in the 2006 National Behaviour Surveillance Survey of the National AIDS Control Organization of India.[9]
¤ See Table A3 for which Indian states are assigned to high- and low-cancer-incidence clusters.
§ Other North Eastern States include Arunachal Pradesh, Nagaland, Meghalaya, Mizoram, and Tripura.
ł Cases per 100 000 women born.
ǂ Cases per 100 000 women-years.
* Threshold included in the UI.
** Below threshold.

Indian state / group of Indian states Ŧ ¤	LTR ł	SLTR ł	ASIR ǂ
India (all states)	233 (219, 252)	293 (274, 316)	2.4 (2.2, 2.6) **
Low incidence	202 (189, 217)	253 (237, 273) *	2.1 (1.9, 2.2) **
High incidence	346 (324, 374)	434 (406, 468)	3.6 (3.3, 3.8) **
Andhra Pradesh	253 (237, 273)	324 (303, 349)	2.5 (2.4, 2.7) **
Assam	186 (174, 200)	231 (216, 249) **	1.9 (1.8, 2.1) **
Bihar	190 (178, 205)	238 (223, 257) *	2.0 (1.8, 2.1) **
Chhattisgarh	190 (178, 205)	238 (223, 257) *	2.0 (1.8, 2.1) **
Delhi	296 (277, 319)	377 (353, 406)	3.0 (2.8, 3.2) **
Goa + Daman & Diu	190 (178, 205)	238 (223, 257) *	2.0 (1.8, 2.1) **
Gujarat + Dadra & Nagar Haveli	190 (178, 205)	233 (219, 252) *	2.0 (1.9, 2.2) **
Haryana	190 (178, 205)	238 (223, 257) *	2.0 (1.8, 2.1) **
Himachal Pradesh	190 (178, 205)	238 (223, 257) *	2.0 (1.8, 2.1) **
Jammu & Kashmir	190 (178, 205)	238 (223, 257) *	2.0 (1.8, 2.1) **
Jharkhand	190 (178, 205)	238 (223, 257) *	2.0 (1.8, 2.1) **
Karnataka	365 (342, 393)	463 (434, 499)	3.7 (3.4, 4.0) **
Kerala + Lakshadweep	161 (151, 174)	207 (194, 223) **	1.6 (1.5, 1.7) **
Madhya Pradesh	328 (307, 353)	412 (386, 444)	3.3 (3.1, 3.6) **
Maharashtra	253 (237, 273)	320 (300, 345)	2.6 (2.4, 2.8) **
Manipur	176 (165, 190)	228 (214, 246) **	1.7 (1.6, 1.9) **
Orissa	190 (178, 205)	238 (223, 257) *	2.0 (1.8, 2.1) **
Other North Eastern States §	324 (304, 350)	391 (366, 422)	3.6 (3.3, 3.9) **
Punjab + Chandigarh	285 (267, 308)	359 (337, 388)	2.9 (2.8, 3.2) **
Rajasthan	190 (178, 205)	238 (223, 257) *	2.0 (1.8, 2.1) **
Sikkim	179 (168, 193)	209 (196, 226) **	2.0 (1.9, 2.2) **
Tamil Nadu + Puducherry	386 (361, 416)	477 (447, 514)	4.0 (3.8, 4.4) *
Uttar Pradesh	190 (178, 205)	238 (223, 257) *	2.0 (1.8, 2.1) **
Uttarakhand	190 (178, 205)	238 (223, 257) *	2.0 (1.8, 2.1) **
West Bengal + Andaman & Nicobar Islands	184 (173, 199)	234 (220, 253) *	1.9 (1.8, 2.0) **

Table B6. Age-standardised incidence rate of cervical cancer in the long-term across single-dose protection assumptions

Age-standardised incidence rate given in cases per 100 000 women-years in the long-term, i.e., in the birth cohorts vaccinated 46-50 years since start of vaccination, under 60-100% vaccination coverage.

° See Table A11 for the exact efficacy and rate of waning for the single-dose protection assumptions.

Ŧ States or groups of states as reported in the 2006 National Behaviour Surveillance Survey of the National AIDS Control Organization of India.[9]

¤ See Table A3 for which Indian states are assigned to high- and low-cancer-incidence clusters.

§ Other North Eastern States include Arunachal Pradesh, Nagaland, Meghalaya, Mizoram, and Tripura.

* Threshold included in the UI.

** Below threshold.

Single-dose protection assumption°	Indian state / group of Indian states Ŧ ¤ §	60%	70%	80%	90%	100%
A	India (all states)	4.5 (4.0, 5.0) *	3.5 (3.1, 4.0) **	2.8 (2.5, 3.1) **	2.4 (2.3, 2.6) **	2.3 (2.2, 2.4) **
A	Low incidence	3.9 (3.4, 4.3) *	3.1 (2.7, 3.4) **	2.4 (2.2, 2.7) **	2.1 (2.0, 2.2) **	2.0 (1.9, 2.0) **
A	High incidence	6.7 (5.9, 7.5)	5.2 (4.6, 5.9)	4.1 (3.7, 4.7) *	3.6 (3.4, 3.8) **	3.4 (3.3, 3.5) **
A	Kerala + Lakshadweep	3.0 (2.6, 3.3) **	2.3 (2.1, 2.6) **	1.8 (1.7, 2.1) **	1.6 (1.5, 1.7) **	1.5 (1.5, 1.6) **
A	Manipur	3.2 (2.9, 3.6) **	2.5 (2.2, 2.9) **	2.0 (1.8, 2.3) **	1.7 (1.6, 1.9) **	1.7 (1.6, 1.7) **
A	Sikkim	3.8 (3.3, 4.2) *	3.0 (2.6, 3.3) **	2.3 (2.1, 2.6) **	2.0 (1.9, 2.2) **	1.9 (1.9, 2.0) **
A	West Bengal + Andaman & Nicobar Islands	3.5 (3.1, 3.9) **	2.8 (2.4, 3.1) **	2.2 (2.0, 2.5) **	1.9 (1.8, 2.0) **	1.8 (1.8, 1.9) **
A	Assam	3.7 (3.2, 4.1) *	2.9 (2.5, 3.2) **	2.2 (2.0, 2.5) **	1.9 (1.8, 2.1) **	1.9 (1.8, 1.9) **
A	Gujarat + Dadra & Nagar Haveli	3.8 (3.4, 4.3) *	3.0 (2.6, 3.4) **	2.3 (2.1, 2.7) **	2.0 (1.9, 2.2) **	1.9 (1.9, 2.0) **
A	Bihar	3.7 (3.3, 4.1) *	2.9 (2.6, 3.3) **	2.3 (2.1, 2.6) **	2.0 (1.9, 2.1) **	1.9 (1.8, 1.9) **
A	Chhattisgarh	3.7 (3.3, 4.1) *	2.9 (2.6, 3.3) **	2.3 (2.1, 2.6) **	2.0 (1.9, 2.1) **	1.9 (1.8, 1.9) **
A	Goa + Daman & Diu	3.7 (3.3, 4.1) *	2.9 (2.6, 3.3) **	2.3 (2.1, 2.6) **	2.0 (1.9, 2.1) **	1.9 (1.8, 1.9) **
A	Haryana	3.7 (3.3, 4.1) *	2.9 (2.6, 3.3) **	2.3 (2.1, 2.6) **	2.0 (1.9, 2.1) **	1.9 (1.8, 1.9) **
A	Himachal Pradesh	3.7 (3.3, 4.1) *	2.9 (2.6, 3.3) **	2.3 (2.1, 2.6) **	2.0 (1.9, 2.1) **	1.9 (1.8, 1.9) **
A	Jammu & Kashmir	3.7 (3.3, 4.1) *	2.9 (2.6, 3.3) **	2.3 (2.1, 2.6) **	2.0 (1.9, 2.1) **	1.9 (1.8, 1.9) **
A	Jharkhand	3.7 (3.3, 4.1) *	2.9 (2.6, 3.3) **	2.3 (2.1, 2.6) **	2.0 (1.9, 2.1) **	1.9 (1.8, 1.9) **
A	Orissa	3.7 (3.3, 4.1) *	2.9 (2.6, 3.3) **	2.3 (2.1, 2.6) **	2.0 (1.9, 2.1) **	1.9 (1.8, 1.9) **
A	Rajasthan	3.7 (3.3, 4.1) *	2.9 (2.6, 3.3) **	2.3 (2.1, 2.6) **	2.0 (1.9, 2.1) **	1.9 (1.8, 1.9) **
A	Uttar Pradesh	3.7 (3.3, 4.1) *	2.9 (2.6, 3.3) **	2.3 (2.1, 2.6) **	2.0 (1.9, 2.1) **	1.9 (1.8, 1.9) **
A	Uttarakhand	3.7 (3.3, 4.1) *	2.9 (2.6, 3.3) **	2.3 (2.1, 2.6) **	2.0 (1.9, 2.1) **	1.9 (1.8, 1.9) **
A	Andhra Pradesh	4.7 (4.2, 5.3)	3.7 (3.3, 4.2) *	2.9 (2.6, 3.3) **	2.5 (2.4, 2.7) **	2.4 (2.4, 2.5) **
A	Maharashtra	4.9 (4.3, 5.4)	3.8 (3.4, 4.3) *	3.0 (2.7, 3.4) **	2.6 (2.4, 2.8) **	2.5 (2.4, 2.6) **
A	Punjab + Chandigarh	5.5 (4.9, 6.2)	4.3 (3.8, 4.9) *	3.4 (3.1, 3.8) **	2.9 (2.8, 3.1) **	2.8 (2.7, 2.9) **
A	Delhi	5.6 (5.0, 6.3)	4.4 (3.9, 5.0) *	3.5 (3.1, 3.9) **	3.0 (2.8, 3.2) **	2.9 (2.8, 3.0) **
A	Other North Eastern States	6.7 (5.9, 7.5)	5.3 (4.6, 5.9)	4.1 (3.7, 4.7) *	3.6 (3.4, 3.8) **	3.4 (3.3, 3.5) **
A	Madhya Pradesh	6.3 (5.6, 7.0)	4.9 (4.3, 5.6)	3.9 (3.5, 4.4) *	3.3 (3.2, 3.6) **	3.2 (3.1, 3.3) **
A	Karnataka	6.9 (6.1, 7.7)	5.4 (4.8, 6.1)	4.2 (3.8, 4.8) *	3.7 (3.5, 3.9) **	3.5 (3.4, 3.6) **
A	Tamil Nadu + Puducherry	7.6 (6.7, 8.5)	5.9 (5.2, 6.7)	4.7 (4.2, 5.3)	4.0 (3.8, 4.3) *	3.9 (3.8, 4.0) **
B	India (all states)	5.7 (5.1, 6.2)	4.7 (4.3, 5.1)	3.8 (3.3, 4.3) *	3.0 (2.6, 3.5) **	2.5 (2.3, 2.7) **

B	Low incidence	4.9 (4.4, 5.4)	4.0 (3.7, 4.4) *	3.3 (2.9, 3.7) **	2.6 (2.2, 3.0) **	2.1 (2.0, 2.3) **
B	High incidence	8.4 (7.6, 9.2)	6.9 (6.3, 7.6)	5.7 (4.9, 6.4)	4.5 (3.8, 5.2) *	3.7 (3.4, 4.0) *
B	Kerala + Lakshadweep	3.7 (3.4, 4.1) *	3.1 (2.8, 3.4) **	2.5 (2.2, 2.8) **	2.0 (1.7, 2.3) **	1.6 (1.5, 1.8) **
B	Manipur	4.1 (3.7, 4.5) *	3.4 (3.1, 3.7) **	2.7 (2.4, 3.1) **	2.2 (1.9, 2.5) **	1.8 (1.6, 1.9) **
B	Sikkim	4.7 (4.3, 5.2)	3.9 (3.6, 4.3) *	3.2 (2.8, 3.6) **	2.5 (2.2, 2.9) **	2.1 (1.9, 2.3) **
B	West Bengal + Andaman & Nicobar Islands	4.4 (4.0, 4.9) *	3.7 (3.3, 4.0) *	3.0 (2.6, 3.4) **	2.3 (2.0, 2.7) **	1.9 (1.8, 2.1) **
B	Assam	4.6 (4.1, 5.0)	3.8 (3.4, 4.2) *	3.1 (2.7, 3.5) **	2.4 (2.1, 2.8) **	2.0 (1.9, 2.2) **
B	Gujarat + Dadra & Nagar Haveli	4.8 (4.3, 5.3)	4.0 (3.6, 4.4) *	3.2 (2.8, 3.6) **	2.5 (2.2, 2.9) **	2.1 (1.9, 2.3) **
B	Bihar	4.6 (4.2, 5.1)	3.8 (3.5, 4.2) *	3.1 (2.7, 3.5) **	2.5 (2.1, 2.8) **	2.0 (1.9, 2.2) **
B	Chhattisgarh	4.6 (4.2, 5.1)	3.8 (3.5, 4.2) *	3.1 (2.7, 3.5) **	2.5 (2.1, 2.8) **	2.0 (1.9, 2.2) **
B	Goa + Daman & Diu	4.6 (4.2, 5.1)	3.8 (3.5, 4.2) *	3.1 (2.7, 3.5) **	2.5 (2.1, 2.8) **	2.0 (1.9, 2.2) **
B	Haryana	4.6 (4.2, 5.1)	3.8 (3.5, 4.2) *	3.1 (2.7, 3.5) **	2.5 (2.1, 2.8) **	2.0 (1.9, 2.2) **
B	Himachal Pradesh	4.6 (4.2, 5.1)	3.8 (3.5, 4.2) *	3.1 (2.7, 3.5) **	2.5 (2.1, 2.8) **	2.0 (1.9, 2.2) **
B	Jammu & Kashmir	4.6 (4.2, 5.1)	3.8 (3.5, 4.2) *	3.1 (2.7, 3.5) **	2.5 (2.1, 2.8) **	2.0 (1.9, 2.2) **
B	Jharkhand	4.6 (4.2, 5.1)	3.8 (3.5, 4.2) *	3.1 (2.7, 3.5) **	2.5 (2.1, 2.8) **	2.0 (1.9, 2.2) **
B	Orissa	4.6 (4.2, 5.1)	3.8 (3.5, 4.2) *	3.1 (2.7, 3.5) **	2.5 (2.1, 2.8) **	2.0 (1.9, 2.2) **
B	Rajasthan	4.6 (4.2, 5.1)	3.8 (3.5, 4.2) *	3.1 (2.7, 3.5) **	2.5 (2.1, 2.8) **	2.0 (1.9, 2.2) **
B	Uttar Pradesh	4.6 (4.2, 5.1)	3.8 (3.5, 4.2) *	3.1 (2.7, 3.5) **	2.5 (2.1, 2.8) **	2.0 (1.9, 2.2) **
B	Uttarakhand	4.6 (4.2, 5.1)	3.8 (3.5, 4.2) *	3.1 (2.7, 3.5) **	2.5 (2.1, 2.8) **	2.0 (1.9, 2.2) **
B	Andhra Pradesh	5.9 (5.4, 6.5)	4.9 (4.5, 5.4)	4.0 (3.5, 4.5) *	3.1 (2.7, 3.6) **	2.6 (2.4, 2.8) **
B	Maharashtra	6.1 (5.5, 6.7)	5.0 (4.6, 5.5)	4.1 (3.6, 4.6) *	3.2 (2.8, 3.7) **	2.7 (2.5, 2.9) **
B	Punjab + Chandigarh	6.9 (6.3, 7.6)	5.7 (5.2, 6.3)	4.7 (4.1, 5.2)	3.7 (3.2, 4.2) *	3.0 (2.8, 3.3) **
B	Delhi	7.1 (6.4, 7.8)	5.8 (5.3, 6.4)	4.7 (4.1, 5.4)	3.7 (3.2, 4.3) *	3.1 (2.9, 3.4) **
B	Other North Eastern States	8.4 (7.6, 9.3)	7.0 (6.3, 7.6)	5.7 (4.9, 6.4)	4.5 (3.8, 5.2) *	3.7 (3.4, 4.0) *
B	Madhya Pradesh	7.9 (7.1, 8.7)	6.5 (5.9, 7.2)	5.3 (4.6, 6.0)	4.2 (3.6, 4.8) *	3.4 (3.2, 3.8) **
B	Karnataka	8.7 (7.8, 9.5)	7.2 (6.5, 7.9)	5.8 (5.1, 6.6)	4.6 (4.0, 5.3) *	3.8 (3.5, 4.1) *
B	Tamil Nadu + Puducherry	9.5 (8.6, 10.5)	7.9 (7.2, 8.6)	6.4 (5.6, 7.2)	5.0 (4.3, 5.8)	4.2 (3.8, 4.5) *
C	India (all states)	6.2 (5.6, 6.7)	5.3 (4.8, 5.8)	4.5 (4.0, 5.0)	3.7 (3.2, 4.2) *	3.1 (2.7, 3.4) **
C	Low incidence	5.3 (4.9, 5.8)	4.6 (4.1, 5.0)	3.9 (3.5, 4.3) *	3.2 (2.7, 3.6) **	2.7 (2.4, 3.0) **
C	High incidence	9.2 (8.3, 10.0)	7.8 (7.1, 8.6)	6.7 (6.0, 7.4)	5.5 (4.7, 6.2)	4.6 (4.1, 5.1)
C	Kerala + Lakshadweep	4.1 (3.7, 4.5) *	3.5 (3.2, 3.8) **	3.0 (2.7, 3.3) **	2.4 (2.1, 2.8) **	2.0 (1.8, 2.3) **
C	Manipur	4.4 (4.0, 4.9)	3.8 (3.5, 4.2) *	3.2 (2.9, 3.6) **	2.6 (2.3, 3.0) **	2.2 (2.0, 2.5) **
C	Sikkim	5.2 (4.7, 5.7)	4.4 (4.0, 4.9)	3.8 (3.4, 4.2) *	3.1 (2.7, 3.5) **	2.6 (2.3, 2.9) **
C	West Bengal + Andaman & Nicobar Islands	4.8 (4.4, 5.3)	4.1 (3.7, 4.5) *	3.5 (3.2, 3.9) **	2.9 (2.5, 3.3) **	2.4 (2.1, 2.7) **
C	Assam	5.0 (4.6, 5.5)	4.3 (3.9, 4.7) *	3.6 (3.3, 4.0) *	3.0 (2.6, 3.4) **	2.5 (2.2, 2.8) **
C	Gujarat + Dadra & Nagar Haveli	5.2 (4.8, 5.7)	4.5 (4.1, 4.9)	3.8 (3.4, 4.2) *	3.1 (2.7, 3.5) **	2.6 (2.3, 2.9) **
C	Bihar	5.1 (4.6, 5.5)	4.3 (3.9, 4.8) *	3.7 (3.3, 4.1) *	3.0 (2.6, 3.4) **	2.5 (2.3, 2.8) **
C	Chhattisgarh	5.1 (4.6, 5.5)	4.3 (3.9, 4.8) *	3.7 (3.3, 4.1) *	3.0 (2.6, 3.4) **	2.5 (2.3, 2.8) **
C	Goa + Daman & Diu	5.1 (4.6, 5.5)	4.3 (3.9, 4.8) *	3.7 (3.3, 4.1) *	3.0 (2.6, 3.4) **	2.5 (2.3, 2.8) **
C	Haryana	5.1 (4.6, 5.5)	4.3 (3.9, 4.8) *	3.7 (3.3, 4.1) *	3.0 (2.6, 3.4) **	2.5 (2.3, 2.8) **
C	Himachal Pradesh	5.1 (4.6, 5.5)	4.3 (3.9, 4.8) *	3.7 (3.3, 4.1) *	3.0 (2.6, 3.4) **	2.5 (2.3, 2.8) **

C	Jammu & Kashmir	5.1 (4.6, 5.5)	4.3 (3.9, 4.8) *	3.7 (3.3, 4.1) *	3.0 (2.6, 3.4) **	2.5 (2.3, 2.8) **
C	Jharkhand	5.1 (4.6, 5.5)	4.3 (3.9, 4.8) *	3.7 (3.3, 4.1) *	3.0 (2.6, 3.4) **	2.5 (2.3, 2.8) **
C	Orissa	5.1 (4.6, 5.5)	4.3 (3.9, 4.8) *	3.7 (3.3, 4.1) *	3.0 (2.6, 3.4) **	2.5 (2.3, 2.8) **
C	Rajasthan	5.1 (4.6, 5.5)	4.3 (3.9, 4.8) *	3.7 (3.3, 4.1) *	3.0 (2.6, 3.4) **	2.5 (2.3, 2.8) **
C	Uttar Pradesh	5.1 (4.6, 5.5)	4.3 (3.9, 4.8) *	3.7 (3.3, 4.1) *	3.0 (2.6, 3.4) **	2.5 (2.3, 2.8) **
C	Uttarakhand	5.1 (4.6, 5.5)	4.3 (3.9, 4.8) *	3.7 (3.3, 4.1) *	3.0 (2.6, 3.4) **	2.5 (2.3, 2.8) **
C	Andhra Pradesh	6.5 (5.9, 7.1)	5.5 (5.0, 6.1)	4.7 (4.2, 5.2)	3.9 (3.3, 4.4) *	3.2 (2.9, 3.6) **
C	Maharashtra	6.7 (6.1, 7.3)	5.7 (5.2, 6.2)	4.9 (4.4, 5.3)	4.0 (3.4, 4.5) *	3.3 (3.0, 3.7) **
C	Punjab + Chandigarh	7.6 (6.9, 8.3)	6.5 (5.9, 7.1)	5.5 (5.0, 6.1)	4.5 (3.9, 5.1) *	3.8 (3.4, 4.2) *
C	Delhi	7.7 (7.0, 8.4)	6.6 (6.0, 7.2)	5.6 (5.1, 6.2)	4.6 (4.0, 5.2) *	3.8 (3.4, 4.3) *
C	Other North Eastern States	9.2 (8.4, 10.0)	7.9 (7.1, 8.6)	6.7 (6.0, 7.4)	5.5 (4.7, 6.2)	4.6 (4.1, 5.1)
C	Madhya Pradesh	8.6 (7.8, 9.4)	7.4 (6.7, 8.1)	6.3 (5.6, 6.9)	5.1 (4.4, 5.8)	4.3 (3.8, 4.8) *
C	Karnataka	9.5 (8.6, 10.3)	8.1 (7.3, 8.9)	6.9 (6.2, 7.6)	5.6 (4.9, 6.4)	4.7 (4.2, 5.3)
C	Tamil Nadu + Puducherry	10.4 (9.5, 11.3)	8.9 (8.1, 9.7)	7.6 (6.8, 8.3)	6.2 (5.4, 7.0)	5.2 (4.6, 5.8)
D	India (all states)	6.9 (6.3, 7.5)	6.1 (5.6, 6.7)	5.5 (4.9, 6.0)	4.6 (4.2, 5.1)	4.1 (3.6, 4.6) *
D	Low incidence	5.9 (5.4, 6.4)	5.3 (4.8, 5.8)	4.7 (4.3, 5.2)	4.0 (3.6, 4.4) *	3.5 (3.1, 3.9) **
D	High incidence	10.2 (9.3, 11.1)	9.1 (8.3, 9.9)	8.1 (7.3, 8.9)	6.9 (6.2, 7.6)	6.0 (5.4, 6.8)
D	Kerala + Lakshadweep	4.5 (4.2, 4.9)	4.0 (3.7, 4.4) *	3.6 (3.3, 3.9) **	3.1 (2.7, 3.4) **	2.7 (2.4, 3.0) **
D	Manipur	4.9 (4.5, 5.4)	4.4 (4.0, 4.8)	3.9 (3.5, 4.3) *	3.3 (3.0, 3.7) **	2.9 (2.6, 3.3) **
D	Sikkim	5.8 (5.3, 6.3)	5.1 (4.7, 5.6)	4.6 (4.1, 5.0)	3.9 (3.5, 4.3) *	3.4 (3.0, 3.8) **
D	West Bengal + Andaman & Nicobar Islands	5.4 (4.9, 5.8)	4.8 (4.3, 5.2)	4.3 (3.9, 4.7) *	3.6 (3.2, 4.0) *	3.2 (2.8, 3.6) **
D	Assam	5.6 (5.1, 6.0)	5.0 (4.5, 5.4)	4.4 (4.0, 4.8) *	3.8 (3.4, 4.2) *	3.3 (2.9, 3.7) **
D	Gujarat + Dadra & Nagar Haveli	5.8 (5.3, 6.3)	5.2 (4.7, 5.7)	4.6 (4.2, 5.1)	3.9 (3.5, 4.3) *	3.4 (3.1, 3.9) **
D	Bihar	5.6 (5.2, 6.1)	5.0 (4.6, 5.5)	4.5 (4.0, 4.9)	3.8 (3.4, 4.2) *	3.3 (3.0, 3.7) **
D	Chhattisgarh	5.6 (5.2, 6.1)	5.0 (4.6, 5.5)	4.5 (4.0, 4.9)	3.8 (3.4, 4.2) *	3.3 (3.0, 3.7) **
D	Goa + Daman & Diu	5.6 (5.2, 6.1)	5.0 (4.6, 5.5)	4.5 (4.0, 4.9)	3.8 (3.4, 4.2) *	3.3 (3.0, 3.7) **
D	Haryana	5.6 (5.2, 6.1)	5.0 (4.6, 5.5)	4.5 (4.0, 4.9)	3.8 (3.4, 4.2) *	3.3 (3.0, 3.7) **
D	Himachal Pradesh	5.6 (5.2, 6.1)	5.0 (4.6, 5.5)	4.5 (4.0, 4.9)	3.8 (3.4, 4.2) *	3.3 (3.0, 3.7) **
D	Jammu & Kashmir	5.6 (5.2, 6.1)	5.0 (4.6, 5.5)	4.5 (4.0, 4.9)	3.8 (3.4, 4.2) *	3.3 (3.0, 3.7) **
D	Jharkhand	5.6 (5.2, 6.1)	5.0 (4.6, 5.5)	4.5 (4.0, 4.9)	3.8 (3.4, 4.2) *	3.3 (3.0, 3.7) **
D	Orissa	5.6 (5.2, 6.1)	5.0 (4.6, 5.5)	4.5 (4.0, 4.9)	3.8 (3.4, 4.2) *	3.3 (3.0, 3.7) **
D	Rajasthan	5.6 (5.2, 6.1)	5.0 (4.6, 5.5)	4.5 (4.0, 4.9)	3.8 (3.4, 4.2) *	3.3 (3.0, 3.7) **
D	Uttar Pradesh	5.6 (5.2, 6.1)	5.0 (4.6, 5.5)	4.5 (4.0, 4.9)	3.8 (3.4, 4.2) *	3.3 (3.0, 3.7) **
D	Uttarakhand	5.6 (5.2, 6.1)	5.0 (4.6, 5.5)	4.5 (4.0, 4.9)	3.8 (3.4, 4.2) *	3.3 (3.0, 3.7) **
D	Andhra Pradesh	7.2 (6.6, 7.8)	6.4 (5.8, 7.0)	5.7 (5.2, 6.3)	4.9 (4.4, 5.4)	4.3 (3.8, 4.8) *
D	Maharashtra	7.4 (6.8, 8.1)	6.6 (6.0, 7.2)	5.9 (5.3, 6.4)	5.0 (4.5, 5.5)	4.4 (3.9, 4.9) *
D	Punjab + Chandigarh	8.4 (7.7, 9.1)	7.5 (6.8, 8.2)	6.7 (6.0, 7.3)	5.7 (5.1, 6.3)	5.0 (4.4, 5.6)
D	Delhi	8.6 (7.8, 9.3)	7.6 (6.9, 8.4)	6.8 (6.2, 7.5)	5.8 (5.2, 6.4)	5.1 (4.5, 5.7)
D	Other North Eastern States	10.2 (9.3, 11.1)	9.1 (8.3, 10.0)	8.1 (7.3, 8.9)	6.9 (6.2, 7.6)	6.1 (5.4, 6.8)
D	Madhya Pradesh	9.6 (8.8, 10.4)	8.5 (7.8, 9.3)	7.6 (6.9, 8.3)	6.5 (5.8, 7.2)	5.7 (5.1, 6.4)
D	Karnataka	10.5 (9.6, 11.4)	9.4 (8.5, 10.2)	8.4 (7.5, 9.1)	7.1 (6.4, 7.9)	6.2 (5.6, 7.0)

D	Tamil Nadu + Puducherry	11.5 (10.6, 12.5)	10.3 (9.4, 11.3)	9.2 (8.3, 10.0)	7.8 (7.0, 8.6)	6.8 (6.1, 7.7)
E	India (all states)	6.9 (6.3, 7.4)	6.2 (5.6, 6.7)	5.5 (5.0, 6.0)	4.7 (4.2, 5.2)	4.1 (3.7, 4.6) *
E	Low incidence	5.9 (5.4, 6.4)	5.3 (4.9, 5.8)	4.8 (4.3, 5.2)	4.1 (3.6, 4.5) *	3.6 (3.2, 4.0) *
E	High incidence	10.2 (9.4, 11.1)	9.2 (8.4, 10.0)	8.2 (7.4, 8.9)	7.0 (6.2, 7.7)	6.1 (5.5, 6.9)
E	Kerala + Lakshadweep	4.6 (4.2, 4.9)	4.1 (3.7, 4.4) *	3.6 (3.3, 4.0) **	3.1 (2.8, 3.4) **	2.7 (2.4, 3.1) **
E	Manipur	5.0 (4.5, 5.4)	4.4 (4.1, 4.8)	4.0 (3.6, 4.3) *	3.4 (3.0, 3.8) **	3.0 (2.7, 3.3) **
E	Sikkim	5.8 (5.3, 6.2)	5.2 (4.7, 5.6)	4.6 (4.2, 5.0)	3.9 (3.5, 4.4) *	3.5 (3.1, 3.9) **
E	West Bengal + Andaman & Nicobar Islands	5.4 (4.9, 5.8)	4.8 (4.4, 5.2)	4.3 (3.9, 4.7) *	3.7 (3.3, 4.1) *	3.2 (2.9, 3.6) **
E	Assam	5.6 (5.1, 6.0)	5.0 (4.6, 5.4)	4.5 (4.0, 4.9)	3.8 (3.4, 4.2) *	3.4 (3.0, 3.8) **
E	Gujarat + Dadra & Nagar Haveli	5.8 (5.3, 6.3)	5.2 (4.8, 5.7)	4.7 (4.2, 5.1)	4.0 (3.6, 4.4) *	3.5 (3.1, 3.9) **
E	Bihar	5.7 (5.2, 6.1)	5.1 (4.6, 5.5)	4.5 (4.1, 4.9)	3.9 (3.4, 4.3) *	3.4 (3.0, 3.8) **
E	Chhattisgarh	5.7 (5.2, 6.1)	5.1 (4.6, 5.5)	4.5 (4.1, 4.9)	3.9 (3.4, 4.3) *	3.4 (3.0, 3.8) **
E	Goa + Daman & Diu	5.7 (5.2, 6.1)	5.1 (4.6, 5.5)	4.5 (4.1, 4.9)	3.9 (3.4, 4.3) *	3.4 (3.0, 3.8) **
E	Haryana	5.7 (5.2, 6.1)	5.1 (4.6, 5.5)	4.5 (4.1, 4.9)	3.9 (3.4, 4.3) *	3.4 (3.0, 3.8) **
E	Himachal Pradesh	5.7 (5.2, 6.1)	5.1 (4.6, 5.5)	4.5 (4.1, 4.9)	3.9 (3.4, 4.3) *	3.4 (3.0, 3.8) **
E	Jammu & Kashmir	5.7 (5.2, 6.1)	5.1 (4.6, 5.5)	4.5 (4.1, 4.9)	3.9 (3.4, 4.3) *	3.4 (3.0, 3.8) **
E	Jharkhand	5.7 (5.2, 6.1)	5.1 (4.6, 5.5)	4.5 (4.1, 4.9)	3.9 (3.4, 4.3) *	3.4 (3.0, 3.8) **
E	Orissa	5.7 (5.2, 6.1)	5.1 (4.6, 5.5)	4.5 (4.1, 4.9)	3.9 (3.4, 4.3) *	3.4 (3.0, 3.8) **
E	Rajasthan	5.7 (5.2, 6.1)	5.1 (4.6, 5.5)	4.5 (4.1, 4.9)	3.9 (3.4, 4.3) *	3.4 (3.0, 3.8) **
E	Uttar Pradesh	5.7 (5.2, 6.1)	5.1 (4.6, 5.5)	4.5 (4.1, 4.9)	3.9 (3.4, 4.3) *	3.4 (3.0, 3.8) **
E	Uttarakhand	5.7 (5.2, 6.1)	5.1 (4.6, 5.5)	4.5 (4.1, 4.9)	3.9 (3.4, 4.3) *	3.4 (3.0, 3.8) **
E	Andhra Pradesh	7.2 (6.6, 7.8)	6.5 (5.9, 7.0)	5.8 (5.2, 6.3)	4.9 (4.4, 5.5)	4.3 (3.9, 4.9) *
E	Maharashtra	7.4 (6.8, 8.0)	6.7 (6.1, 7.2)	5.9 (5.4, 6.5)	5.1 (4.5, 5.6)	4.5 (4.0, 5.0) *
E	Punjab + Chandigarh	8.4 (7.7, 9.1)	7.6 (6.9, 8.2)	6.7 (6.1, 7.3)	5.7 (5.1, 6.4)	5.1 (4.5, 5.7)
E	Delhi	8.6 (7.9, 9.3)	7.7 (7.0, 8.4)	6.9 (6.2, 7.5)	5.9 (5.2, 6.5)	5.2 (4.6, 5.8)
E	Other North Eastern States	10.2 (9.4, 11.1)	9.2 (8.4, 10.0)	8.2 (7.4, 8.9)	7.0 (6.2, 7.8)	6.2 (5.5, 6.9)
E	Madhya Pradesh	9.6 (8.8, 10.4)	8.6 (7.9, 9.4)	7.7 (6.9, 8.4)	6.5 (5.8, 7.3)	5.8 (5.2, 6.5)
E	Karnataka	10.5 (9.7, 11.4)	9.4 (8.6, 10.3)	8.4 (7.6, 9.2)	7.2 (6.4, 8.0)	6.3 (5.7, 7.1)
E	Tamil Nadu + Puducherry	11.6 (10.6, 12.5)	10.4 (9.5, 11.3)	9.3 (8.4, 10.1)	7.9 (7.1, 8.8)	7.0 (6.2, 7.8)

Table B7. Standardised life-time risk of cervical cancer in the long-term across single-dose protection assumptions

Standardised life-time risk given in cases per 100 000 women born in the long-term, i.e., in the birth cohorts vaccinated 46-50 years since start of vaccination, under 60-100% vaccination coverage.

° See Table A11 for the exact efficacy and rate of waning for the single-dose protection assumptions.

Ŧ States or groups of states as reported in the 2006 National Behaviour Surveillance Survey of the National AIDS Control Organization of India.[9]

¤ See Table A3 for which Indian states are assigned to high- and low-cancer-incidence clusters.

§ Other North Eastern States include Arunachal Pradesh, Nagaland, Meghalaya, Mizoram, and Tripura.

* Threshold included in the UI.

** Below threshold.

Single-dose protection assumption°	Indian state / group of Indian states Ŧ ¤ §	60%	70%	80%	90%	100%
A	India (all states)	550 (486, 614)	431 (380, 486)	338 (305, 382)	292 (276, 314)	280 (274, 289)
A	Low incidence	476 (420, 531)	373 (328, 421)	292 (264, 331)	252 (238, 272) *	242 (237, 250) **
A	High incidence	815 (720, 909)	638 (563, 720)	501 (451, 566)	432 (408, 465)	415 (405, 428)
A	Kerala + Lakshadweep	388 (343, 433)	304 (268, 343)	238 (215, 270) *	206 (194, 221) **	198 (193, 204) **
A	Manipur	429 (379, 478)	336 (296, 379)	263 (238, 298) *	227 (215, 245) **	218 (213, 225) **
A	Sikkim	393 (347, 438)	308 (271, 347)	241 (218, 273) *	208 (197, 224) **	200 (195, 206) **
A	West Bengal + Andaman & Nicobar Islands	440 (389, 491)	345 (304, 389)	271 (244, 306) *	234 (221, 251) *	224 (219, 231) **
A	Assam	433 (383, 483)	339 (299, 383)	266 (240, 301) *	230 (217, 247) **	221 (215, 227) **
A	Gujarat + Dadra & Nagar Haveli	438 (387, 489)	343 (303, 388)	269 (243, 305) *	232 (220, 250) *	223 (218, 230) **
A	Bihar	447 (395, 498)	350 (308, 395)	274 (248, 310) *	237 (224, 255) *	228 (222, 234) **
A	Chhattisgarh	447 (395, 498)	350 (308, 395)	274 (248, 310) *	237 (224, 255) *	228 (222, 234) **
A	Goa + Daman & Diu	447 (395, 498)	350 (308, 395)	274 (248, 310) *	237 (224, 255) *	228 (222, 234) **
A	Haryana	447 (395, 498)	350 (308, 395)	274 (248, 310) *	237 (224, 255) *	228 (222, 234) **
A	Himachal Pradesh	447 (395, 498)	350 (308, 395)	274 (248, 310) *	237 (224, 255) *	228 (222, 234) **
A	Jammu & Kashmir	447 (395, 498)	350 (308, 395)	274 (248, 310) *	237 (224, 255) *	228 (222, 234) **
A	Jharkhand	447 (395, 498)	350 (308, 395)	274 (248, 310) *	237 (224, 255) *	228 (222, 234) **
A	Orissa	447 (395, 498)	350 (308, 395)	274 (248, 310) *	237 (224, 255) *	228 (222, 234) **
A	Rajasthan	447 (395, 498)	350 (308, 395)	274 (248, 310) *	237 (224, 255) *	228 (222, 234) **
A	Uttar Pradesh	447 (395, 498)	350 (308, 395)	274 (248, 310) *	237 (224, 255) *	228 (222, 234) **
A	Uttarakhand	447 (395, 498)	350 (308, 395)	274 (248, 310) *	237 (224, 255) *	228 (222, 234) **
A	Andhra Pradesh	608 (538, 679)	476 (420, 538)	374 (337, 423)	323 (305, 347)	310 (303, 319)
A	Maharashtra	600 (531, 670)	470 (415, 531)	369 (333, 417)	318 (301, 343)	306 (299, 315)
A	Punjab + Chandigarh	675 (597, 753)	529 (466, 597)	415 (374, 469)	358 (338, 385)	344 (336, 354)
A	Delhi	708 (626, 789)	554 (489, 626)	435 (392, 492)	375 (355, 404)	361 (352, 372)
A	Other North Eastern States	734 (649, 819)	575 (507, 649)	451 (407, 511)	390 (368, 419)	374 (365, 386)
A	Madhya Pradesh	773 (684, 863)	606 (534, 684)	475 (429, 538)	410 (388, 441)	394 (385, 406)
A	Karnataka	869 (768, 970)	681 (600, 769)	534 (482, 604)	461 (436, 496)	443 (432, 456)
A	Tamil Nadu + Puducherry	895 (792, 999)	701 (618, 792)	550 (496, 623)	475 (449, 511)	456 (445, 470)
B	India (all states)	690 (623, 759)	570 (520, 627)	464 (404, 523)	366 (315, 423)	302 (279, 329)
B	Low incidence	597 (539, 656)	493 (449, 542)	401 (349, 453)	316 (272, 366)	261 (241, 285) *

B	High incidence	1023 (923, 1124)	845 (770, 928)	687 (598, 775)	542 (467, 627)	447 (413, 487)
B	Kerala + Lakshadweep	487 (439, 535)	402 (367, 442)	327 (285, 369)	258 (222, 298) *	213 (197, 232) **
B	Manipur	538 (486, 592)	445 (405, 489)	362 (315, 408)	285 (246, 330) *	235 (217, 256) *
B	Sikkim	493 (445, 542)	407 (371, 448)	331 (288, 374)	261 (225, 302) *	215 (199, 235) **
B	West Bengal + Andaman & Nicobar Islands	553 (499, 608)	457 (416, 502)	372 (323, 419)	293 (252, 339)	242 (223, 263) *
B	Assam	544 (491, 598)	449 (409, 493)	365 (318, 412)	288 (248, 333) *	238 (220, 259) *
B	Gujarat + Dadra & Nagar Haveli	550 (497, 605)	454 (414, 499)	370 (322, 417)	291 (251, 337)	240 (222, 262) *
B	Bihar	561 (506, 616)	463 (422, 509)	377 (328, 425)	297 (256, 344)	245 (226, 267) *
B	Chhattisgarh	561 (506, 616)	463 (422, 509)	377 (328, 425)	297 (256, 344)	245 (226, 267) *
B	Goa + Daman & Diu	561 (506, 616)	463 (422, 509)	377 (328, 425)	297 (256, 344)	245 (226, 267) *
B	Haryana	561 (506, 616)	463 (422, 509)	377 (328, 425)	297 (256, 344)	245 (226, 267) *
B	Himachal Pradesh	561 (506, 616)	463 (422, 509)	377 (328, 425)	297 (256, 344)	245 (226, 267) *
B	Jammu & Kashmir	561 (506, 616)	463 (422, 509)	377 (328, 425)	297 (256, 344)	245 (226, 267) *
B	Jharkhand	561 (506, 616)	463 (422, 509)	377 (328, 425)	297 (256, 344)	245 (226, 267) *
B	Orissa	561 (506, 616)	463 (422, 509)	377 (328, 425)	297 (256, 344)	245 (226, 267) *
B	Rajasthan	561 (506, 616)	463 (422, 509)	377 (328, 425)	297 (256, 344)	245 (226, 267) *
B	Uttar Pradesh	561 (506, 616)	463 (422, 509)	377 (328, 425)	297 (256, 344)	245 (226, 267) *
B	Uttarakhand	561 (506, 616)	463 (422, 509)	377 (328, 425)	297 (256, 344)	245 (226, 267) *
B	Andhra Pradesh	764 (689, 839)	631 (575, 693)	513 (446, 579)	404 (348, 468)	334 (308, 364)
B	Maharashtra	754 (680, 829)	623 (567, 684)	507 (441, 571)	399 (344, 462)	329 (305, 359)
B	Punjab + Chandigarh	847 (765, 931)	700 (638, 769)	570 (496, 642)	449 (387, 519)	370 (342, 404)
B	Delhi	888 (802, 976)	734 (669, 806)	597 (519, 673)	470 (405, 544)	388 (359, 423)
B	Other North Eastern States	922 (832, 1014)	762 (694, 837)	620 (539, 699)	488 (421, 565)	403 (372, 439)
B	Madhya Pradesh	971 (876, 1067)	802 (731, 881)	652 (568, 736)	514 (443, 595)	424 (392, 462)
B	Karnataka	1091 (985, 1199)	901 (821, 990)	734 (638, 827)	578 (498, 669)	477 (441, 520)
B	Tamil Nadu + Puducherry	1124 (1015, 1236)	929 (846, 1020)	756 (657, 852)	595 (513, 689)	491 (454, 536)
C	India (all states)	754 (686, 823)	645 (585, 706)	548 (494, 604)	448 (388, 511)	376 (335, 420)
C	Low incidence	652 (593, 712)	557 (506, 610)	474 (427, 522)	388 (336, 442)	325 (289, 364)
C	High incidence	1116 (1016, 1219)	955 (866, 1045)	812 (731, 895)	664 (575, 756)	557 (496, 623)
C	Kerala + Lakshadweep	531 (484, 580)	455 (412, 498)	387 (348, 426)	316 (274, 360)	265 (236, 296) *
C	Manipur	587 (535, 641)	502 (456, 550)	428 (385, 471)	349 (303, 398)	293 (261, 328)
C	Sikkim	538 (490, 588)	460 (418, 504)	392 (352, 431)	320 (277, 365)	268 (239, 300) *
C	West Bengal + Andaman & Nicobar Islands	603 (549, 659)	516 (468, 565)	439 (395, 484)	359 (311, 409)	301 (268, 337)
C	Assam	593 (540, 648)	507 (460, 556)	432 (389, 476)	353 (306, 402)	296 (264, 331)
C	Gujarat + Dadra & Nagar Haveli	601 (547, 656)	514 (466, 562)	437 (393, 481)	357 (309, 407)	300 (267, 335)
C	Bihar	612 (557, 668)	523 (475, 573)	445 (401, 490)	364 (315, 415)	305 (272, 341)
C	Chhattisgarh	612 (557, 668)	523 (475, 573)	445 (401, 490)	364 (315, 415)	305 (272, 341)
C	Goa + Daman & Diu	612 (557, 668)	523 (475, 573)	445 (401, 490)	364 (315, 415)	305 (272, 341)
C	Haryana	612 (557, 668)	523 (475, 573)	445 (401, 490)	364 (315, 415)	305 (272, 341)
C	Himachal Pradesh	612 (557, 668)	523 (475, 573)	445 (401, 490)	364 (315, 415)	305 (272, 341)
C	Jammu & Kashmir	612 (557, 668)	523 (475, 573)	445 (401, 490)	364 (315, 415)	305 (272, 341)

C	Jharkhand	612 (557, 668)	523 (475, 573)	445 (401, 490)	364 (315, 415)	305 (272, 341)
C	Orissa	612 (557, 668)	523 (475, 573)	445 (401, 490)	364 (315, 415)	305 (272, 341)
C	Rajasthan	612 (557, 668)	523 (475, 573)	445 (401, 490)	364 (315, 415)	305 (272, 341)
C	Uttar Pradesh	612 (557, 668)	523 (475, 573)	445 (401, 490)	364 (315, 415)	305 (272, 341)
C	Uttarakhand	612 (557, 668)	523 (475, 573)	445 (401, 490)	364 (315, 415)	305 (272, 341)
C	Andhra Pradesh	833 (758, 910)	713 (647, 780)	606 (546, 668)	496 (429, 565)	416 (370, 465)
C	Maharashtra	823 (749, 898)	704 (638, 770)	599 (539, 659)	489 (424, 557)	410 (365, 459)
C	Punjab + Chandigarh	925 (842, 1010)	791 (718, 866)	673 (606, 741)	550 (476, 627)	461 (411, 516)
C	Delhi	969 (882, 1059)	829 (752, 908)	706 (635, 777)	577 (499, 657)	484 (431, 541)
C	Other North Eastern States	1006 (916, 1099)	861 (781, 942)	732 (659, 807)	598 (518, 682)	502 (447, 561)
C	Madhya Pradesh	1059 (964, 1157)	906 (822, 992)	771 (694, 849)	630 (546, 718)	528 (471, 591)
C	Karnataka	1191 (1084, 1300)	1019 (924, 1115)	867 (780, 955)	708 (614, 807)	594 (529, 664)
C	Tamil Nadu + Puducherry	1227 (1117, 1340)	1049 (952, 1149)	893 (804, 983)	730 (632, 831)	612 (545, 684)
D	India (all states)	837 (766, 910)	747 (678, 816)	667 (601, 728)	566 (507, 626)	497 (443, 556)
D	Low incidence	723 (663, 787)	646 (587, 706)	577 (520, 630)	490 (438, 541)	429 (383, 481)
D	High incidence	1239 (1135, 1348)	1106 (1005, 1209)	988 (890, 1079)	839 (751, 927)	735 (657, 823)
D	Kerala + Lakshadweep	590 (540, 642)	527 (479, 576)	470 (424, 514)	399 (357, 441)	350 (313, 392)
D	Manipur	652 (597, 710)	582 (529, 636)	520 (469, 568)	441 (395, 488)	387 (346, 433)
D	Sikkim	597 (547, 650)	533 (485, 583)	476 (429, 520)	404 (362, 447)	355 (317, 397)
D	West Bengal + Andaman & Nicobar Islands	670 (614, 729)	598 (543, 654)	534 (481, 583)	454 (406, 501)	398 (355, 445)
D	Assam	659 (603, 717)	588 (534, 643)	525 (473, 574)	446 (399, 493)	391 (349, 438)
D	Gujarat + Dadra & Nagar Haveli	667 (611, 725)	595 (541, 650)	531 (479, 581)	451 (404, 499)	396 (353, 443)
D	Bihar	679 (622, 739)	606 (551, 663)	541 (488, 592)	460 (412, 508)	403 (360, 451)
D	Chhattisgarh	679 (622, 739)	606 (551, 663)	541 (488, 592)	460 (412, 508)	403 (360, 451)
D	Goa + Daman & Diu	679 (622, 739)	606 (551, 663)	541 (488, 592)	460 (412, 508)	403 (360, 451)
D	Haryana	679 (622, 739)	606 (551, 663)	541 (488, 592)	460 (412, 508)	403 (360, 451)
D	Himachal Pradesh	679 (622, 739)	606 (551, 663)	541 (488, 592)	460 (412, 508)	403 (360, 451)
D	Jammu & Kashmir	679 (622, 739)	606 (551, 663)	541 (488, 592)	460 (412, 508)	403 (360, 451)
D	Jharkhand	679 (622, 739)	606 (551, 663)	541 (488, 592)	460 (412, 508)	403 (360, 451)
D	Orissa	679 (622, 739)	606 (551, 663)	541 (488, 592)	460 (412, 508)	403 (360, 451)
D	Rajasthan	679 (622, 739)	606 (551, 663)	541 (488, 592)	460 (412, 508)	403 (360, 451)
D	Uttar Pradesh	679 (622, 739)	606 (551, 663)	541 (488, 592)	460 (412, 508)	403 (360, 451)
D	Uttarakhand	679 (622, 739)	606 (551, 663)	541 (488, 592)	460 (412, 508)	403 (360, 451)
D	Andhra Pradesh	925 (847, 1006)	826 (750, 903)	737 (665, 806)	626 (560, 692)	549 (490, 615)
D	Maharashtra	913 (837, 994)	815 (741, 891)	728 (656, 795)	618 (553, 683)	542 (484, 607)
D	Punjab + Chandigarh	1027 (940, 1117)	917 (833, 1002)	818 (738, 894)	695 (622, 768)	609 (544, 682)
D	Delhi	1076 (986, 1171)	961 (873, 1050)	858 (773, 937)	729 (652, 805)	639 (570, 715)
D	Other North Eastern States	1117 (1023, 1215)	997 (906, 1090)	890 (803, 973)	756 (677, 836)	663 (592, 742)
D	Madhya Pradesh	1176 (1077, 1279)	1050 (954, 1147)	937 (845, 1024)	796 (712, 880)	698 (623, 781)
D	Karnataka	1322 (1211, 1438)	1180 (1072, 1290)	1054 (950, 1151)	895 (801, 989)	785 (701, 879)
D	Tamil Nadu + Puducherry	1362 (1248, 1482)	1216 (1105, 1329)	1086 (979, 1186)	922 (825, 1019)	809 (722, 905)

E	India (all states)	840 (770, 908)	753 (687, 818)	671 (607, 732)	573 (512, 636)	505 (451, 565)
E	Low incidence	726 (666, 785)	651 (594, 708)	581 (525, 633)	495 (442, 550)	437 (390, 489)
E	High incidence	1244 (1140, 1345)	1115 (1017, 1212)	994 (899, 1083)	848 (758, 942)	748 (669, 837)
E	Kerala + Lakshadweep	592 (543, 640)	531 (485, 577)	473 (428, 516)	404 (361, 449)	356 (318, 399)
E	Manipur	655 (600, 708)	587 (536, 638)	523 (473, 570)	446 (399, 496)	394 (352, 441)
E	Sikkim	600 (550, 649)	538 (491, 584)	479 (434, 522)	409 (365, 454)	360 (322, 404)
E	West Bengal + Andaman & Nicobar Islands	673 (616, 727)	603 (550, 655)	538 (486, 586)	459 (410, 509)	404 (362, 453)
E	Assam	661 (606, 715)	593 (541, 644)	529 (478, 576)	451 (403, 501)	397 (355, 445)
E	Gujarat + Dadra & Nagar Haveli	669 (613, 724)	600 (547, 652)	535 (484, 583)	456 (408, 507)	402 (360, 451)
E	Bihar	682 (625, 737)	611 (558, 665)	545 (493, 594)	465 (415, 517)	410 (367, 459)
E	Chhattisgarh	682 (625, 737)	611 (558, 665)	545 (493, 594)	465 (415, 517)	410 (367, 459)
E	Goa + Daman & Diu	682 (625, 737)	611 (558, 665)	545 (493, 594)	465 (415, 517)	410 (367, 459)
E	Haryana	682 (625, 737)	611 (558, 665)	545 (493, 594)	465 (415, 517)	410 (367, 459)
E	Himachal Pradesh	682 (625, 737)	611 (558, 665)	545 (493, 594)	465 (415, 517)	410 (367, 459)
E	Jammu & Kashmir	682 (625, 737)	611 (558, 665)	545 (493, 594)	465 (415, 517)	410 (367, 459)
E	Jharkhand	682 (625, 737)	611 (558, 665)	545 (493, 594)	465 (415, 517)	410 (367, 459)
E	Orissa	682 (625, 737)	611 (558, 665)	545 (493, 594)	465 (415, 517)	410 (367, 459)
E	Rajasthan	682 (625, 737)	611 (558, 665)	545 (493, 594)	465 (415, 517)	410 (367, 459)
E	Uttar Pradesh	682 (625, 737)	611 (558, 665)	545 (493, 594)	465 (415, 517)	410 (367, 459)
E	Uttarakhand	682 (625, 737)	611 (558, 665)	545 (493, 594)	465 (415, 517)	410 (367, 459)
E	Andhra Pradesh	929 (851, 1004)	832 (760, 905)	742 (672, 809)	633 (566, 703)	558 (499, 625)
E	Maharashtra	917 (840, 991)	822 (750, 893)	733 (663, 799)	625 (558, 694)	551 (493, 617)
E	Punjab + Chandigarh	1031 (945, 1115)	924 (843, 1004)	824 (745, 898)	703 (628, 781)	620 (554, 694)
E	Delhi	1081 (990, 1168)	968 (884, 1053)	864 (781, 941)	737 (658, 818)	649 (581, 727)
E	Other North Eastern States	1122 (1028, 1213)	1005 (917, 1093)	896 (811, 977)	765 (683, 849)	674 (603, 755)
E	Madhya Pradesh	1181 (1082, 1277)	1058 (966, 1151)	944 (854, 1028)	805 (719, 894)	710 (635, 795)
E	Karnataka	1328 (1216, 1435)	1190 (1086, 1293)	1061 (960, 1156)	905 (808, 1005)	798 (714, 894)
E	Tamil Nadu + Puducherry	1368 (1253, 1479)	1226 (1119, 1333)	1093 (989, 1191)	933 (833, 1036)	822 (735, 921)

Table B8. Life-time risk of cervical cancer in the long-term across single-dose protection assumptions
Life-time risk given in cases per 100 000 women born in the long-term, i.e., in the birth cohorts vaccinated 46-50 years since start of vaccination, under 60-100% vaccination coverage.
° See Table A11 for the exact efficacy and rate of waning for the single-dose protection assumptions.
Ŧ States or groups of states as reported in the 2006 National Behaviour Surveillance Survey of the National AIDS Control Organization of India.[9]
¤ See Table A3 for which Indian states are assigned to the clusters of high and low cervical cancer incidence.
§ Other North Eastern States include Arunachal Pradesh, Nagaland, Meghalaya, Mizoram, and Tripura.

Single-dose protection assumption°	Indian state / group of Indian states Ŧ ¤ §	60%	70%	80%	90%	100%
A	India (all states)	438 (387, 489)	343 (303, 388)	269 (243, 305)	232 (220, 250)	223 (218, 230)
A	Low incidence	379 (335, 422)	297 (261, 335)	233 (210, 263)	201 (190, 216)	193 (188, 199)
A	High incidence	650 (575, 726)	510 (449, 575)	400 (361, 452)	345 (326, 371)	331 (324, 342)
A	Kerala + Lakshadweep	303 (268, 338)	238 (209, 268)	186 (168, 211)	161 (152, 173)	155 (151, 159)
A	Manipur	330 (292, 369)	259 (228, 292)	203 (183, 230)	175 (166, 189)	168 (164, 173)
A	Sikkim	337 (298, 376)	264 (233, 298)	207 (187, 234)	179 (169, 192)	172 (168, 177)
A	West Bengal + Andaman & Nicobar Islands	346 (306, 386)	271 (239, 306)	213 (192, 241)	184 (174, 198)	176 (172, 182)
A	Assam	349 (308, 389)	273 (241, 308)	214 (193, 242)	185 (175, 199)	178 (173, 183)
A	Gujarat + Dadra & Nagar Haveli	356 (315, 398)	279 (246, 315)	219 (198, 248)	189 (179, 203)	182 (177, 187)
A	Bihar	357 (316, 399)	280 (247, 316)	220 (198, 249)	190 (179, 204)	182 (178, 188)
A	Chhattisgarh	357 (316, 399)	280 (247, 316)	220 (198, 249)	190 (179, 204)	182 (178, 188)
A	Goa + Daman & Diu	357 (316, 399)	280 (247, 316)	220 (198, 249)	190 (179, 204)	182 (178, 188)
A	Haryana	357 (316, 399)	280 (247, 316)	220 (198, 249)	190 (179, 204)	182 (178, 188)
A	Himachal Pradesh	357 (316, 399)	280 (247, 316)	220 (198, 249)	190 (179, 204)	182 (178, 188)
A	Jammu & Kashmir	357 (316, 399)	280 (247, 316)	220 (198, 249)	190 (179, 204)	182 (178, 188)
A	Jharkhand	357 (316, 399)	280 (247, 316)	220 (198, 249)	190 (179, 204)	182 (178, 188)
A	Orissa	357 (316, 399)	280 (247, 316)	220 (198, 249)	190 (179, 204)	182 (178, 188)
A	Rajasthan	357 (316, 399)	280 (247, 316)	220 (198, 249)	190 (179, 204)	182 (178, 188)
A	Uttar Pradesh	357 (316, 399)	280 (247, 316)	220 (198, 249)	190 (179, 204)	182 (178, 188)
A	Uttarakhand	357 (316, 399)	280 (247, 316)	220 (198, 249)	190 (179, 204)	182 (178, 188)
A	Andhra Pradesh	475 (420, 530)	372 (328, 420)	292 (264, 331)	252 (238, 271)	242 (237, 250)
A	Maharashtra	476 (421, 531)	373 (329, 421)	292 (264, 331)	252 (239, 272)	242 (237, 250)
A	Punjab + Chandigarh	536 (474, 598)	420 (370, 474)	329 (297, 372)	284 (268, 306)	273 (266, 281)
A	Delhi	556 (491, 620)	435 (384, 491)	342 (308, 386)	295 (279, 317)	283 (277, 292)
A	Other North Eastern States	609 (539, 680)	477 (421, 539)	374 (338, 424)	323 (305, 348)	310 (303, 320)
A	Madhya Pradesh	615 (544, 687)	482 (425, 544)	378 (341, 428)	326 (308, 351)	314 (306, 323)
A	Karnataka	685 (606, 764)	537 (473, 606)	421 (380, 476)	363 (343, 391)	349 (341, 360)
A	Tamil Nadu + Puducherry	724 (641, 808)	568 (500, 641)	445 (402, 504)	384 (363, 414)	369 (360, 380)
B	India (all states)	550 (496, 605)	454 (414, 499)	370 (322, 417)	291 (251, 337)	240 (222, 262)
B	Low incidence	475 (429, 522)	393 (358, 431)	320 (278, 360)	252 (217, 291)	208 (192, 226)
B	High incidence	817 (737, 898)	674 (615, 741)	549 (478, 619)	432 (373, 500)	357 (330, 389)
B	Kerala + Lakshadweep	381 (343, 418)	314 (287, 346)	256 (223, 289)	202 (174, 233)	166 (154, 181)

B	Manipur	415 (374, 456)	342 (312, 376)	279 (242, 314)	220 (189, 254)	181 (168, 198)
B	Sikkim	423 (382, 465)	349 (318, 384)	284 (247, 321)	224 (193, 259)	185 (171, 201)
B	West Bengal + Andaman & Nicobar Islands	435 (392, 478)	359 (327, 395)	292 (254, 330)	230 (198, 266)	190 (176, 207)
B	Assam	438 (395, 481)	362 (330, 397)	294 (256, 332)	232 (200, 268)	191 (177, 209)
B	Gujarat + Dadra & Nagar Haveli	447 (404, 492)	370 (337, 406)	301 (262, 339)	237 (204, 274)	196 (181, 213)
B	Bihar	449 (405, 493)	371 (338, 407)	302 (262, 340)	238 (205, 275)	196 (181, 214)
B	Chhattisgarh	449 (405, 493)	371 (338, 407)	302 (262, 340)	238 (205, 275)	196 (181, 214)
B	Goa + Daman & Diu	449 (405, 493)	371 (338, 407)	302 (262, 340)	238 (205, 275)	196 (181, 214)
B	Haryana	449 (405, 493)	371 (338, 407)	302 (262, 340)	238 (205, 275)	196 (181, 214)
B	Himachal Pradesh	449 (405, 493)	371 (338, 407)	302 (262, 340)	238 (205, 275)	196 (181, 214)
B	Jammu & Kashmir	449 (405, 493)	371 (338, 407)	302 (262, 340)	238 (205, 275)	196 (181, 214)
B	Jharkhand	449 (405, 493)	371 (338, 407)	302 (262, 340)	238 (205, 275)	196 (181, 214)
B	Orissa	449 (405, 493)	371 (338, 407)	302 (262, 340)	238 (205, 275)	196 (181, 214)
B	Rajasthan	449 (405, 493)	371 (338, 407)	302 (262, 340)	238 (205, 275)	196 (181, 214)
B	Uttar Pradesh	449 (405, 493)	371 (338, 407)	302 (262, 340)	238 (205, 275)	196 (181, 214)
B	Uttarakhand	449 (405, 493)	371 (338, 407)	302 (262, 340)	238 (205, 275)	196 (181, 214)
B	Andhra Pradesh	597 (539, 656)	493 (449, 542)	401 (349, 452)	316 (272, 366)	261 (241, 284)
B	Maharashtra	597 (539, 657)	493 (450, 542)	402 (349, 453)	316 (273, 366)	261 (241, 285)
B	Punjab + Chandigarh	672 (607, 739)	555 (506, 610)	452 (393, 510)	356 (307, 412)	294 (272, 320)
B	Delhi	698 (630, 767)	576 (525, 633)	469 (408, 529)	370 (318, 428)	305 (282, 333)
B	Other North Eastern States	765 (690, 841)	632 (576, 694)	514 (447, 580)	405 (349, 469)	334 (309, 364)
B	Madhya Pradesh	772 (697, 849)	638 (581, 701)	519 (452, 586)	409 (352, 473)	338 (312, 368)
B	Karnataka	860 (776, 945)	710 (647, 781)	578 (503, 652)	455 (392, 527)	376 (347, 410)
B	Tamil Nadu + Puducherry	910 (821, 1000)	751 (685, 826)	611 (532, 689)	482 (415, 557)	397 (367, 433)
C	India (all states)	600 (546, 656)	514 (466, 562)	437 (393, 481)	357 (309, 407)	300 (267, 335)
C	Low incidence	519 (472, 566)	444 (403, 486)	378 (340, 416)	309 (267, 351)	259 (230, 289)
C	High incidence	891 (811, 973)	762 (692, 835)	649 (584, 714)	530 (459, 604)	445 (396, 497)
C	Kerala + Lakshadweep	415 (378, 454)	355 (322, 389)	302 (272, 333)	247 (214, 281)	207 (185, 232)
C	Manipur	453 (412, 494)	387 (351, 424)	329 (296, 363)	269 (233, 307)	226 (201, 252)
C	Sikkim	461 (420, 504)	395 (358, 432)	336 (302, 370)	274 (238, 313)	230 (205, 257)
C	West Bengal + Andaman & Nicobar Islands	474 (432, 518)	406 (368, 444)	345 (311, 380)	282 (244, 321)	237 (211, 265)
C	Assam	478 (435, 522)	409 (371, 447)	348 (313, 383)	284 (246, 324)	238 (212, 267)
C	Gujarat + Dadra & Nagar Haveli	488 (444, 533)	418 (379, 457)	355 (320, 391)	290 (252, 331)	244 (217, 272)
C	Bihar	490 (446, 535)	419 (380, 459)	356 (321, 393)	291 (252, 332)	244 (218, 273)
C	Chhattisgarh	490 (446, 535)	419 (380, 459)	356 (321, 393)	291 (252, 332)	244 (218, 273)
C	Goa + Daman & Diu	490 (446, 535)	419 (380, 459)	356 (321, 393)	291 (252, 332)	244 (218, 273)
C	Haryana	490 (446, 535)	419 (380, 459)	356 (321, 393)	291 (252, 332)	244 (218, 273)
C	Himachal Pradesh	490 (446, 535)	419 (380, 459)	356 (321, 393)	291 (252, 332)	244 (218, 273)
C	Jammu & Kashmir	490 (446, 535)	419 (380, 459)	356 (321, 393)	291 (252, 332)	244 (218, 273)
C	Jharkhand	490 (446, 535)	419 (380, 459)	356 (321, 393)	291 (252, 332)	244 (218, 273)
C	Orissa	490 (446, 535)	419 (380, 459)	356 (321, 393)	291 (252, 332)	244 (218, 273)

C	Rajasthan	490 (446, 535)	419 (380, 459)	356 (321, 393)	291 (252, 332)	244 (218, 273)
C	Uttar Pradesh	490 (446, 535)	419 (380, 459)	356 (321, 393)	291 (252, 332)	244 (218, 273)
C	Uttarakhand	490 (446, 535)	419 (380, 459)	356 (321, 393)	291 (252, 332)	244 (218, 273)
C	Andhra Pradesh	651 (593, 711)	557 (506, 610)	474 (427, 522)	387 (336, 441)	325 (289, 363)
C	Maharashtra	652 (593, 712)	558 (506, 611)	475 (427, 523)	388 (336, 442)	325 (290, 364)
C	Punjab + Chandigarh	734 (668, 801)	628 (570, 687)	534 (481, 588)	436 (378, 497)	366 (326, 409)
C	Delhi	762 (693, 832)	651 (591, 713)	554 (499, 610)	453 (392, 516)	380 (338, 425)
C	Other North Eastern States	835 (760, 911)	714 (648, 782)	607 (547, 669)	496 (430, 566)	416 (371, 466)
C	Madhya Pradesh	843 (767, 921)	721 (654, 790)	614 (552, 676)	501 (434, 571)	421 (375, 470)
C	Karnataka	938 (854, 1025)	803 (728, 879)	683 (615, 752)	558 (484, 636)	468 (417, 524)
C	Tamil Nadu + Puducherry	993 (903, 1084)	849 (770, 930)	722 (650, 796)	590 (511, 673)	495 (441, 554)
D	India (all states)	667 (611, 725)	595 (541, 650)	531 (479, 581)	451 (404, 499)	396 (353, 443)
D	Low incidence	576 (528, 627)	514 (467, 562)	459 (414, 501)	390 (349, 431)	342 (305, 383)
D	High incidence	989 (906, 1076)	883 (802, 965)	789 (711, 862)	670 (599, 740)	587 (524, 657)
D	Kerala + Lakshadweep	461 (422, 502)	412 (374, 450)	368 (331, 402)	312 (279, 345)	274 (244, 306)
D	Manipur	502 (460, 547)	448 (407, 490)	400 (361, 437)	340 (304, 376)	298 (266, 334)
D	Sikkim	512 (469, 557)	457 (415, 500)	408 (368, 446)	347 (310, 383)	304 (271, 340)
D	West Bengal + Andaman & Nicobar Islands	527 (482, 573)	470 (427, 514)	420 (379, 459)	357 (319, 394)	313 (279, 350)
D	Assam	530 (486, 577)	474 (430, 518)	423 (381, 462)	359 (321, 397)	315 (281, 352)
D	Gujarat + Dadra & Nagar Haveli	542 (497, 590)	484 (440, 529)	432 (390, 472)	367 (328, 406)	322 (287, 360)
D	Bihar	544 (498, 592)	485 (441, 530)	433 (391, 473)	368 (329, 407)	323 (288, 361)
D	Chhattisgarh	544 (498, 592)	485 (441, 530)	433 (391, 473)	368 (329, 407)	323 (288, 361)
D	Goa + Daman & Diu	544 (498, 592)	485 (441, 530)	433 (391, 473)	368 (329, 407)	323 (288, 361)
D	Haryana	544 (498, 592)	485 (441, 530)	433 (391, 473)	368 (329, 407)	323 (288, 361)
D	Himachal Pradesh	544 (498, 592)	485 (441, 530)	433 (391, 473)	368 (329, 407)	323 (288, 361)
D	Jammu & Kashmir	544 (498, 592)	485 (441, 530)	433 (391, 473)	368 (329, 407)	323 (288, 361)
D	Jharkhand	544 (498, 592)	485 (441, 530)	433 (391, 473)	368 (329, 407)	323 (288, 361)
D	Orissa	544 (498, 592)	485 (441, 530)	433 (391, 473)	368 (329, 407)	323 (288, 361)
D	Rajasthan	544 (498, 592)	485 (441, 530)	433 (391, 473)	368 (329, 407)	323 (288, 361)
D	Uttar Pradesh	544 (498, 592)	485 (441, 530)	433 (391, 473)	368 (329, 407)	323 (288, 361)
D	Uttarakhand	544 (498, 592)	485 (441, 530)	433 (391, 473)	368 (329, 407)	323 (288, 361)
D	Andhra Pradesh	723 (662, 787)	646 (587, 706)	576 (520, 630)	490 (438, 541)	429 (383, 481)
D	Maharashtra	724 (663, 787)	646 (587, 706)	577 (520, 630)	490 (438, 541)	430 (384, 481)
D	Punjab + Chandigarh	815 (746, 886)	727 (661, 795)	649 (586, 709)	552 (494, 610)	484 (432, 541)
D	Delhi	846 (774, 920)	755 (686, 825)	674 (608, 736)	572 (512, 633)	502 (448, 562)
D	Other North Eastern States	927 (849, 1008)	827 (751, 904)	739 (666, 807)	627 (561, 693)	550 (491, 616)
D	Madhya Pradesh	936 (857, 1018)	836 (759, 913)	746 (673, 815)	634 (567, 700)	556 (496, 622)
D	Karnataka	1042 (954, 1134)	930 (845, 1017)	830 (749, 907)	705 (631, 779)	618 (552, 692)
D	Tamil Nadu + Puducherry	1102 (1009, 1199)	984 (894, 1075)	878 (792, 960)	746 (668, 824)	654 (584, 732)
E	India (all states)	669 (613, 724)	600 (547, 652)	535 (484, 583)	456 (408, 507)	402 (360, 451)
E	Low incidence	578 (530, 625)	518 (473, 563)	462 (418, 504)	394 (352, 438)	347 (311, 389)

E	High incidence	994 (910, 1074)	890 (813, 968)	794 (718, 865)	677 (605, 752)	597 (534, 669)
E	Kerala + Lakshadweep	463 (424, 501)	415 (379, 451)	370 (335, 403)	316 (282, 351)	278 (249, 312)
E	Manipur	504 (462, 545)	452 (413, 492)	403 (365, 439)	344 (307, 382)	303 (271, 340)
E	Sikkim	514 (471, 556)	461 (421, 501)	411 (372, 448)	351 (313, 390)	309 (276, 346)
E	West Bengal + Andaman & Nicobar Islands	529 (485, 572)	474 (433, 515)	423 (382, 461)	361 (322, 401)	318 (284, 356)
E	Assam	533 (488, 576)	477 (436, 519)	426 (385, 464)	363 (324, 403)	320 (286, 359)
E	Gujarat + Dadra & Nagar Haveli	544 (499, 588)	488 (445, 530)	435 (394, 474)	371 (331, 412)	327 (293, 366)
E	Bihar	546 (500, 590)	489 (447, 532)	436 (395, 475)	372 (332, 413)	328 (293, 367)
E	Chhattisgarh	546 (500, 590)	489 (447, 532)	436 (395, 475)	372 (332, 413)	328 (293, 367)
E	Goa + Daman & Diu	546 (500, 590)	489 (447, 532)	436 (395, 475)	372 (332, 413)	328 (293, 367)
E	Haryana	546 (500, 590)	489 (447, 532)	436 (395, 475)	372 (332, 413)	328 (293, 367)
E	Himachal Pradesh	546 (500, 590)	489 (447, 532)	436 (395, 475)	372 (332, 413)	328 (293, 367)
E	Jammu & Kashmir	546 (500, 590)	489 (447, 532)	436 (395, 475)	372 (332, 413)	328 (293, 367)
E	Jharkhand	546 (500, 590)	489 (447, 532)	436 (395, 475)	372 (332, 413)	328 (293, 367)
E	Orissa	546 (500, 590)	489 (447, 532)	436 (395, 475)	372 (332, 413)	328 (293, 367)
E	Rajasthan	546 (500, 590)	489 (447, 532)	436 (395, 475)	372 (332, 413)	328 (293, 367)
E	Uttar Pradesh	546 (500, 590)	489 (447, 532)	436 (395, 475)	372 (332, 413)	328 (293, 367)
E	Uttarakhand	546 (500, 590)	489 (447, 532)	436 (395, 475)	372 (332, 413)	328 (293, 367)
E	Andhra Pradesh	726 (665, 785)	651 (594, 708)	580 (525, 632)	495 (442, 550)	436 (390, 489)
E	Maharashtra	727 (666, 786)	651 (594, 708)	581 (525, 633)	496 (443, 550)	437 (391, 489)
E	Punjab + Chandigarh	818 (750, 884)	733 (669, 797)	654 (591, 712)	558 (498, 620)	492 (440, 551)
E	Delhi	849 (778, 918)	761 (694, 827)	678 (614, 739)	579 (517, 643)	510 (456, 571)
E	Other North Eastern States	930 (852, 1006)	834 (761, 907)	744 (673, 810)	634 (567, 705)	559 (500, 626)
E	Madhya Pradesh	940 (861, 1016)	842 (769, 916)	751 (680, 818)	641 (572, 712)	565 (505, 633)
E	Karnataka	1046 (959, 1131)	937 (856, 1019)	836 (756, 911)	713 (637, 792)	629 (562, 704)
E	Tamil Nadu + Puducherry	1107 (1014, 1196)	992 (905, 1078)	884 (800, 964)	754 (674, 838)	665 (595, 745)

Table B9. Relative reduction in life-time risk of cervical cancer by maximum age of catch-up

Max. age of catch-up	Coverage of catch-up	% Relative reduction in life-time risk of cervical cancer by age at the introduction of vaccination				
		11-30 yrs (unstratified)	11-15 yrs	16-20 yrs	21-25 yrs	25-30 yrs
NA (base-case)	NA	3% (2, 4)	7% (5, 10)	4% (2, 5)	2% (1, 2)	0% (0, 1)
15 yrs	60%	13% (12, 14)	44% (41, 47)	5% (3, 7)	2% (1, 4)	1% (0, 1)
15 yrs	90%	18% (17, 19)	61% (59, 64)	6% (4, 8)	3% (2, 4)	1% (0, 2)
20 yrs	60%	23% (21, 24)	50% (47, 54)	34% (31, 37)	4% (3, 6)	2% (1, 3)
20 yrs	90%	31% (30, 32)	69% (66, 71)	47% (44, 50)	5% (4, 7)	3% (2, 3)
25 yrs	60%	29% (27, 30)	52% (48, 56)	39% (36, 42)	21% (19, 24)	3% (2, 4)
25 yrs	90%	39% (38, 40)	70% (67, 72)	53% (51, 56)	30% (27, 33)	4% (3, 5)
30 yrs	60%	33% (31, 35)	53% (50, 57)	40% (37, 43)	25% (22, 28)	14% (12, 16)
30 yrs	90%	45% (43, 46)	71% (68, 73)	54% (51, 57)	35% (32, 38)	20% (17, 22)

B.2. Supplementary results in figures

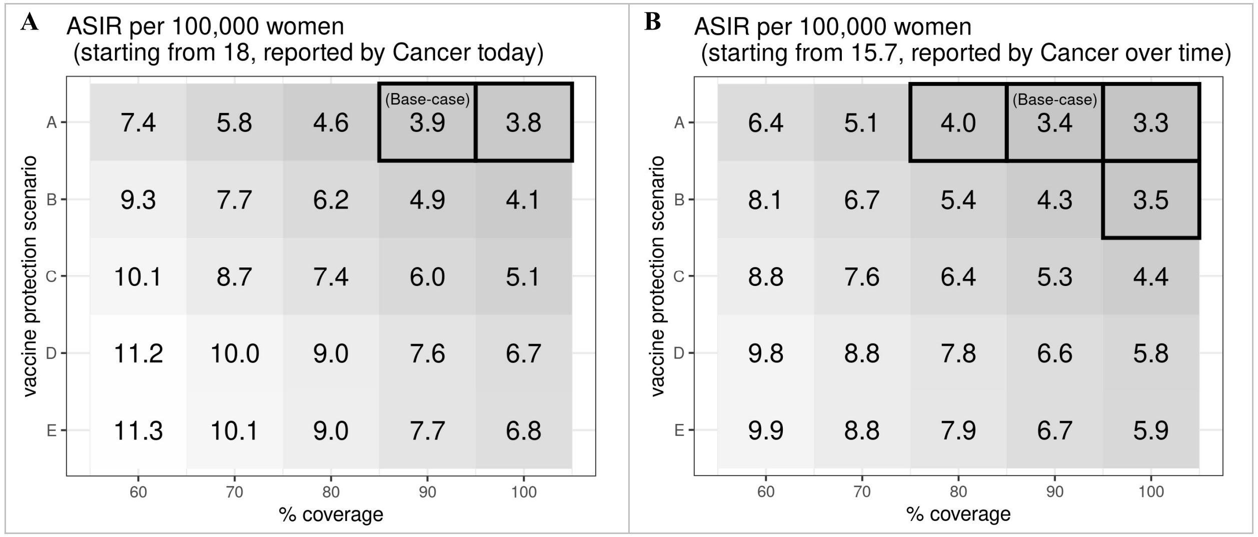

Figure B1. Age-standardised incidence rate of cervical cancer under alternative baseline values.
Age-standardised incidence rate (ASIR) of cervical cancer per 100 000 women-years by vaccine protection assumption (rows in each panel) and % coverage (columns in each panel) in the long term, i.e., in birth cohorts vaccinated 46-50 years after start of vaccination. Assumption A also represents life-long protection for two-dose schedule. Thick outlines indicate being below the elimination threshold of 4 cases per 100 000 women-years. Panel A: starting from baseline ASIR of 18 cases per 100 000 women-years according to Cancer Today of GLOBOCAN,[25] derived by extrapolation of incidence across India based on urban/rural entity. This estimate of baseline ASIR was also used by Drolet et al.[26] Panel B: starting from baseline ASIR of 15.7 cases per 100 000 women-years according to Cancer over time of GLOBOCAN,[27] derived based on a selection of cancer registries, without applying extrapolation.[3] This estimate of baseline ASIR was also used by Brisson et al.[28] Settings fixed across all scenarios: routine vaccination in 10 year-old girls without catch-up vaccination.

Reference

1. Baussano I, Elfström KM, Lazzarato F, et al. Type-specific human papillomavirus biological features: Validated model-based estimates. *PLoS ONE* 2013; **8**(11): e81171.
2. Jenness SM, Morris M. EpiModel: An R Package for Mathematical Modeling of Infectious Disease over Networks. *Journal of Statistical Software* 2018; **84**(8): 1-47.
3. Stelzle D, Tanaka LF, Lee KK, et al. Estimates of the global burden of cervical cancer associated with HIV. *The Lancet Global Health* 2020; **9**(2): e161-e9.
4. Berkhof J, Bogaards JA, Demirel E, Diaz M, Sharma M, Kim JJ. Cost-Effectiveness of Cervical Cancer Prevention in Central and Eastern Europe and Central Asia. *Vaccine* 2013; **31**(S7): H71-H9.
5. Bogaards JA, Xiridou M, Coupé VMH, Meijer CJLM, Wallinga J, Berkhof J. Model-based estimation of viral transmissibility and infection-induced resistance from the age-dependent prevalence of infection for 14 high-risk types of human papillomavirus. *American Journal of Epidemiology* 2010; **171**(7): 817-25.
6. Man I, Georges D, Bonjour M, Baussano I. " Footprinting" missing epidemiological data for cervical cancer: a case study in India. *medRxiv* 2022.
7. Bray F, Colombet M, Mery L, et al. Cancer Incidence in Five Continents, Vol. XI (electronic version). Lyon: International Agency for Research on Cancer. 2017. http://ci5.iarc.fr (accessed 1 February 2021).
8. Report of National Cancer Registry Programme 2012-2016. National Centre for Disease Informatics and Research. 2020. https://www.ncdirindia.org/All_Reports/Report_2020/resources/NCRP_2020_2012_16.pdf (accessed 1 October 2021).
9. National Behavioural Surveillance Survey: General Population 2006. National AIDS Control Organisation Ministry of Health and Family Welfare Government of India. https://www.aidsdatahub.org/sites/default/files/resource/national-bss-general-population-india-2006.pdf (accessed 1 February 2021).
10. Franceschi S, Rajkumar R, Snijders PJF, et al. Papillomavirus infection in rural women in southern India. *British Journal of Cancer* 2005; **92**(3): 601-6.
11. Dutta S, Begum R, Mazumder D, et al. Prevalence of Human Papillomavirus in Women Without Cervical Cancer: A Population-based Study in Eastern India. *International Journal of Gynecological Pathology* 2012; **31**(2): 178-83.
12. The Demographic and Health Surveys (DHS). United States Agency of International Development (USAID). https://dhsprogram.com/ (accessed 1 October 2021).
13. Gaffey MF, Venkatesh S, Dhingra N, et al. Male use of female sex work in India: A nationally representative behavioural survey. *PLoS One* 2011; **6**(7): e22704.
14. Vandepitte J, Lyerla R, Dallabetta G, Crabbé F, Alary M, Buvé A. Estimates of the number of female sex workers in different regions of the world. *Sexually Transmitted Infections* 2006; **82**(suppl 3): iii18-25.
15. Basu P, Chandna P, Bamezai RNK, et al. MassARRAY spectrometry is more sensitive than PreTect HPV-Proofer and consensus PCR for type-specific detection of high-risk oncogenic human papillomavirus genotypes in cervical cancer. *Journal of Clinical Microbiology* 2011; **49**(10): 3537-44.
16. Bonjour M, Charvat H, Franco EL, et al. Global estimates of expected and preventable cervical cancers among girls born between 2005 and 2014: a birth cohort analysis. *The Lancet Public Health* 2021; **6**(7): e510-21.
17. UN Life tables. United Nations Department of Economic Social Affairs Population Dynamics. https://population.un.org/wpp/Download (accessed 1 February 2021).
18. Segi M. Cancer mortality for selected sites in 24 countries 1950-57. *Sendai, Japan: Department of Public Health, Tohoku University of Medicine* 1960.
19. India Census C-series 2011. Office of the Registrar, General Census Commissioner India. https://censusindia.gov.in/2011census/C-series/C-13.html (accessed 1 February 2021).
20. Basu P, Malvi SG, Joshi S, et al. Vaccine efficacy against persistent human papillomavirus (HPV) 16/18 infection at 10 years after one, two, and three doses of quadrivalent HPV vaccine in girls in India: a multicentre, prospective, cohort study. *The Lancet Oncology* 2021; **22**(11): 1518-29.
21. Panicker G, Rajbhandari I, Pathak HN, Brady AM, Unger ER. Multiplex immunoassay to measure antibody response to nine HPV vaccine types. *Journal of Immunological Methods* 2021; **498**: 113136.
22. Canfell K, Kim JJ, Kulasingam S, et al. HPV-FRAME: A consensus statement and quality framework for modelled evaluations of HPV-related cancer control. *Papillomavirus Research* 2019; **8**: 100184.
23. Bruni L, Serrano B, Roura E, et al. Cervical cancer screening programmes and age-specific coverage estimates for 202 countries and territories worldwide: a review and synthetic analysis. *The Lancet Global Health* 2022; **10**(8): e1115-e27.
24. Jit M, Prem K, Benard E, Brisson M. From cervical cancer elimination to eradication of vaccine-type human papillomavirus: Feasibility, public health strategies and cost-effectiveness. *Preventive Medicine* 2021; **144**: 106354.
25. Ferlay J, Ervik M, Lam F, et al. Global Cancer Observatory: Cancer Today. Lyon, France: International Agency for Research on Cancer. 2020. https://gco.iarc.fr/today (accessed 15 February 2022).

26. Drolet M, Laprise JF, Martin D, et al. Optimal human papillomavirus vaccination strategies to prevent cervical cancer in low-income and middle-income countries in the context of limited resources: a mathematical modelling analysis. *The Lancet Infectious Diseases* 2021; **21**(11): 1598-610.
27. Ervik M, Lam F, Laversanne M, Ferlay J, Bray F. Global Cancer Observatory: Cancer Over Time. Lyon, France: International Agency for Research on Cancer. 2020. https://gco.iarc.fr/overtime (accessed 15 February 2022).
28. Brisson M, Kim JJ, Canfell K, et al. Impact of HPV vaccination and cervical screening on cervical cancer elimination: a comparative modelling analysis in 78 low-income and lower-middle-income countries. *The Lancet* 2020; **395**(10224): 575-90.
